# Solution-processable polymer membranes with hydrophilic subnanometre pores for sustainable lithium extraction

**DOI:** 10.1038/s44221-025-00398-8

**Published:** 2025-03-12

**Authors:** Dingchang Yang, Yijie Yang, Toby Wong, Sunshine Iguodala, Anqi Wang, Louie Lovell, Fabrizia Foglia, Peter Fouquet, Charlotte Breakwell, Zhiyu Fan, Yanlin Wang, Melanie M. Britton, Daryl R. Williams, Nilay Shah, Tongwen Xu, Neil B. McKeown, Maria-Magdalena Titirici, Kim E. Jelfs, Qilei Song

**Affiliations:** 1 Department of Chemical Engineering, Imperial College London, London, UK; 2 Department of Chemistry, Molecular Science Research Hub, Imperial College London, London, UK; 3 School of Chemistry, University of Birmingham, Birmingham, UK; 4 Department of Chemistry, University College London, London, UK; 5 Institut Laue-Langevin, Grenoble, France; 6 Key Laboratory of Precision and Intelligent Chemistry, School of Chemistry and Materials Science, University of Science and Technology of China, Hefei, People’s Republic of China; 7 EaStCHEM, School of Chemistry, University of Edinburgh, Edinburgh, UK

**Keywords:** Porous materials, Polymer chemistry, Polymers, Chemical engineering, Materials chemistry

## Abstract

Membrane-based separation processes hold great promise for sustainable extraction of lithium from brines for the rapidly expanding electric vehicle industry and renewable energy storage. However, it remains challenging to develop high-selectivity membranes that can be upscaled for industrial processes. Here we report solution-processable polymer membranes with subnanometre pores with excellent ion separation selectivity in electrodialysis processes for lithium extraction. Polymers of intrinsic microporosity incorporated with hydrophilic functional groups enable fast transport of monovalent alkali cations (Li^+^, Na^+^ and K^+^) while rejecting relatively larger divalent ions such as Mg^2+^. The polymer of intrinsic microporosity membranes surpasses the performance of most existing membrane materials. Furthermore, the membranes were scaled up and integrated into an electrodialysis stack, demonstrating excellent selectivity in simulated salt-lake brines. This work will inspire the development of selective membranes for a wide range of sustainable separation processes critical for resource recovery and a global circular economy.

## Abstract (web summary)

The microporous polymer membranes with the introduction of hydrophilic functional groups achieve high selectivity for monovalent/divalent ion separation in electrodialysis processes, providing an alternative approach to lithium extraction with easy scale-up.

## Main

**Fig. 1:**
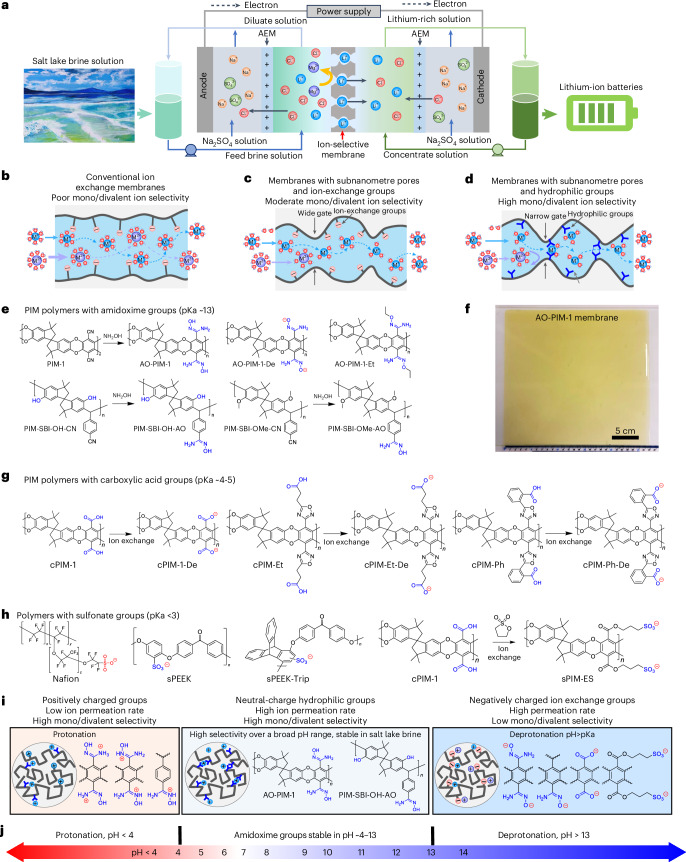
Ion-selective membrane-based electrodialysis processes for lithium extraction. **a**, Schematic diagram of selective electrodialysis process for lithium extraction from brine solution. For simplicity, only one ion-selective membrane (ISM) and two anion exchange membranes (AEMs) are shown in the diagram. **b**, Schematic diagram of ion transport in conventional ion-exchange membranes. **c**, Schematic diagram of selective ion transport in ion-exchange membranes with subnanometre channels. **d**, Schematic diagram of selective ion transport in microporous ion-selective membranes with hydrophilic functional groups. **e**, Chemical structures of hydrophilic PIM polymers with amidoxime groups. **f**, Photo of AO-PIM-1 membrane with size of 25 cm × 27 cm, exposed in water. **g**, PIM polymers with carboxylic acid and carboxylate groups. **h**, Polymers with sulfonate groups. **i**, Summary of polymer membranes in different charge states. **j**, Schematic diagram showing the operation of AO-PIM membranes over a broad pH range.

With the growing global demand for lithium used in lithium-ion batteries for electric vehicles and renewable energy storage, there is an urgent need for recycling of lithium and extraction from unconventional sources^1^. Traditional lithium extraction methods, such as hard rock mining, face environmental challenges and limitations in scalability. Direct lithium extraction from various water resources, such as salt-lake brines or geothermal brine solutions, offer a promising alternative to enhance efficiency, reduce environmental impact and address economic considerations^2–8^. A wide range of materials and direct lithium extraction technologies have been developed^4^, such as adsorbents and membrane-based separation processes including reverse osmosis and nanofiltration for lithium extraction^9^. Selective electrodialysis has emerged as a sustainable and efficient lithium extraction technology for extracting lithium ions from brine solutions^10,11^. In electrodialysis processes, lithium ions selectively transport through an ion-selective membrane under the electric field, resulting in efficient separation from other ions present in the brine (Fig. 1a). Furthermore, electrodialysis processes can be driven by the renewable electricity and combined with adsorption, reverse osmosis and nanofiltration processes, especially for processing high-concentration brine solutions^12^. One key scientific challenge lies in the development of ion-selective membranes that provide high selectivity of monovalent ions towards divalent ions present in complex brine solutions^13^.

Designing high-selectivity membranes with precise separation of solutes has broad implications for a wide range of industrial processes^14–17^, for example, desalination, ion separation and extraction, resource recovery and recycling, and electrochemical energy conversion and storage. The ion transport through a membrane with subnanometre pores could be governed by several mechanisms, including size and Donnan exclusion, ion dehydration and intrapore ion diffusion. A variety of parameters could determine the ion transport, such as channel size, surface chemistry and charges, hydration, interactions between ion and pore walls and confinement environments. Traditional water desalination membranes^18^ or nanofiltration membranes for ion separation^19^ have dense structures or disordered pores with moderate ion–ion selectivity between nearly identical hydrated diameter of ions with different valency, such as Li^+^ (hydrated diameter 7.64 Å) and Mg^2+^ ions (8.56 Å) (ref. ^20^). Conventional ion-exchange membranes are usually made of polymers with high chain mobility and phase separation, leading to large ion channels with poor ion selectivity (typically below 10) (Fig. 1b). Next generation of ion separation membranes have been developed from microporous materials with ordered confined channels^21,22^, such as metal–organic frameworks^23,24^, covalent organic frameworks^25–27^, porous organic cages^28^ and two-dimensional materials such as graphene^29^ and MoS_2_ nanosheets^30^. The difficulty of manufacturing and implementing these advanced materials into defect-free ion-selective membranes can be a limiting factor for their widespread adoption, given the scale of operations that would be required for industrially relevant processes.

Polymers of intrinsic microporosity (PIMs) are a class of polymers with rigid and contorted backbone structures, forming interconnected hourglass-shaped size-selective channels that enable efficient molecular sieving^31–34^, ion separation^35^ and selective ion transport in redox flow batteries^35–40^. Compared to other crystalline microporous materials, PIM polymers are soluble in common solvents and can be easily processed into membranes, which allows easy upscaling and manufacturing using industrial continuous roll-to-roll membrane casting lines. Given the promising features of PIM membranes, we hypothesize that they would provide high mono/divalent ion selectivity in electrodialysis processes for direct lithium extraction. However, PIM membranes incorporated with ion-exchange groups tend to swell upon hydration, potentially leading to moderate selectivity for mono/divalent ion separation (Fig. 1c).

In this study, we report the development of PIM membranes with hydrophilic functional groups as ion-selective membranes and demonstrate their excellent monovalent/divalent selectivity for efficient lithium extraction through selective electrodialysis processes (Fig. 1d). The hydrophilic PIM membranes consist of rigid and contorted polymer chains incorporated with hydrophilic functional groups such as amidoxime (Fig. 1e). These polymers are solution processable and can be cast into large-area membranes (Fig. 1f). Generally, polymers with hydrophilic groups formed rigid hydrogen-bonded networks through both interchain and intrachain interactions, which further enhance the chain rigidity. The resulting narrow subnanometre-sized ion channels restrict the ion partitioning through dehydration process and confined diffusion within the channels. Furthermore, introducing hydrophilic groups into the interconnected subnanometre pores generated ion–pore interactions that play important roles in regulating ion transport. Polymer membranes with carboxylic acid groups (Fig. 1g) and sulfonate groups (Fig. 1h) were also developed and their varied ability of ionization led to slightly different ion–pore interactions. Ion-exchangeable groups with a lower p*K*_a_ (for example carboxylic acid at ~4–5) can dissociate partially in neutral pH, which can be carefully designed to reduce swelling and achieve high ion selectivity. Membranes with sulfonate groups dissociate completely in neutral pH, leading to strong electrostatic interactions and consequently poor monovalent/divalent selectivity. The amidoxime groups interact preferentially with alkali metal cations and improve selectivity towards divalent ions over a broad pH range (Fig. 1i,j). Owing to the synergistic effect of size sieving, regulated ion dehydration, electrostatic interactions and restricted intrapore diffusion, hydrophilic PIM membranes enable fast transport of smaller monovalent alkali metal cations (Li^+^, Na^+^ and K^+^) under the driving force of an electric field, while effectively rejecting relatively larger divalent ions such as Mg^2+^. This work will inspire the development of ion-selective membranes and advance electrodialysis processes for a diverse range of strategically important separation applications.

## Design and characterization of polymer membranes

We developed amidoxime-functionalized PIM membranes using two generations of PIM polymers, including the first generation of dibenzodioxin-based PIMs, PIM-1 (Supplementary Fig. 1) and a new generation of ether-bond-free PIMs prepared by superacid-catalysed Friedel–Crafts reactions (Supplementary Fig. 2). The nitrile groups in PIM-1 polymer can be readily modified into hydrophilic functional groups including amidoxime (AO-PIM-1)^41^ and carboxylic acid (cPIM-1)^42,43^. Following our previous work^35,36^, AO-PIM-1 polymer was exposed to alkaline solutions at a high pH of 14 to deprotonate the hydroxyl groups, forming a negatively charged polymer AO-PIM-1-De. We also substituted the hydroxyl groups in the AO-PIM-1 with non-polar hydrophobic ethyl groups (AO-PIM-1-Et) while keeping the hydrophilic amine groups. Amidoxime-functionalized polyacrylonitrile (AO-PAN) membranes with varied degree of modification were prepared as control samples with negligible microporosity in the solid state. Moreover, AO-PIM polymers with more rigid backbones were also prepared, including AO-PIM-SBF (spirobifluorene) and AO-PIM-DBMP (dibenzomethanopentacene) membranes. Furthermore, we synthesized another group of PIM polymers by superacid-catalysed Friedel–Crafts reactions, including PIM-SBI-OH-CN and PIM-SBI-OMe-CN with varied ratio of hydroxyl groups and protection by methyl groups, respectively. The nitrile groups of these two polymers were also modified to amidoxime groups (PIM-SBI-OH-AO and PIM-SBI-OMe-AO).

To explore the effect of other functional groups on ion separation, we also prepared PIM polymers with carboxylic acid groups and variants (Fig. 1g and Supplementary Fig. 3), including cPIM-1 by acid hydrolysis of PIM-1 and substituted with methyl groups (cPIM-1-OMe), cPIMs with oxadiazole and ethyl- (cPIM-Et), and phenyl- (cPIM-Ph) pendant groups, following the protocol reported in our recent work^40^. Polymer membranes with sulfonate groups were also included as control samples (Fig. 1h and Supplementary Fig. 4), including Nafion, sulfonated poly (ether-ether-ketone) (sPEEK), sPEEK with triptycene backbone (sPEEK-Trip)^44,45^ and sulfonated PIM polymers via esterification modification following a previous study^46^. The molecular engineering of these polymers allows us to tailor the membrane pore size, functional groups and understand their roles in governing the water–ion–membrane interactions and ion transport dynamics.

The polymer structures were characterized by nuclear magnetic resonance (NMR) (Supplementary Figs. 5–8) and Fourier transform infrared (FTIR) spectroscopy (Supplementary Fig. 9). Most of the polymers are soluble in polar solvents such as dimethylformamide and dimethyl sulfoxide (DMSO) and can be easily cast into membranes (Supplementary Fig. 10). Typically, the membrane thickness is between 30 and 70 µm. Scanning electron microscopy images of the membrane cross sections confirm that the membranes are dense films without defects (Supplementary Figs. 11 and 12).

**Fig. 2:**
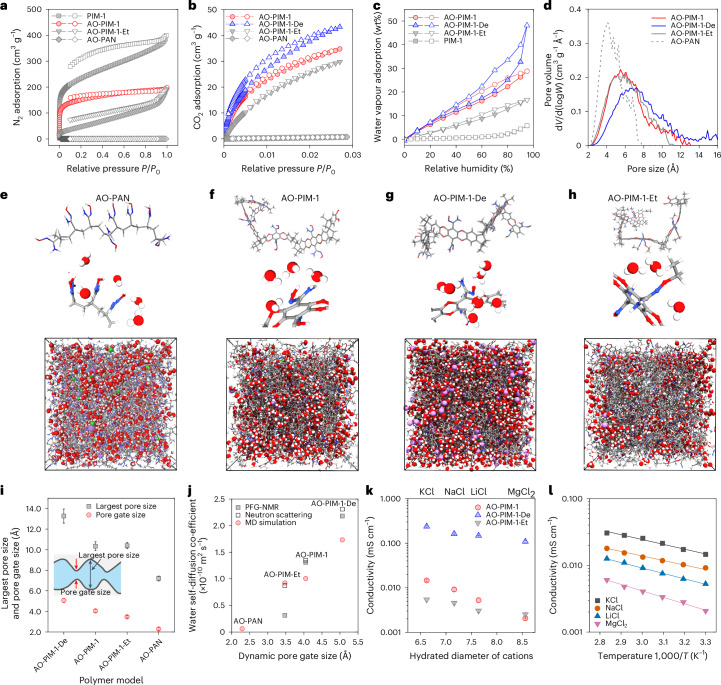
Pore structures and ion conduction. **a**, N_2_ adsorption at 77 K. Four typical samples: PIM-1, AO-PIM-1, AO-PIM-1-Et, AO-PAN. *P*, equilibrium pressure; *P*_0_, saturation pressure. **b**, CO_2_ adsorption at 273 K. Four typical samples: AO-PIM-1, AO-PIM-1-De, AO-PIM-1-Et, AO-PAN. **c**, Water vapour adsorption isotherms as a function of relative humidity at 25 °C (solid symbols: adsorption; open symbols: desorption). AO-PIM-1, AO-PIM-1-De, AO-PIM-1-Et and PIM-1. **d**, Pore size distribution of hydrated polymers derived from computational models, measured by a 1 Å diameter probe. d*V*, pore volume; d(log*W*), pore width. **e**–**h**, Computational models of chain segments, water molecules bound to hydrophilic groups and hydrated polymer models for AO-PAN (**e**), AO-PIM-1 (**f**), AO-PIM-1-De (**g**) and AO-PIM-1-Et (**h**). The size of amorphous cells are: 53.8 Å (AO-PAN), 52.3 Å (AO-PIM-1), 56.5 Å (AO-PIM-1-De) and 53.7 Å (AO-PIM-1-Et). Colour of atoms and ions: red—oxygen; blue—nitrogen; grey—carbon; white—hydrogen; purple—lithium ions. **i**, Largest pore size and dynamic pore gate size, derived from hydrated polymer models. The data are presented as the mean ± s.d. (*n* = 10); the error bars represent the standard deviation (s.d.). **j**, Water self-diffusion coefficients versus the dynamic pore gate size derived from computational models. The water self-diffusion coefficients were probed by PFG-NMR and neutron scattering and calculated by MD simulation, respectively. **k**, Ionic conductivity as a function of hydrated diameter of salt ions, measured experimentally in 0.1 M KCl, 0.1 M NaCl, 0.1 M, LiCl and 0.1 M MgCl_2_ at 30 °C. The data are presented as the mean ± s.d. (*n* = 3); the error bars represent the standard deviation (s.d.). **l**, Temperature dependence of ionic conductivity of pretreated and pristine AO-PIM-1 membranes measured experimentally in 0.1 M NaCl solution. *T*, temperature. Source data

The intrinsic microporosity and water sorption in membranes play a critical role in controlling the formation of water channels and ion transport channels. N_2_ adsorption at 77 K and CO_2_ sorption at 273 K (Fig. 2a,b and Supplementary Fig. 13) confirm that these modified PIM polymers retain their microporosity. In contrast, AO-PAN exhibits negligible gas adsorption in the solid state. Figure 2c illustrates the water vapour sorption in AO-PIM-1, AO-PIM-1-De and AO-PIM-1-Et, with relative humidity up to 95%. Generally, the modification of nitrile groups to hydrophilic amidoxime groups enhances the water sorption (Supplementary Fig. 14). The AO-PIM-1 exhibits a water uptake of about 30 wt% with low swelling ratio (Supplementary Fig. 14). The charged AO-PIM-1-De membrane shows a much higher water uptake (up to 60 wt%). The deprotonation might also weaken the hydrogen bonding between the amidoxime groups, leading to a loose polymer network with a relatively broad pore size distribution. AO-PIM-1-Et shows a relatively low water adsorption (20 wt%), owing to the substitution of hydroxyl groups with hydrophobic ethyl groups.

To understand the pore structures and complicated ion–polymer–water interactions, we built hydrated polymer models based on the water uptakes measured experimentally and performed molecular dynamics simulations (Supplementary Fig. 15). As presented in Fig. 2d–h, the change of backbone and amidoxime functional groups leads to substantial change in the pore size distribution (Fig. 2d), hydration and electrostatic charge (Supplementary Fig. 16). The simulated pore size distribution of AO-PIM-1 in the hydrated state reveals a minimal change in the range of 2–10 Å with peak at around 5 Å (Supplementary Fig. 17). After deprotonation, the polymer becomes negatively charged, and pore size distribution reveals an evident shift towards large pores (peak at 6–8 Å) due to swelling. The derived radial distribution functions (RDFs) validate the narrow separation distance (~3 Å) between the amidoxime groups (Supplementary Fig. 18), suggesting strong inter/intrachain interactions, for example, hydrogen bonding. Modelling also revealed that unconnected and small water clusters are distributed in the AO-PAN (Fig. 2e), while in charge-neutral AO-PIM-1 polymer nanometre-sized water clusters are more connected (Fig. 2f). After deprotonation, the water clusters display an evident aggregation and become fully connected (Fig. 2g), which agrees with the high electrolyte uptake measured experimentally. In contrast, the water clusters in AO-PIM-1-Et are isolated from each other (Fig. 2h), due to the substitution with relatively hydrophobic ethyl groups. Radial number density distribution functions reveal strong signals at separation distances of 2–4 Å between water and amidoxime groups, indicating a certain number of water molecules bound to the functional groups in the form of hydration shells (Supplementary Fig. 19).

The pore structures of hydrated polymer membranes are critical for water and ion transport. The interconnected hydrated micropores in the polymer membranes can be visualized as an hourglass-shaped architecture (Fig. 2i). We hypothesize that the dynamic pore gates in the membranes are the bottlenecks that restrict the motion of water and ions between the micropores, while the diffusion in the micropores is relatively fast. Following our previous approach^40^, we quantified the largest pore sizes and dynamic pore gate sizes by molecular dynamics. The non-porous AO-PAN exhibits narrow gates (2.3 ± 0.1 Å), which are even slightly smaller than the kinetic diameter of water molecules (~2.8 Å), restricting the water mobility. AO-PIM-1 exhibits an average pore gate size of 4 ± 0.17 Å. In contrast, the AO-PIM-1-De shows slightly larger pore gates (5.1 ± 0.2 Å). Figure 2j presents the water self-diffusion coefficients through the hydrated pores calculated by molecular simulation and measured experimentally by pulsed field gradient (PFG) NMR spectroscopy (Supplementary Fig. 20 and Supplementary Table 1) and quasi-elastic neutron scattering (QENS) spectroscopy (Supplementary Fig. 21). For the membranes with relatively large pore gates, the water self-diffusion coefficients are dramatically boosted, as revealed by PFG-NMR spectroscopy. For example, AO-PIM-1 shows a water self-diffusion coefficient of 1.3 × 10^−10^ m^2^ s^−1^, which is one order of magnitude lower than that of bulk water (2.3 × 10^−9^ m^2^ s^−1^ at 25 °C). QENS measurements suggest multimodal diffusion of water in PIM membranes, including localized diffusion (*D*_loc_) and long-range diffusion (*D*_lr_). The long-range diffusion agrees well with that measured by PFG-NMR. The localized diffusion reflects the fast diffusion of water within the confined micropores, as observed previously in Nafion and other membranes^40,47,48^. Detailed analysis of QENS data is provided in Supplementary Information Fig. 21.

The ion conductivities of PIM membranes were derived from through-plane resistances measured by electrochemical impedance spectroscopy (Supplementary Figs. 22–26). As presented in Fig. 2k,l, the conductivity of salt ions decreases with the size of cations, K^+^ (hydrated diameter 6.62 Å), Na^+^ (7.16 Å), Li^+^ (7.64 Å) and Mg^2+^ (8.56 Å) (Supplementary Table 2). We quantified the ion transference number for both cations and anions in the PIM membranes (Supplementary Fig. 27). Both cations and smaller anions (Cl^−^, hydration diameter of 6.4 Å) contribute to the migration under electric field; therefore, the conductivity is considered as the overall apparent conductivity instead of cations alone. However, it remains difficult to fully decouple the transport of anions and cations in these confined nanopores. The activation energies were derived from ion conductivity measurements at varied temperatures (Supplementary Fig. 26), reflecting the average energy barriers for electromigration of both cations and anions within the membrane under electric field. The narrow pore size distribution in the AO-PIM-1 polymers confine salt ions within the subnanometre pores. The low MgCl_2_ conductivity and high activation energy was due to steric hindrance of subnanometre channels, which restrict the migration of large Mg^2+^ ions with large hydration shells.

## Ion separation performance

To gain fundamental understanding of the ion diffusion under the driving force of concentration without the influence of electromigration driven by an electric field, concentration-driven diffusion dialysis performance of PIM membranes was evaluated in H-type cells (Supplementary Fig. 28). For single-component salt ion diffusion, AO-PIM-1 membrane gives decent monovalent/divalent ion selectivity, with K^+^/Mg^2+^ selectivity at 200, Na^+^/Mg^2+^ selectivity at 120 and Li^+^/Mg^2+^ selectivity about 100 (Supplementary Fig. 29). For binary mixtures, the membrane presents much higher monovalent/divalent ion selectivity, with K^+^/Mg^2+^ selectivity at 1,000, Na^+^/Mg^2+^ selectivity at 500 and Li^+^/Mg^2+^ selectivity about 150 (Supplementary Fig. 30). The higher selectivity in binary mixtures suggests the competitive ion transport between smaller and large cations through the AO-PIM-1 membranes, which is mainly due to the decreased Mg^2+^ permeation rate resulted from weaker partitioning compared with Li^+^. A similar phenomenon has been observed in other nanoporous membranes, such as covalent organic frameworks^26^. In contrast, the AO-PIM-1-De membranes with deprotonation of hydroxyl groups display poor selectivity (K^+^/Mg^2+^ about 7) (Supplementary Fig. 31), which is due to the swollen pores with a relatively broader pore size distribution compared to the unmodified pristine AO-PIM-1 membrane.

**Fig. 3:**
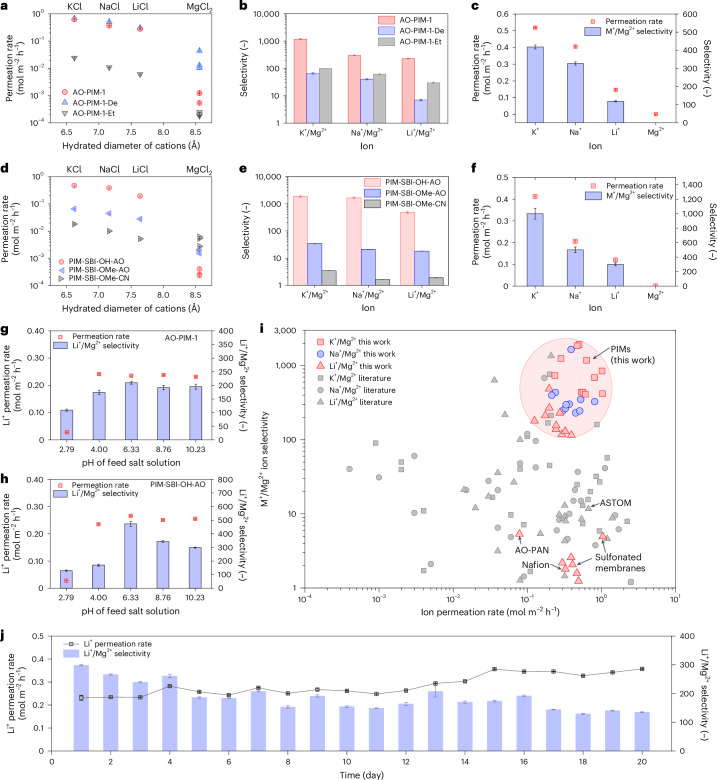
Ion separation performance via selective electrodialysis. **a**, Ion permeation rates of binary salt mixtures for AO-PIM-1, AO-PIM-1-De and AO-PIM-1-Et membranes. **b**, Monovalent/divalent ion selectivity of AO-PIM-1, AO-PIM-1-De and AO-PIM-1-Et membranes. **c**, Ion permeation rate and selectivity of AO-PIM-1 membrane for feed solution of KCl/NaCl/LiCl/MgCl_2_ mixtures (0.1 M each). **d**, Ion permeation rates of binary salt mixtures for PIM-SBI-OH-AO, PIM-SBI-OMe-AO and PIM-SBI-OMe-CN. **e**, Monovalent/divalent ion selectivity of PIM-SBI-OH-AO, PIM-SBI-OMe-AO and PIM-SBI-OMe-CN membranes. **f**, Ion permeation rate and selectivity of PIM-SBI-OH-AO membrane for feed solution of KCl/NaCl/LiCl/MgCl_2_ mixtures (0.1 M each). In **a**–**f**, the error bars of permeation rate data represent the standard errors derived from linear fittings of salt concentration profiles of three independent experiments, and the error bars of selectivity data represent uncertainties derived from the permeation rates. **g**, Ion permeation rate and selectivity of AO-PIM-1 membrane in Li/Mg binary salt mixtures at varied pH. **h**, Ion permeation rate and selectivity of PIM-SBI-OH-AO membrane in Li/Mg binary salt mixtures at varied pH. **i**, Plot of monovalent/divalent ion selectivity versus monovalent ion permeation rates. Literature data are included for comparison (shown in Supplementary Table 4). Typical commercial ion-exchange membranes are included for comparison: Nafion (tested in this work) and ASTOM commercial monovalent selective membranes^26^. **j**, Long-duration performance of electrodialysis tests of AO-PIM-1 membranes for 20 days. In **g**, **h** and **j**, the error bars of permeation rate data represent the standard errors derived from linear fittings of salt concentration profiles, and the error bars of selectivity data represent uncertainties derived from the permeation rates. Source data

The electrodialysis performance of PIM membranes was evaluated in laboratory-scale electrodialysis cells with an effective area of 2 cm^2^ (Supplementary Fig. 32 and Supplementary Table 3). Firstly, AO-PIM-1 membrane was tested with LiCl/MgCl_2_, KCl/MgCl_2_ and NaCl/MgCl_2_ binary mixtures under different current densities (1–3 mA cm^−2^) (Supplementary Fig. 33). Results indicate that under 2 mA cm^−2^, AO-PIM-1 could achieve optimal ion selectivity. Under the driving force of the electric field, the ion separation performance was less sensitive to the change of membrane thickness (Supplementary Fig. 34). Therefore, in most experiments, membranes with thickness around 50 µm were tested. As shown in Fig. 3a, pristine AO-PIM-1 membranes display a size-sieving phenomenon, with fast diffusion of smaller monovalent alkali metal cations (K^+^, Na^+^, Li^+^) with permeation rates of 0.2–1.0 mol m^−2^ h^−1^ while rejecting larger divalent cations (Mg^2+^) with slower permeation rates around 10^−3^ mol m^−2^ h^−1^. AO-PIM-1 membrane exhibits excellent monovalent/divalent ion selectivity, with K^+^/Mg^2+^ selectivity at 1,180, Na^+^/Mg^2+^ selectivity at 300 and Li^+^/Mg^2+^ selectivity about 230 (Fig. 3b).

We further confirmed the selective ion separation performance using a salt mixture of KCl/NaCl/LiCl/MgCl_2_ (Fig. 3c and Supplementary Fig. 35). The monovalent/divalent ion selectivity is relatively lower than that of binary mixtures, due to the competitive transport between small monovalent cations through the membranes. The combined monovalent/divalent ion selectivity is still as high as 1,000. The performance of AO-PIM-1 in binary mixtures with varied Mg^2+^/Li^+^ mass ratios (10, 20, 40, 60 and 80) were also investigated (Supplementary Fig. 36). Selective separation of Li^+^ and Mg^2+^ were achieved under all Mg^2+^/Li^+^ mass ratios. When the mass ratio of Mg/Li was 20, the Li/Mg selectivity was the highest (around 240). The AO-PIM-1 membrane also maintained reasonably high selectivity (>100) with the electrolyte concentration increased from 0.05 M to 1 M (Supplementary Fig. 37).

The structural and chemical properties of polymer membranes influence the ion transport characteristics and selectivity. The PIM polymer backbone provides the scaffold on which functional groups are attached, which determines the microporosity and density of functional groups in the pore walls and thus influences how the ions interact with polymers. The non-porous AO-PAN membrane showed much lower ion permeation rates (Li^+^ ~0.078 mol m^−2^ h^−1^) and relatively low monovalent/divalent selectivity (Li^+^/Mg^2+^ ~5.3) (Supplementary Fig. 38). The slow ion transport through these non-porous membranes is due to the less-connected water channels in the polymer matrix. We also evaluated the AO-PIM membranes with more rigid backbones, such as AO-PIM-SBF and AO-PIM-DMBP. According to our previous work^36^, AO-PIM-1, AO-PIM-SBF and AO-PIM-DBMP membranes have similar gas sorption capacities, pore size distributions in the dry state and similar water uptake. Therefore, these membranes demonstrated similar ion permeation rates and comparable mono/divalent ion selectivity under electrodialysis conditions (Supplementary Fig. 39).

To understand the effect of amidoxime functional groups on the ion transport, we also tested AO-PIM-1-De and AO-PIM-1-Et at the same conditions as AO-PIM-1 membranes (Fig. 3a,b and Supplementary Figs. 40–42). The deprotonated AO-PIM-1-De membrane allows fast permeation of monovalent ions and divalent Mg^2+^ ions, resulting in much lower selectivity, for example, Li/Mg < 10. The moderate selectivity of AO-PIM-1-De can be due to the combination of several features and physical principles that influence the transport of monovalent and divalent ions. One key feature is the swelling of the deprotonated AO-PIM-1-De membrane due to excessive electrolyte uptake, which behaves like ion-exchange membranes to some extent. The swelling led to a broader pore size distribution, which weakens the size-sieving selectivity, allowing the diffusion of relatively large Mg^2+^ ions. The negatively charged amidoxime groups could also facilitate the electromigration of positively charged Mg^2+^ under electric field due to strong interactions between divalent Mg^2+^ ions and deionized amidoxime groups.

We also substituted the hydroxyl groups in the AO-PIM-1 with non-polar hydrophobic ethyl groups (AO-PIM-1-Et) while keeping the hydrophilic amine groups (Supplementary Figs. 41 and 42). Compared to AO-PIM-1, AO-PIM-1-Et gives almost two orders of magnitude lower monovalent ion permeation rate (Li^+^ ~0.00612 mol m^−2^ h^−1^) and restricted divalent Mg^2+^ transport, with Li^+^/Mg^2+^ selectivity at around 30. According to gas adsorption measurements and MD simulation, the micropore volume was largely maintained in AO-PIM-1-Et, the incorporation of hydrophobic ethyl groups reduces water adsorption to some extent and formed less-connected water channels, resulting in slightly narrow dynamic pore gate size (~3.5 Å) and slightly lower water diffusion coefficient (Fig. 2j). The evident drop in the ion permeation rate could be mainly attributed to the removal of hydroxyl groups, which play a crucial role in enhancing the interactions with alkali metal ions.

Another group of PIM polymers were prepared by superacid-catalysed Friedel–Crafts reactions, including PIM-SBI-OH-CN and PIM-SBI-OMe-CN and modified polymers with amidoxime groups (PIM-SBI-OH-AO and PIM-SBI-OMe-AO) (Supplementary Figs. 43–48). These polymers with similar backbone and microporosity but varied amidoxime and hydroxyl groups serve as control samples to study the pore environment and ion–pore interactions. PIM-SBI-OH-AO polymer demonstrated high monovalent ion permeation rates in electrodialysis (Fig. 3d) and remarkable monovalent/divalent ion selectivity in binary mixtures, for example, K^+^/Mg^2+^ selectivity at 1,850, Na^+^/Mg^2+^ selectivity at 1,650 and Li^+^/Mg^2+^ selectivity at about 485 (Fig. 3e). The membrane maintained high ion selectivity in KCl/NaCl/LiCl/MgCl_2_ salt mixtures (Fig. 3f and Supplementary Fig. 43) and high feed concentrations (Supplementary Fig. 44). The control sample PIM-SBI-OMe-AO with methyl group substitution exhibits much lower ion permeation rates (Fig. 3d) and poor selectivity (Fig. 3e and Supplementary Figs. 45 and 47). In contrast, PIM-SBI-OMe-CN shows negligible selectivity and slow ion transport which is two orders of magnitude lower due to the hydrophobic pore environment and lack of favourable functional groups (Fig. 3d,e and Supplementary Figs. 46 and 47). A control sample with nominal 50% hydroxyl groups and 50% methyl groups (PIM-SBI-OMe_0.5_-OH_0.5_-AO) validated the critical role of hydroxyl groups (Supplementary Fig. 48). These membranes display similar CO_2_ adsorption capacity and similar pore size distribution in the solid state. With the increasing ratio of hydroxyl groups, the membrane pores became more hydrophilic with both ion permeation rate and ion selectivity enhanced. These results suggest the hydroxyl groups not only enhance the local hydrophilicity to improve the connectivity of water channels but also facilitate the interactions with salt ions and their transport.

To further investigate the role of amidoxime groups and microporosity, we prepared blend membranes using PAN and PIM-SBI-OMe-CN and then performed amidoxime modification to produce membranes with AO functional groups (Supplementary Fig. 49). Compared to the individual AO-PAN and PIM-SBI-OMe-AO membrane, the blend membrane combines the features of microporosity and high loading of amidoxime groups, as confirmed by gas adsorption and FTIR spectra. The resulting blend membrane provided higher permeation rates and high mono/divalent ion selectivity (Li^+^/Mg^2+^ ~60), which can be attributed to the synergy of enhanced microporosity and high-loading amidoxime groups within the micropores.

Besides AO-PIM membranes, we also developed PIM polymers with other functional groups to verify the effect of ion–membrane interactions on ion transport. First, we prepared PIM membranes with carboxylic acid groups (cPIM-1, cPIM-Et, cPIM-Ph) (Supplementary Figs. 50 and 51), ion-exchanged carboxylate (cPIM-Et-De and cPIM-Ph-De) (Supplementary Fig. 52) and a control sample with carboxylic acid groups substituted with methyl groups (cPIM-1-OMe) (Supplementary Fig. 50). It should be noted that although the cPIM-1 membrane had high ion selectivity, it broke after about 20 min in the electrodialysis cell due to excessive hydration and swelling after complete ion exchange^38^. To solve the swelling problem of cPIM-1, we developed carboxylic acid-functionalized PIMs with pendant groups of tailored hydrophobicity, which have limited degree of swelling and hence are more stable in salt solution. These two membranes demonstrate high selectivity and stability for effectively separating monovalent ions from divalent ions (Supplementary Fig. 51). Compared to AO-PIM-1, cPIM-Et gives slightly higher ion permeation rates and lower ion selectivity, whereas cPIM-Ph shows relatively lower ion permeation rate and higher selectivity. The polymer membranes were treated with strong base (1 M NaOH) to fully deprotonate the carboxylic acid to form carboxylate groups (termed as cPIM-Et-De and cPIM-Ph-De). Similar to deprotonated AO-PIM-1-De, the deprotonated cPIM-Et-De and cPIM-Ph-De membranes lose their selectivity in electrodialysis (Supplementary Fig. 52). Furthermore, cPIM-1-OMe, a control sample substituted with hydrophobic non-polar methyl end group, shows negligible selectivity and poor ion transport. These control experiments confirm the important role of hydrophilic groups as ion binding sites for the selective ion transport.

Polymer membranes with easily dissociated sulfonate groups were also tested, and they generally show negligible selectivity (<5). Normal sPEEK membrane, sPEEK-Trip membranes, sPIM-1-ES and sPIM-Ph-ES with sulfonate groups were verified to have relatively high ion permeation rate but negligible selectivity. Such poor ion selectivity is comparable to that of conventional cation exchange membranes such as Nafion (Li^+^/Mg^2+^ selectivity ~1.8) (Supplementary Fig. 53). These polymers with sulfonic acid (-SO_3_H) groups can easily dissociate and become negatively charged in salt solution. Despite that PIM backbones are applied in some of these polymers, the fully ion-exchanged membranes suffer from swelling, leading to enlargement of dynamic pore gates, and consequently poor selectivity. These strong electrostatic interactions between negatively charged sulfonated membranes and Mg^2+^ ions tend to favour the electromigration of Mg^2+^ over monovalent ions through the membrane under the electric field.

The above control experiments demonstrated the importance of regulating the electrostatic charges of the membranes and their interactions with ions, which can dramatically influence the ion separation performance. The pH of salt-lake brine solutions could also influence the electrostatic charges of functional groups and hence affect the ion selectivity. It is well known that the amidoxime groups could undergo protonation at low pH and deprotonation at high pH, which would have important impact on the electrostatic charges of membrane surfaces. Figure 3g,h and Supplementary Fig. 54 present the ion permeation rates and selectivity of AO-PIM-1 and PIM-SBI-OH-AO membranes at varied pH of feed solutions. Generally, the membranes provide high ion permeation rates and high selectivity over a broad pH range (4–10). At relatively higher pH (>7), the ion selectivity decreases slightly which might be associated with the partial ionization of the –OH groups in the amidoxime functional groups in AO-PIM-1 or in PIM-SBI-OH-AO polymer. When the pH decreases to 2.79, the Li^+^ ion permeation rate drops by ten times. At low pH (<4), the amidoxime groups become positively charged due to protonation, which lead to electrostatic repulsion towards positively charged alkali metal cations (Li^+^ and Mg^2+^) and consequently lower permeation rates, while maintaining high selectivity (>100). Overall, the AO-PIM membranes maintain high selectivity over a broad pH range, which makes them suitable for processing of salt-lake brines (the pH is typically within 7–11).

Figure 3i shows the plot of monovalent/divalent ion selectivity versus the ion permeation rate for PIM membranes and the comparison with membranes reported in the literature, including ion-exchange membranes and nanofiltration membranes (detailed data are presented in Supplementary Table 4). Generally, PIM membranes with hydrophilic functional groups present evidently high monovalent/divalent ion selectivity, which are much higher than conventional polymeric membranes.

We also evaluated the operation stability of AO-PIM-1 membranes in the electrodialysis cell (Fig. 3j). The membrane demonstrated a slowly increasing Li ion permeation rate from 0.23 to 0.35 mol m^−2^ h^−1^ over a continuous operation for 20 days (about 480 h), whereas the Li/Mg selectivity decreased from ~250 to 150. The slow increase of ion permeation rate and slight decay in selectivity may be due to the swelling of membranes over time and slow penetration of Mg^2+^ ions through the membranes. Nevertheless, the ion selectivity is still significantly higher than traditional polymer ion-exchange membranes. Characterizations of the membrane recovered from the long-duration tests suggested that the membranes were stable, as verified by NMR, FTIR, tensile strength tests and SEM imaging analysis (Supplementary Fig. 55).

## Non-equilibrium molecular dynamics simulations

**Fig. 4:**
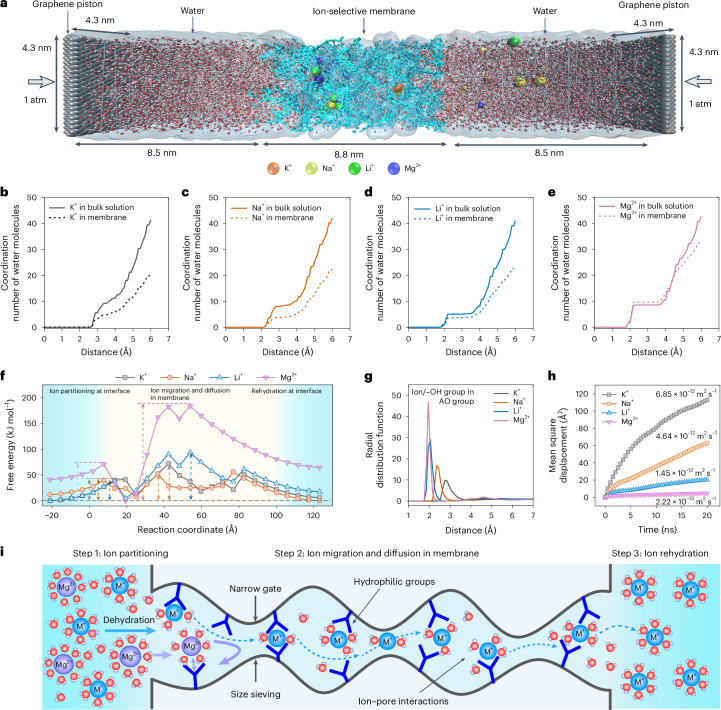
Molecular dynamics simulations of ion transport in AO-PIM-1 membrane. **a**, Set-up of non-equilibrium model for ion transport through one AO-PIM-1 membrane. The AO-PIM-1 membrane with thickness of 8.8 nm is sandwiched between two water chambers, with graphene sheet pistons at two ends. Standard atmospheric pressures are applied on both graphene sheets. The ion transport is driven by an electric field. **b**–**e**, Coordination number of water molecules as a function of the distance from the centre of ions: K^+^ (**b**), Na^+^ (**c**), Li^+^ (**d**), Mg^2+^ (**e**). **f**, Transport energy barrier profiles of ion transport through the membrane. Light blue colour: interface region at membrane surface. Light yellow colour: membrane region. Short dashed arrows indicate the energy barriers for ion partitioning and ion migration and diffusion in membrane, and dashed lines correspond to the levels of free energy of salts. **g**, Radial distribution function of ions bound to the hydroxyl groups on the AO groups, as a function of the distance between ions with hydroxyl groups in amidoxime groups. **h**, Modelled mean squared displacement of cations in AO-PIM-1 membrane as a function of time. Cation self-diffusion coefficients were included. **i**, Schematic diagram showing the mechanism of ion separation through PIM membranes with interconnected subnanometre channels. Source data

To gain a deep understanding of the ion separation mechanism, we performed non-equilibrium molecular dynamics simulations to study the ion transport under an electric field of 0.03 V Å^−1^ (Fig. 4a and Supplementary Fig. 56). Generally, the overall ion transport mechanism through membranes is a combination of the ion partitioning via dehydration process at the pore entrance and the confined electromigration and diffusion of partially dehydrated ions within the subnanometre pores. The hydration of ions can be quantified by the coordination number of water molecules (Fig. 4b–e), which is derived from the RDFs (Supplementary Fig. 57). The simulations suggest the coordination number of water molecules decreases when the ions move from the bulk solution into the membranes, indicating that partial dehydration of K^+^, Na^+^ and Li^+^ ions occur. Highly charged ions such as Mg^2+^ have higher hydration numbers due to their strong electrostatic fields that attract more water molecules, which agree well with the high hydration energy (Supplementary Table 2).

The energy barrier of ion transport through the PIM membrane was calculated to investigate the energy transition during the ion transport process (Fig. 4f and Supplementary Fig. 58). Two steps of energy change associated with ion transport could be observed, including (1) ion partitioning step and (2) migration and diffusion in the membrane. Following literature work^14^, we could estimate the energy barriers for both ion partitioning and migration/diffusion (Supplementary Table 5 and Supplementary Fig. 59). The energy barriers for ion partition are relatively low due to the large pore entrance, which only requires partial ion dehydration. In contrast, the energy barriers for ion migration/diffusion are relatively high and governing the overall energy barrier for ion transport, for example, K^+^ (62 kJ mol^−1^), Na^+^ (49 kJ mol^−1^), Li^+^ (90 kJ mol^−1^) and Mg^2+^ (183 kJ mol^−1^). The high energy barrier could be attributed to the energy required for dehydration through the narrow pore gates and ion binding with the functional groups in the pore walls. The interactions of cations with the functional groups at the pore entrance and pore walls are also important for the ion transport. RDFs of polymer models in the presence of salt ions also suggest strong binding between salt ions and the hydroxyl groups in amidoxime groups (Fig. 4g and Supplementary Fig. 60). Particularly the short distance between Mg^2+^ ions and AO groups suggests that the hydrophilic groups may be bound to the solvation shell of partially dehydrated Mg^2+^ ions when they are confined in the subnanometre channels. The binding energies of K^+^, Na^+^, Li^+^ and Mg^2+^ with the amidoxime groups were calculated from the DFT simulations, following the order of K^+^ < Na^+^ < Li^+^ < Mg^2+^ (Supplementary Table 5). For divalent ions to move between functional groups, this strong interaction needs to be overcome, leading to a large energy barrier for their diffusion. The hydrophilic groups can form hydrogen bonds with water molecules, enhancing the hydration level of the pore environment. Monovalent ions have smaller hydration shells compared to divalent ions, so they exhibit greater mobility because of their weaker interactions with water molecules. Figure 4h shows the mean squared displacement of different ions through the membranes, indicating the preferential transport of smaller cations (K^+^, Na^+^, Li^+^) over divalent Mg^2+^ ions through the confined membrane water channels. The cation diffusion coefficient correlates well with the hydrated diameter of cations, suggesting the size sieving effect due to the narrow subnanometre channels, especially the narrow dynamic pore gates.

The ion transport through the membranes can be divided into three steps (Fig. 4i), including (1) ion partitioning into the narrow pore channels, (2) ion migration and diffusion through the interconnected micropores and (3) ion rehydration. Combining the modelling and experimental results, we can conclude that the superior monovalent–divalent ion selectivity of neutral-charge PIM membranes could be attributed to the synergistic effects of (1) regulated dehydration and ion partitioning into narrow subnanometre-sized ion channels, (2) confined transport of partially dehydrated ions through the subnanometre pores, especially size sieving by the narrow pore gates with appropriate sizes and (3) favourable interactions between monovalent ions with a sufficient amount of hydrophilic functional groups (for example, amidoxime, carboxylic acid, hydroxyl) in the hydrated pores.

## Separation performance in upscaled electrodialysis stack

**Fig. 5:**
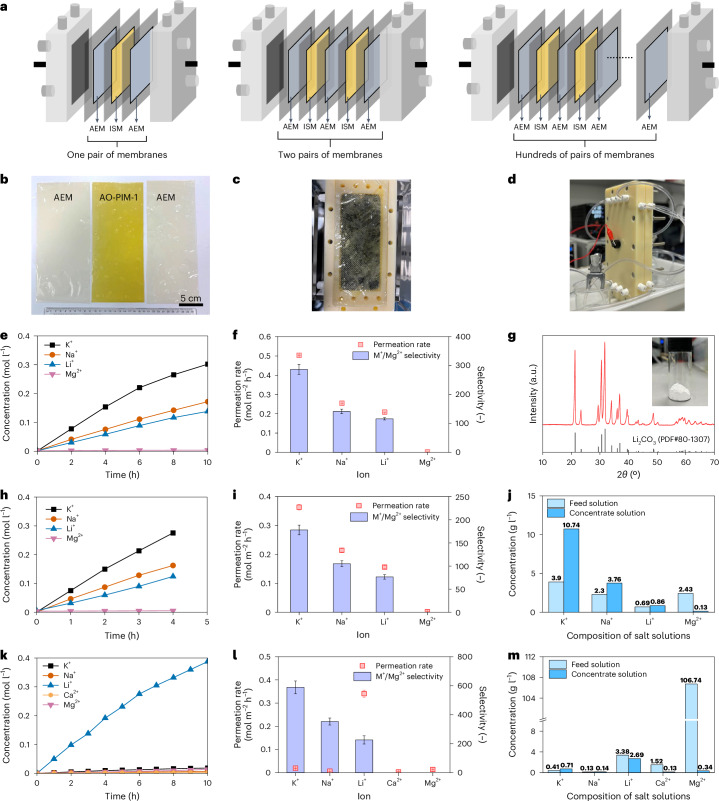
Lithium extraction in a laboratory-scale electrodialysis stack. **a**, Schematic diagrams of electrodialysis stacks. ISM, ion-selective membrane; AEM, anion exchange membrane. **b**, Photos of one pair of membranes, including one piece of AO-PIM-1 membrane (ISM) and two pieces of AEM membranes. **c**, Photo of the membrane assembled in the electrodialysis stack. **d**, Photo of one electrodialysis stack in operation. **e**,**f**, Ion concentration profiles of concentrate chamber for electrodialysis separation with one pair of membranes (**e**), with feed solution of KCl/NaCl/LiCl/MgCl_2_ (0.1 M) and derived ion permeation rates and selectivity (**f**). **g**, X-ray diffraction pattern of Li_2_CO_3_ product. The inset photo shows the purified Li_2_CO_3_ solid. **h**,**i**, Ion concentration profiles of concentrate chamber for electrodialysis separation with two pairs of membranes (**h**), with feed solution of KCl/NaCl/LiCl/MgCl_2_ (0.1 M) and derived ion permeation rates and selectivity (**i**). **j**, Compositions of initial feed solution and concentrate solution after testing for 4 h. **k**, Ion concentration profiles of concentrate chamber, with simulated salt-lake brine solution (after removal of Na^+^ and K^+^) as feed solution. **l**, Ion permeation rates and selectivity derived from **k**. **m**, Compositions of initial feed solution and concentrate solution after testing for 10 h. The *y* axis includes a break from 10 to 100. In **f**, **i** and **l**, the error bars of permeation rate data represent the standard errors derived from linear fittings of salt concentration profiles, and the error bars of selectivity data represent uncertainties derived from the permeation rates. Source data

Testing of membranes in an electrodialysis stack is a critical step before scaling them up in an industrial scale pilot system (Fig. 5a). To demonstrate the upscaling potential of PIM membranes for lithium extraction, we integrated our newly developed membranes into electrodialysis stacks with relatively large effective area of 189 cm^2^. We prepared one pair of membranes (one piece of AO-PIM-1 and two pieces of anion-exchange membranes (AEMs)) (Fig. 5b) and assembled the membranes in a lab-scale electrodialysis stack with an effective area of 189 cm^2^. These membranes were assembled into one electrodialysis stack (Fig. 5c,d and Supplementary Fig. 61). The performance of AO-PIM-1 membrane was evaluated with KCl/NaCl/LiCl/MgCl_2_ mixed solution with initial concentration of 0.1 M for each salt ion. The salt concentration profiles of concentrate stream are illustrated in Fig. 5e. Figure 5f shows the permeation rate and selectivity of AO-PIM-1 for ion separation using the large-area stack. The results in the stack still demonstrated preferential transport of K^+^, Na^+^ and Li^+^ with high selectivity towards Mg^2+^ (over 100). The final product solution was evaporated to enhance Li^+^ concentration to over 3 mol l^−1^. Lithium can be precipitated easily using Na_2_CO_3_, resulting in the production of high purity battery-grade Li_2_CO_3_ with purity up to 99.6 wt% based on inductively coupled plasma mass spectrometry (ICP-MS) measurement. The X-ray diffraction pattern of precipitated Li_2_CO_3_ product is presented in Fig. 5g.

To demonstrate this upscaling effect in the electrodialysis stack, we performed experiments using electrodialysis stack with two pairs of membranes (two pieces of AO-PIM-1 and three pieces of AEMs) (Fig. 5h–j). Owing to the enlarged membrane areas, the processing efficiency of the stack is almost two times that with one pair of membranes (Fig. 5h). Therefore, for salt solution with 0.1 M for each ion, the specific energy consumption for Li extraction with two pairs of membranes (10.94 kWh kg^−1^) is only half of that for electrodialysis stack with one pair of membranes (19.87 kWh kg^−1^). Similar improvement in processing efficiency and reduction in energy consumption was observed with simulated brine solutions (Supplementary Figs. 62 and 63). The slightly lower ion selectivity (Fig. 5i) was due to the relatively low perm-selectivity of the AEM membrane, leading to leakage of co-ions (cations). This can be improved by using AEM membranes with high perm-selectivity in the future. The mass ratio of Mg/Li in the concentrate solution was largely reduced yet the concentrations of K^+^ and Na^+^ ions were enriched (Fig. 5j) due to their preferential transport over lithium ions.

We further conducted experiments to test the performance of AO-PIM-1 membranes for separation of salt mixtures with compositions similar to hypersaline brine extracted from salt-lake reservoirs in China (Supplementary Table 6). Li^+^ transport was relatively slow due to the low initial concentration and competition transport, yet the Li/Mg selectivity is still as high as 30 despite the initial high Mg^2+^/Li^+^ mass ratio (~65) (Supplementary Fig. 62). The overall monovalent/divalent ion selectivity is still high. These mixed salt tests prove that AO-PIM-1 still provides good selectivity for lithium extraction with Mg^2+^/Li^+^ ratio reduced evidently. The ionic composition of product solution after 8 h of testing is shown in Supplementary Table 6, indicating that the mass ratio of Mg/Li could be reduced from 65 to around 2.

The preferential transport of sodium and potassium over lithium could be a problem for processing of real brine solution with high sodium and potassium concentration. Membranes with monovalent ion selectivity (for example Li^+^/Na^+^) can be developed by integrating ceramic ion-conductor^49^ or special lithium-binding functional groups^50^. In industrial processes of lithium extraction from brines, brine solutions are usually processed via multiple steps^51^. Electrodialysis could be used to process the evaporated brine solutions or real brine solution with relatively low sodium concentration. Hence, we evaluated the electrodialysis separation performance for processing of brine solutions with K^+^ and Na^+^ removed (Fig. 5k and Supplementary Table 7) and obtained high monovalent/divalent selectivity (Fig. 5l) and lower Mg/Li mass ratio in the concentrate solution (Fig. 5m). More comprehensive studies should be performed with a variety of hypersaline salt-lake brines and long-term system operation in realistic operation conditions^10^.

Membranes used in electrodialysis processes are exposed to dynamic changing chemical environment, including exposure to various ions, pH fluctuations and potential oxidative or reductive conditions. To demonstrate the stability and recyclability of PIM membranes in selective electrodialysis, we recycled the membranes and evaluated their performance in the electrodialysis stack. The recovered AO-PIM-1 membranes maintain the same structures as fresh membranes, as characterized by NMR and FTIR spectroscopy (Supplementary Fig. 64), confirming their excellent chemical stability. The dried membranes were redissolved in dimethylformamide and cast into a defect-free membrane (Supplementary Fig. 65). The membrane was evaluated again in the 189 cm^2^ electrodialysis stack and maintained high ion permeation rates and high selectivity (Supplementary Fig. 65).

## Discussion

In summary, we demonstrate microporous polymer membranes with high monovalent–divalent ion selectivity in selective electrodialysis processes for efficient and sustainable lithium extraction. Experimental studies and computational modelling suggest that the superior monovalent–divalent ion selectivity of PIM membranes could be attributed to the synergistic effect of ion partitioning and size sieving regulated by narrow subnanometre-sized ion channels and favourable interactions between ions with functional groups in the hydrated micropores. In particular, the hydrophilic functional groups (for example, amidoxime, carboxylic acid, hydroxyl groups) are less prone to dissociate, and they interact preferentially with alkali cations and enable selective ion transport in hydrated micropores. The ion-selective membranes demonstrate excellent monovalent/divalent ion selectivity in both binary salt mixtures and simulated salt-lake brines, producing high purity battery-grade Li_2_CO_3_. Furthermore, owing to their unique solution processability, the manufacturing of PIM polymer membranes can be potentially scaled up using industrial continuous roll-to-roll membrane production lines and integrated into large membrane stacks. Future work can be focused on development of Li-selective membranes with high ion selectivity towards other monovalent cations. The molecular design of high-selectivity ion separation membranes and their selective electrodialysis performance demonstrated in this work have broad implications for the development of next-generation membrane technologies for industrial separation applications, such as water purification, resource recovery and recycling process, which will contribute to a global circular economy.

## Methods

### Synthesis of polymers

PIM-1 was synthesized using a protocol adapted from the literature^52^, by polymerization of purified monomers including 5,5′,6,6′-Tetrahyroxy-3,3,3′,3′-tetramethyl-1,1′-spirobisindane (TTSBI) and 2,3,5,6- tetrafluoroterephthalonitrile (TFTPN) in anhydrous DMAc and K_2_CO_3_ fine powder at 150 °C. The AO-PIM-1 polymer were synthesized following the protocol reported in the literature^41^. The AO-PIM-1-Et was synthesized following a previous study^53^. Another group of PIM membranes were prepared by superacid-catalysed Friedel–Crafts reactions from 6,6′-dimethoxy-3,3,3′,3′-tetramethyl-2,2′,3,3′-tetrahydro-1,1′-spiro(SBI-OMe), 3,3,3′,3′-Tetramethyl-1,1′-spirobisindane-6,6′-diol (SBI-OH) and 4-formylbenzonitrile in dichloromethane and methanesulfonic acid, forming PIM-SBI-OMe-CN, PIM-SBI-OH-CN and PIM-SBI-OMe_0.5_-OH_0.5_-CN. The nitrile groups of these polymers were modified into amidoxime groups by reacting with hydroxylamine solution in DMSO at 100 °C, forming polymers PIM-SBI-OMe-AO, PIM-SBI-OH-AO and PIM-SBI-OMe_0.5_-OH_0.5_-AO.

AO-PAN membranes were made through amidoximation modification of PAN membranes. PAN polymer was dissolved into DMSO solvent and casted into membrane. Then PAN membranes were modified into AO-PAN membranes by reacting with aqueous hydroxylamine solution (5 g l^−1^) at 60 °C for 3 h (ref. ^54^). AO-PAN/PIM-SBI-OMe-AO blend membrane was prepared by AO modification of blended PAN/PIM-SBI-OMe-CN membranes. PAN and PIM-SBI-OMe-CN polymers were blended with 1:1 ratio and casted to membrane. The membrane was immersed in 50 ml of an aqueous hydroxylamine solution with concentration of 5 g l^−1^ under N_2_ at 60°C for 3 h. After the reaction, the membranes were thoroughly washed multiple times with deionized water.

PIM polymers with carboxylic acid groups were synthesized following the protocol reported in the literature. cPIM-1 was prepared by acid hydrolysis of PIM-1^43^. cPIM-Et and cPIM-Ph were prepared by modification of AO-PIM-1 with succinic anhydride and phthalic anhydride to form pendant groups with ethyl-, phenyl- containing linkages, respectively^40^. The cPIM-Et and cPIM-Ph were further exposed to 1 M NaOH to fully exchange the carboxylic acid to carboxylate, cPIM-Et-De and cPIM-Ph-De. cPIM-1-OMe was prepared by modification of cPIM-1 with Me-I to substitute the hydroxyl groups with methyl groups.

sPEEK polymer was prepared by sulfonation of PEEK. PEEK-Trip was synthesized with triptycene backbone and further sulfonated to form sPEEK-Trip^45^. PIM polymers with sulfonate groups were prepared by esterification of cPIM polymers, including modification of cPIM-1 with 1,3-propanesultone to form sPIM-1-ES, and modification of cPIM-Ph with 1,3-propanesultone to form sPIM-Ph-ES.

Most polymers were fabricated into dense membranes by the solution casting method. More details are available in Supplementary Information.

### Materials characterization

Fourier transform infrared spectroscopy (FTIR) was performed on a Perkin-Elmer Spectrum 100 FTIR spectrometer with polymer membrane samples mounted on a zinc–selenium/diamond plate. Liquid state ^1^H and ^13^C nuclear magnetic resonance (NMR) spectra were collected using Bruker Avance III HD 600 MHz or Jeol 400 MHz spectrometers at 25 °C and 60 °C for samples in CDCl_3_ and *d*-DMSO, respectively. Low-pressure gas physisorption was analysed by Micromeritics 3Flex surface characterization analyser. The N_2_ and CO_2_ adsorption/desorption isotherms were measured at 77 K and 273 K, respectively. Samples were accurately weighed and transferred to measuring tubes and degassed overnight at 100 °C. Afterward, another in situ degas process (80 °C for 2 h) was introduced before the measurement took place. Pore size distribution is derived from both N_2_ and CO_2_ sorption using density functional theory (DFT) calculation. Dynamic vapour sorption (DVS) of water was performed using a gravimetric DVS sorption analyser (Surface Measurement Systems) at 25 °C. Cross-sectional morphology of membranes was characterized by Scanning electron microscopy (SEM) using a Karl Zeiss LEO 1525 microscope. Before testing, the membranes were manually fractured in liquid nitrogen and coated with a thin layer of chromium. The electrolyte uptake or water uptake of the membrane is defined as the weight difference between the wet membrane after soaking in corresponding salt solution or DI water and the dry membrane. The swelling ratio of membranes is defined as the length difference between the wet membrane and the dry membrane. The ion conductivity is derived from two-electrode through-plane membrane resistance measured by the electrochemical impedance spectroscopy technique utilizing a potentiostat, employing an AC bias of 10 mV and scanning frequencies ranging from 0.2 MHz to 10 Hz. Membranes saturated with an aqueous electrolyte were positioned between two stainless steel electrodes, each featuring an effective area of 2 cm^−2^, and securely enclosed within coin cell for ionic conductivity evaluations under elevated temperatures.

### Electrodialysis separation performance tests

This study uses two kinds of lab-scale electrodialysis stack supplied by Hefei Chemjoy Polymer Material Co. For small electrodialysis stack, the effective area of each membrane is 2 cm^2^. Channels with thickness of 1 cm separate two neighbouring membranes. For large electrodialysis stack, the effective area of each membrane is 189 cm^2^. The channel spacers with thickness of 1 mm separate two neighbouring membranes. Typically, the cell is equipped with one piece of ion-selective membranes and two pieces of anion-exchange membranes (PiperION, Fuel Cell Store) placed in parallel. The electrodialysis stack was operated in a galvanostatic mode controlled by a direct current supply.

In the electrodialysis experiments, 100 ml mixed ion solution and 100 ml 0.01 M salt (KCl or NaCl) solution were pumped into dilute and concentrate chambers separately, with a volumetric flow rate of 40 ml min^−1^. The 200 ml Na_2_SO_4_ solution was pumped into the electrode chamber, with volumetric flow rate of 80 ml min^−1^. The electrodialysis experiments were performed in a batch mode in which the effluent streams were circulated back to the respective tanks. For small electrodialysis stack with membrane effective area of 2 cm^2^, the experiments of AO-PIM-1 membrane took 1 or 2 h in total with current density 1 or 1.5 or 2 or 2.5 or 3 mA cm^−2^. Two mA cm^−2^ was selected as a suitable current density, and all the other experiments were performed under this current density with the same operating conditions. For large electrodialysis stack, the mixed ion (K^+^, Na^+^, Li^+^ and Mg^2+^) solution or simulated salt-lake solution with volume of 1 l and DI water with volume of 200 ml were pumped into diluate and concentrate chambers separately, with a volumetric flow rate of 40 ml min^−1^ and current density of 2 mA cm^−2^. The Na_2_SO_4_ solution with volume of 400 ml was pumped into electrode chamber, with volumetric flow rate of 80 ml min^−1^. The experiment normally took several hours with current density 2 mA cm^−2^. Samples were collected every 10 or 20 mins for small electrodialysis stack and every 1 h for large electrodialysis stack. The concentration of salt ions was measured by ICP-MS. The sample solutions were diluted using 2% HNO_3_ appropriately to make sure the concentration is within the detecting limits of ICP-MS.

Two sets of experiments were performed to study the impact of composition of feed solution on the separation performance. In one set of experiments, the concentration of feed solution of mixed salt (Li^+^ and Mg^2+^) were varied at 0.05 M, 0.1 M, 0.25 M, 0.5 M, 0.75 M and 1 M, respectively. A KCl solution of 0.01 M was pumped into the concentrate chambers to reduce the solution resistance. Another set of experiments were performed to study the effect of mass ratio of Mg/Li. The LiCl concentration was fixed at 0.01 mol l^−1^ with Mg/Li mass ratio varied at 10, 20, 40, 60, 80, respectively. The electrode solution fed into electrode chamber in these measurements was 0.3 M Na_2_SO_4_.

To further investigate their performance under different pH conditions, the pH of the feed solution (0.1 M LiCl and MgCl_2_) was adjusted to 2.79, 4, 6.33, 8.79 and 10.23 using H_2_SO_4_ or NaOH. The feed solutions were then pumped into the electrodialysis stack to test ion transport. The testing lasted for a total of 1 h, with samples collected every 10 min. In the stability test, the mixed ion (Li^+^ and Mg^2+^) solution and 0.01 M KCl solution were pumped into diluate and concentrate chambers separately. The electrode solution of 0.3 M Na_2_SO_4_ was pumped into electrode chamber. The experiment took 20 days in total. Each feed solution was changed every 24 hours. For each cycle, samples were collected at time 0, 2 h, 4 h, 6 h and 24 h. The concentration of samples was also quantified by ICP-MS.

The permeation rate is defined as the mole of a specific ion *i* extracted per membrane area per time. The permeation rate of *i*, *P*_*i*,*t*_ (mol m^−2^ h^−1^) from time 0 to time *t* can be calculated using the following equation:

1 $${P}_{i,t}=\frac{{C}_{i,t}{V}_{\mathrm{C},t}-{C}_{i,0}{V}_{\mathrm{C},0}}{{At}}$$

where *C*_*i*,*t*_ refers to the concentration of *i* ion in time *t* in the concentration chamber (mol l^−1^), *V*_C,*t*_ and *V*_C,0_ refer to the volume of the concentrate chamber (l) at time *t* and 0 and *A* is the area of the membrane (m^2^). The errors of calculated ion permeation rates mainly originated from the errors of liner fittings of ion concentrations measured by ICP-MS. Typically, at least three membranes were measured to obtain reliable results.

The selectivity of membranes to other cations M^+^ (K^+^, Na^+^, Li^+^) over Mg^2+^ into concentrate chambers (*S*_M/Mg_) is defined as the ratio of the ion fluxes normalized by their initial concentrations, at time *t*:

2 $${S}_{{\rm{M}}/{\rm{Mg}}}=\frac{{P}_{{\rm{M}},t}}{{P}_{{\rm{Mg}},t}}\frac{{C}_{{\rm{Mg}},t}\,}{{C}_{{\rm{M}},t}}$$

Where *P*_M,*t*_ and *P*_Mg,*t*_ are the permeation rate of M^+^ (K^+^, Na^+^, Li^+^) and Mg^2+^ at time *t* (mol m^−2^ h^−1^), *C*_M_ and *C*_Mg_ are the feed concentrations of M^+^ (K^+^, Na^+^, Li^+^) and Mg^2+^ in the dilute chamber at time *t* (mol l^−1^). The uncertainty of selectivity was derived from the errors of ion permeation rates.

### Molecular modelling

Both equilibrium models and non-equilibrium models were constructed. The construction of the amorphous cell of the pristine AO-PIM-1 and its control derivatives (AO-PIM-De and AO-PIM-Et) was performed with Polymatic^55^. The method of building amorphous polymer models has been widely used in the literature and proven effective in building microporous polymers, such as sulfonated PIM polymers in our previous work^37^, generating valid models with properties (for example, density, porosity) similar to experimental results. A total of 150 monomers were packed in a single system. Five different initial configurations by random packing were prepared to obtain statistically average outcomes. To simulate the hydrated state of polymer membranes, water molecules were added based on the electrolyte uptake of different types of polymer under the condition of 100% relative humidity. Ions were added based on the concentration of 1 M electrolytes. Their respective quantities are listed in Supplementary Table 8.

Molecular dynamics simulations were performed in Large-scale Atomic/Molecular Massively Parallel Simulator^56^. Polymer and ion interactions were described by the optimized potentials for liquid simulations all-atom (OPLS-AA) force field^57^. The LJ parameters of monovalent (K^+^, Na^+^, Li^+^) and divalent (Mg^2+^) ions were taken from the results of Li et al. based on the optimised hydration free energies parameter set^58,59^. TIP3P water model^60^ was used with its bond and angle constrained by the SHAKE algorithm^61^. For the dry polymer model, the polymerized structure underwent a 21-step equilibration process^55^ to obtain an experimentally comparable structure. For the hydrated model, the equilibration scheme was performed after randomly packing water molecules with the polymerized structure.

Non-equilibrium models were also performed and molecular dynamics simulations were carried out to study the ion transport driven by the electric field. The non-equilibrium model is a sandwiched model enveloped by two carbon sheets, which is composed of the electrolyte reservoir, the polymer membrane and the water reservoir. Non-equilibrium simulation of ion transport through the sandwiched model was performed with an electric field of 0.03 V Å^−1^ applied on ions.

Umbrella sampling was used to compute the free energy of ion transporting within the membrane. The path along the *z* axis perpendicular to the membrane cross section starting from the inlet interface to the outlet interface was used as the reaction coordinate, specifically from –21 Å to 123 Å. A harmonic spring of 1.5 kcal mol^−1^ Å^−2^ was employed to steer the transmembrane process of ions. At each step, ions were progressively pulled towards the potential centre for 20 ps, and then 50 ps production run was used for data acquisition. There are a total of 26 windows. The weighted histogram analysis method algorithm was used to generate the free energy profile^62^.

Detailed methods of simulations and data analyses are provided in Supplementary Information.

### Reporting summary

Further information on research design is available in the Nature Portfolio Reporting Summary linked to this article.

## Supplementary information

Supplementary Information Supplementary Methods, Figs. 1–65 and Tables 1–8.

Reporting Summary

## Source data

Source Data Fig. 2 Source data.

Source Data Fig. 3 Source data.

Source Data Fig. 4 Source data.

Source Data Fig. 5 Source data.

## Data Availability

The data supporting the findings of this study are available within the paper and its Supplementary Information. The original Neutron Spin Echo data are available at 10.5291/ILL-DATA.DIR-360. Source data are provided with this paper.

## References

[1] DuChanois, R. M. et al. Prospects of metal recovery from wastewater and brine. *Nat. Water* **1**, 37–46 (2023).

[2] Vera, M. L., Torres, W. R., Galli, C. I., Chagnes, A. & Flexer, V. Environmental impact of direct lithium extraction from brines. *Nat. Rev. Earth Environ.* **4**, 149–165 (2023).

[3] Darling, S. B. The brine of the times. *Science* **385**, 1421–1422 (2024). 39325917 10.1126/science.ads3699

[4] Kazi, O. A. et al. Material design strategies for recovery of critical resources from water. *Adv. Mater.* **35**, 2300913 (2023). 10.1002/adma.202300913 37000538

[5] Li, Z. et al. Lithium extraction from brine through a decoupled and membrane-free electrochemical cell design. *Science* **385**, 1438–1444 (2024). 39325903 10.1126/science.adg8487

[6] Song, Y. et al. Solar transpiration–powered lithium extraction and storage. *Science* **385**, 1444–1449 (2024). 39325897 10.1126/science.adm7034

[7] Chen, X. et al. Spatially separated crystallization for selective lithium extraction from saline water. *Nat. Water* **1**, 808–817 (2023).

[8] Zhang, G. et al. Spontaneous lithium extraction and enrichment from brine with net energy output driven by counter-ion gradients. *Nat. Water* **2**, 1091–1101 (2024).

[9] Wang, R., He, R., He, T., Elimelech, M. & Lin, S. Performance metrics for nanofiltration-based selective separation for resource extraction and recovery. *Nat. Water* **1**, 291–300 (2023).

[10] Foo, Z. H., Thomas, J. B., Heath, S. M., Garcia, J. A. & Lienhard, J. H. Sustainable lithium recovery from hypersaline salt-lakes by selective electrodialysis: transport and thermodynamics. *Environ. Sci. Technol.* **57**, 14747–14759 (2023). 37721998 10.1021/acs.est.3c04472

[11] Wang, R. & Lin, S. Membrane design principles for ion-selective electrodialysis: an analysis for Li/Mg separation. *Environ. Sci. Technol.* **58**, 3552–3563 (2024). 38324772 10.1021/acs.est.3c08956 PMC10882969

[12] Ying, J., Lin, Y., Zhang, Y. & Yu, J. Developmental progress of electrodialysis technologies and membrane materials for extraction of lithium from salt lake brines. *ACS ES&T Water* **3**, 1720–1739 (2023).

[13] Razmjou, A., Asadnia, M., Hosseini, E., Habibnejad Korayem, A. & Chen, V. Design principles of ion selective nanostructured membranes for the extraction of lithium ions. *Nat. Commun.* **10**, 5793 (2019). 31857585 10.1038/s41467-019-13648-7 PMC6923379

[14] Epsztein, R., DuChanois, R. M., Ritt, C. L., Noy, A. & Elimelech, M. Towards single-species selectivity of membranes with subnanometre pores. *Nat. Nanotechnol.* **15**, 426–436 (2020). 32533116 10.1038/s41565-020-0713-6

[15] DuChanois, R. M., Porter, C. J., Violet, C., Verduzco, R. & Elimelech, M. Membrane materials for selective ion separations at the water–energy nexus. *Adv. Mater.* **33**, 2101312 (2021). 10.1002/adma.202101312 34396602

[16] Violet, C. et al. Designing membranes with specific binding sites for selective ion separations. *Nat. Water* **2**, 706–718 (2024).

[17] Gao, F. et al. Pushing the limits of size selectivity in nanoscale solute separations. *Nat. Water* **2**, 521–530 (2024).

[18] Culp, T. E. et al. Nanoscale control of internal inhomogeneity enhances water transport in desalination membranes. *Science* **371**, 72–75 (2021). 33384374 10.1126/science.abb8518

[19] Liang, Y. et al. Polyamide nanofiltration membrane with highly uniform sub-nanometre pores for sub-1 Å precision separation. *Nat. Commun.* **11**, 2015 (2020). 32332724 10.1038/s41467-020-15771-2 PMC7181833

[20] Zhou, X. et al. Intrapore energy barriers govern ion transport and selectivity of desalination membranes. *Sci. Adv.* **6**, eabd9045 (2020). 33239305 10.1126/sciadv.abd9045 PMC7688318

[21] Slater, A. G. & Cooper, A. I. Function-led design of new porous materials. *Science* **348**, aaa8075 (2015). 26023142 10.1126/science.aaa8075

[22] Zuo, P. et al. Near-frictionless ion transport within triazine framework membranes. *Nature* **617**, 299–305 (2023). 37100908 10.1038/s41586-023-05888-x PMC10131500

[23] Lu, J. et al. Efficient metal ion sieving in rectifying subnanochannels enabled by metal–organic frameworks. *Nat. Mater.* **19**, 767–774 (2020). 32152561 10.1038/s41563-020-0634-7

[24] Xu, T. et al. Perfect confinement of crown ethers in MOF membrane for complete dehydration and fast transport of monovalent ions. *Sci. Adv.* **10**, eadn0944 (2024). 38718127 10.1126/sciadv.adn0944 PMC11078184

[25] Sheng, F. et al. Efficient ion sieving in covalent organic framework membranes with sub-2-nanometer channels. *Adv. Mater.* **33**, 2104404 (2021). 10.1002/adma.202104404 34480387

[26] Meng, Q.-W. et al. Enhancing ion selectivity by tuning solvation abilities of covalent-organic-framework membranes. *Proc. Natl Acad. Sci. USA* **121**, e2316716121 (2024). 38349874 10.1073/pnas.2316716121 PMC10895279

[27] Meng, W. et al. Three-dimensional cationic covalent organic framework membranes for rapid and selective lithium extraction from saline water. *Nat. Water*10.1038/s44221-024-00379-3 (2025).

[28] Xu, T. et al. Highly ion-permselective porous organic cage membranes with hierarchical channels. *J. Am. Chem. Soc.* **144**, 10220–10229 (2022). 35586909 10.1021/jacs.2c00318

[29] Abraham, J. et al. Tunable sieving of ions using graphene oxide membranes. *Nat. Nanotechnol.* **12**, 546–550 (2017). 28369049 10.1038/nnano.2017.21

[30] Ries, L. et al. Enhanced sieving from exfoliated MoS_2_ membranes via covalent functionalization. *Nat. Mater.* **18**, 1112–1117 (2019). 31451779 10.1038/s41563-019-0464-7

[31] Budd, P. M. et al. Solution-processed, organophilic membrane derived from a polymer of intrinsic microporosity. *Adv. Mater.* **16**, 456–459 (2004).

[32] Carta, M. et al. An efficient polymer molecular sieve for membrane gas separations. *Science* **339**, 303–307 (2013). 23329042 10.1126/science.1228032

[33] Guiver, M. D. & Lee, Y. M. Polymer rigidity improves microporous membranes. *Science* **339**, 284–285 (2013). 23329040 10.1126/science.1232714

[34] Rose, I. et al. Polymer ultrapermeability from the inefficient packing of 2D chains. *Nat. Mater.* **16**, 932–937 (2017). 28759030 10.1038/nmat4939

[35] Tan, R. et al. Hydrophilic microporous membranes for selective ion separation and flow-battery energy storage. *Nat. Mater.* **19**, 195–202 (2020). 31792424 10.1038/s41563-019-0536-8

[36] Ye, C. et al. Long-Life aqueous organic redox flow batteries enabled by amidoxime-functionalized ion-selective polymer membranes. *Angew. Chem. Int. Ed.* **61**, e202207580 (2022). 10.1002/anie.202207580 PMC9541571 35876472

[37] Ye, C. et al. Development of efficient aqueous organic redox flow batteries using ion-sieving sulfonated polymer membranes. *Nat. Commun.* **13**, 3184 (2022). 35676263 10.1038/s41467-022-30943-y PMC9177609

[38] Wang, A. et al. Ion-selective microporous polymer membranes with hydrogen-bond and salt-bridge networks for aqueous organic redox flow batteries. *Adv. Mater.* **35**, 2210098 (2023). 10.1002/adma.202210098 36634684

[39] Baran, M. J. et al. Design rules for membranes from polymers of intrinsic microporosity for crossover-free aqueous electrochemical devices. *Joule* **3**, 2968–2985 (2019).

[40] Wang, A. et al. Selective ion transport through hydrated micropores in polymer membranes. *Nature* **635**, 353–358 (2024). 39506120 10.1038/s41586-024-08140-2 PMC11560840

[41] Patel, H. A. & Yavuz, C. T. Noninvasive functionalization of polymers of intrinsic microporosity for enhanced CO_2_ capture. *Chem. Commun.* **48**, 9989–9991 (2012). 10.1039/c2cc35392j 22951579

[42] Du, N., Robertson, G. P., Song, J., Pinnau, I. & Guiver, M. D. High-performance carboxylated polymers of intrinsic microporosity (PIMs) with tunable gas transport properties. *Macromolecules* **42**, 6038–6043 (2009).

[43] Mizrahi Rodriguez, K. et al. Facile and time-efficient carboxylic acid functionalization of PIM-1: effect on molecular packing and gas separation performance. *Macromolecules* **53**, 6220–6234 (2020).

[44] Moh, L. C. H., Goods, J. B., Kim, Y. & Swager, T. M. Free volume enhanced proton exchange membranes from sulfonated triptycene poly(ether ketone). *J. Membr. Sci.* **549**, 236–243 (2018).

[45] Wong, T. et al. Sulfonated poly(ether-ether-ketone) membranes with intrinsic microporosity enable efficient redox flow batteries for energy storage. *Joule* **9**, 101795 (2025).

[46] Cseri, L. et al. Electrospun adsorptive nanofibrous membranes from ion exchange polymers to snare textile dyes from wastewater. *Adv. Mater. Technol.* **6**, 2000955 (2021).

[47] Perrin, J.-C., Lyonnard, S. & Volino, F. Quasielastic neutron scattering study of water dynamics in hydrated nafion membranes. *J. Phys. Chem. C* **111**, 3393–3404 (2007).

[48] Foglia, F. et al. Disentangling water, ion and polymer dynamics in an anion exchange membrane. *Nat. Mater.* **21**, 555–563 (2022). 35301475 10.1038/s41563-022-01197-2

[49] Ma, H. et al. Dual-channel-ion conductor membrane for low-energy lithium extraction. *Environ. Sci. Technol.* **57**, 17246–17255 (2023). 37918342 10.1021/acs.est.3c05935

[50] Warnock, S. J. et al. Engineering Li/Na selectivity in 12-crown-4–functionalized polymer membranes. *Proc. Natl Acad. Sci. USA* **118**, e2022197118 (2021). 34493651 10.1073/pnas.2022197118 PMC8449368

[51] Yong, M. et al. Sustainable lithium extraction and magnesium hydroxide co-production from salt-lake brines. *Nat. Sustain.* **7**, 1662–1671 (2024).

[52] Du, N., Song, J., Robertson, G. P., Pinnau, I. & Guiver, M. D. Linear high molecular weight ladder polymer via fast polycondensation of 5,5′,6,6′-tetrahydroxy-3,3,3′,3′-tetramethylspirobisindane with 1,4-dicyanotetrafluorobenzene. *Macromol. Rapid Commun.* **29**, 783–788 (2008).

[53] Baran, M. J. et al. Diversity-oriented synthesis of polymer membranes with ion solvation cages. *Nature* **592**, 225–231 (2021). 33828319 10.1038/s41586-021-03377-7

[54] Huang, F. et al. Preparation of amidoxime polyacrylonitrile chelating nanofibers and their application for adsorption of metal Ions. *Materials* **6**, 969–980 (2013). 28809351 10.3390/ma6030969 PMC5512958

[55] Abbott, L. J., Hart, K. E. & Colina, C. M. Polymatic: a generalized simulated polymerization algorithm for amorphous polymers. *Theor. Chem. Acc.* **132**, 1334 (2013).

[56] Thompson, A. P. et al. LAMMPS—a flexible simulation tool for particle-based materials modeling at the atomic, meso, and continuum scales. *Comput. Phys. Commun.* **271**, 108171 (2022).

[57] Jorgensen, W. L., Maxwell, D. S. & Tirado-Rives, J. Development and testing of the OPLS all-atom force field on conformational energetics and properties of organic liquids. *J. Am. Chem. Soc.* **118**, 11225–11236 (1996).

[58] Li, P., Roberts, B. P., Chakravorty, D. K. & Merz, K. M. Jr Rational design of Particle Mesh Ewald compatible Lennard-Jones parameters for +2 metal cations in explicit solvent. *J. Chem. Theory Comput.* **9**, 2733–2748 (2013). 10.1021/ct400146w PMC3728907 23914143

[59] Li, P., Song, L. F. & Merz, K. M. Jr Systematic parameterization of monovalent ions employing the nonbonded model. *J. Chem. Theory Comput.* **11**, 1645–1657 (2015). 10.1021/ct500918t 26574374

[60] Jorgensen, W. L., Chandrasekhar, J., Madura, J. D., Impey, R. W. & Klein, M. L. Comparison of simple potential functions for simulating liquid water. *J. Chem. Phys.* **79**, 926–935 (1983).

[61] Andersen, H. C. Rattle: a ‘velocity’ version of the shake algorithm for molecular dynamics calculations. *J. Comput. Phys.* **52**, 24–34 (1983).

[62] Grossfield, A. WHAM: the weighted histogram analysis method, version 2.0.10 http://membrane.urmc.rochester.edu/wordpress/?page_id=126 (2020).

