# Supplementary material for: Solution-processable polymer membranes with hydrophilic subnanometre pores for sustainable lithium extraction
Source: Nat Water. 2025 Mar 12;3(3):319–33. doi: 10.1038/s44221-025-00398-8 (PMC11932922; doi:10.1038/s44221-025-00398-8)
Supplement: Supplementary file 1 — Supplementary Methods, Figs. 1–65 and Tables 1–8. [file 44221_2025_398_MOESM1_ESM.pdf]

nature water

Supplementary information https://doi.org/10.1038/s44221-025-00398-8

Solution-processable polymer membranes with hydrophilic subnanometre pores for sustainable lithium extraction

In the format provided by the authors and unedited

Supplementary Methods
Materials

Most chemicals were purchased from Sigma Aldrich or TCI Chemicals and used without further purification unless otherwise noted. Solvents were purchased from VWR UK. Commercial Nafion membranes and PiperION AEM membranes were purchased from Fuel Cell Store. The monomers 6,6'-dimethoxy-3,3,3', 3'-tetramethyl-2,2', 3,3'-tetrahydro-1,1'-spiro (SBI-OMe) and 3,3,3′,3′-Tetramethyl-1,1′-spirobisindane-6,6′-diol (SBI-OH) were synthesized and purified following the literature[1].

Synthesis of SBI-OH. In a 500 ml round bottom flask under nitrogen flow, 200 g (870 mmol) of bisphenol A (BPA) was added and the flask heated to 135 ^{o}C to melt the solid. Then, 10 mL of methansulfonic acid was added and the solution was stirred for 6h. The mixture was cooled to room temperature and then poured into vigorously stirred water for 2 h. The yellow precipitate was collected by filtration and dried overnight under vacuum. The yellow solid was recrystallized from a 1:1 water/ethanol mixture to yield 3,3,3′,3′-Tetramethyl-1,1′-spirobisindane-6,6′-diol (SBI-OH) as white crystals which were dried overnight under vacuum at 100 ^{o}C.

Synthesis of SBI-OMe. In a 200 ml round bottom flask submerged in an ice bath, 20 g (65 mmol) of SBI-OH was dissolved in acetone under mechanical stirring. 45 g of potassium carbonate was added, and the mixture was stirred for 30 min. Then 41 g (325 mmol) of dimethyl sulfate was added dropwise and the reaction was stirred for 12 h. The mixture was poured onto ice under stirring and the white precipitate was collected by filtration. The white solids were washed repeatedly with water. The product was purified by recrystallisation from petroleum ether to obtain 6,6'-dimethoxy-3,3,3',3'-tetramethyl-2,2',3,3'-tetrahydro-1,1'-spiro (SBI-OMe) as white crystals.

Synthesis of PIM-1. The synthesis of PIM-1 was adapted from the literature[2]. Purified monomers including 5,5′,6,6′-Tetrahyroxy-3,3,3′,3′-tetramethyl-1,1′-spirobisindane (TTSBI) (3.40 g, 10.0 mmol) and 2,3,5,6- tetrafluoroterephthalonitrile (TFTPN) (2.00 g, 10.0 mmol) were added to a 250 mL three-neck-round-bottomed flask and pre-flashed with nitrogen, before being dissolved in anhydrous DMAc (18.0 mL). Once a clear solution formed, the flask was placed under reflux at 150˚C, followed by the addition of K_2CO_3 fine powder (3 × 1.17 g, 25.4 mmol) over 5 minutes, where the resulting solution increased significantly in viscosity after 0.5 h. Subsequently, three portions of toluene (3 × 2 mL) were added over 10 mins, and the solution was left to stir for 1 h. The polymer solution was poured into methanol (200 mL) and left to stir. After filtering and drying overnight, the solid was dissolved in chloroform and reprecipitated in methanol. Finally, the polymer was refluxed in water overnight to remove any residual salts and dried in a vacuum oven at 110˚C for 12h to give PIM-1 as a yellow powder at 94% yield.

Synthesis of AO-PIM-1. The AO-PIM-1 polymer was synthesized following the protocol reported in the literature[3]. PIM-1 (20.00 g, 38.4 mmol) and THF (800 mL) were added to a glass reactor (Asynt, UK). Once dissolved, the solution was set to reflux at 69˚C under argon using a waterless condenser. A solution of 50 wt% aqueous hydroxylamine (200 mL) was then added dropwise to allow the precipitate to redissolve. Stirring was maintained for a further 20 h, and the solution was precipitated into ethanol (3 L). The solid was filtered under vacuum and then rinsed thoroughly with DI water and ethanol before drying overnight at 110 °C to give AO-PIM-1 as a pale-yellow powder (19.77 g) in 87% yield. ^{1}H NMR

(d6-DMSO, 400 MHz): δ (ppm) 9.44 (br t, 2H), 6.80 (br s, 2H), 6.13 (br s, 2H), 5.77 (br s, 4H), 2.07 (br d, 4H), 1.30 (br s, 6H), 1.22 (br s, 6H);

Synthesis of AO-PIM-1-Et. The AO-PIM-1-Et was synthesized following a previous study[4]. AO-PIM-1 (1.37 g, 2.6 mmol) and dimethyl sulfoxide (57 mL) were added to a round bottom flask pre-flashed with N_2. The solution was heated gently to allow the polymer to dissolve. Once cooled, a solution of lithium hydroxide monohydrate (0.48 g, 11.4 mmol) in DI water (5.75 mL) was added dropwise over 1 min. The resulting solution was left to stir for 1 h. The reaction mixture was then placed on ice, and diethyl sulfate (1.76 g, 11.4 mmol) was added dropwise. The reaction mixture was allowed to reach room temperature and stirred for a further 72 h. Sodium hydroxide was then added to quench the reaction and increase the pH above 7. The solution was precipitated in DI water, filtered under vacuum and rinsed abundantly with DI water. The final product was dried overnight at 100˚C to give AO-PIM-Et as a dark yellow solid (1.19 g) in 78% yield. 1 H NMR (d6-DMSO, 400 MHz): δ (ppm) 6.75 (br s, 2H), 6.16 (br s, 2H), 5.78 (br s, 4H), 3.87(br t, 4H), 2.23 (br d, 4H), 1.29–1.06 (br m, 18H).

Synthesis of AO-PAN. AO-PAN membranes were made through AO modification of PAN membranes. PAN polymer was dissolved into DMSO solvent and cast into membrane. Then PAN membranes were modified into AO-PAN membranes by reacting with hydroxylamine solution, which converts nitrile groups into amidoxime groups. Dried PAN membrane samples were immersed in 50 mL of an aqueous hydroxylamine solution under N_2 for a specified duration. Parameters of 60 °C, 3 h, and 5 g L^{-1} hydroxylamine solution were reported to be the optimal amidoximation condition[5]. After the reaction, the membranes were thoroughly washed multiple times with deionized water.

Synthesis of blended AO-PAN/PIM-SBI-OMe-AO membranes: The blend membrane was prepared through AO modification of blended PAN/PIM-SBI-OMe-CN membranes. PAN and PIM-SBI-OMe-CN polymers were blended with 1:1 ratio and dissolved in DMSO solvent. After fully stirred, the blended polymer solution was cast into membrane. Dried PAN/PIM-SBI-OMe-CN membrane samples were immersed in 50 mL of an aqueous hydroxylamine solution with concentration of 5 g L^{-1} under N_2 for 60 °C for 3h. After the reaction, the AO-PAN/PIM-SBI-OMe-AO membranes were thoroughly washed multiple times with deionized water.

Synthesis of cPIM-1. The cPIM-1 was synthesized following a previous study[6]. To a 1L round-bottomed flask containing PIM-1 (10 g) as a fine powder, DI water (250 mL), glacial acetic acid (83 mL) and concentrated sulfuric acid (250 mL) were added in sequence. The reaction flask was refluxed at 150˚C for 48 h. The solution was then cooled, and vacuum filtered directly. The powder was washed abundantly with DI water, with the pH being checked throughout. The powder was then transferred to a 1 L round-bottomed flask containing 0.1 M aqueous sulfuric acid (500 mL) and heated to reflux overnight. The powder was then filtered, washed abundantly with DI water, and dried overnight to give cPIM-1 as a dark orange powder at 95% yield.

Synthesis of cPIM-1-OMe. To a two-neck round bottomed flask containing cPIM-1 (0.5 g, 1.00 mmol), K_2CO_3 (1.11 g, 8.02 mmol) and anhydrous DMF (12.5 mL) were mixed and stirred at room temperature under argon atmosphere. An excess of Me-I (1.42 g, 10.03 mmol) was added in the dark under vigorous stirring. The solution was left to stir overnight and was precipitated in DI water. The polymer was washed

abundantly in DI water at least twice, filtered under vacuum and dried overnight to give cPIM-1-OMe as a yellow powder at 90 % yield.

Synthesis of PIM-SBI-OMe-CN. In a 100 mL round bottom flask with a magnetic stirrer, 1.70 g (13 mmol) of 4-formylbenzonitrile was dissolved in 20 mL dichloromethane. 3.06 g (10mmol) of SBI-OMe was added to form a clear brown solution. Then 5 mL of methanesulfonic acid was added dropwise. The reaction was left to stir for 30 minutes when a viscous solution was formed. The solution was poured into stirred methanol to precipitate a fibrous white polymer (PIM-SBI-OMe-CN). The polymer was washed with methanol followed by boiling water to remove residual acid before drying overnight in air at 100 ^{o}C. The dried polymer was dissolved in DCM and precipitated in methanol to obtain the purified polymer as a white powder.

Synthesis of PIM-SBI-OMe-AO. In a 250 ml two-neck round-bottom flask equipped with a reflux condenser and pressure actuated dropping funnel, 2 g (4.16 mmol) of PIM-SBI-OMe-CN polymer was dissolved in 80 mL of DMSO. The polymer solution was heated to 90 ^{o}C under stirring. Then, 30 mL of hydroxylamine solution was added dropwise via the dropping funnel. On complete addition, the reaction mixture was left to stir overnight at 100 ^{o}C before allowing to cool to room temperature. Once cooled, the precipitated polymer was obtained by filtration and washed repeatedly with water before drying overnight under vacuum at 100 ^{o}C to yield PIM-SBI-OMe-AO as a straw colour powder.

Synthesis of PIM-SBI-OH-CN. In a 100 mL round bottom flask with a magnetic stirrer, 1.70 g (13 mmol) of 4-formylbenzonitrile was dissolved in 20 mL dichloromethane. 3.36 g (10 mmol) of SBI-OH was added. Then 3 mL of methanesulfonic acid was added dropwise. The reaction was left to stir for 10 minutes when a viscous solution was formed. The solution was poured into stirred water to precipitate a fibrous white polymer (PIM-SBI-OH-CN). The polymer was washed repeatedly with boiling water to remove residual acid before drying overnight in air at 100 ^{o}C. The dried polymer was dissolved in THF and precipitated in methanol to obtain the purified polymer as a white powder.

Synthesis of PIM-SBI-OH-AO. In a 250 mL two-neck round-bottom flask equipped with a reflux condenser and pressure actuated dropping funnel, 2 g (4.46 mmol) of PIM-SBI-OH-CN polymer was dissolved in 100 mL of DMSO. The polymer solution was heated to 90 ^{o}C under stirring. Then, 30 mL of hydroxylamine solution was added dropwise via the dropping funnel. On complete addition, the reaction mixture was left to stir overnight at 100 ^{o}C before allowing to cool to room temperature. Once cooled, the precipitated polymer was obtained by filtration and washed repeatedly with water before drying overnight under vacuum at 100 ^{o}C to yield PIM-SBI-OH-AO as a light brown solid.

Synthesis of cPIM-Et. AO-PIM-1 (2.00 g, 3.8 mmol repeat unit) was dissolved in 100 mL DMSO, and succinic anhydride (1.9 g, 5 mol eq. relative to the repeating unit of AO-PIM-1) was added into the AO-PIM-1 solution. After the full dissolution of anhydride, the mixture was stirred at 30 ^{o}C for a further 4 h and then potassium ethoxide was added (4.15 g, 13 mol 90 eq. relative to the repeating unit of AO-PIM-1). The mixture was vigorously stirred at room temperature for 1 h and then poured into 400 mL water. Hydrochloric acid was added dropwise to the solution until pH was adjusted to 1-2. The precipitate was filtered, suspended in 0.5 M aqueous H_2SO_4, and refluxed for 4 hours. The powder was then collected by filtration, rinsed with deionized water and acetone, and briefly air-dried at 110 °C for 1 hour to yield a free-flowing yellow powder.

Synthesis of cPIM-Ph. The synthetic route is similar to cPIM-Et. AO-PIM-1 (2.00 g, 3.8 mmol repeat unit) was dissolved in 100 mL DMSO, and phthalic anhydride (2.8 g, 5 mol eq. relative to the repeating unit of AO-PIM-1) was added into the AO-PIM-1 solution. Once the anhydride was fully dissolved, the mixture was stirred at 30 °C for an additional 4 hours, after which potassium ethoxide (4.15 g, 13 mmol, 90 equivalents relative to the repeating unit of AO-PIM-1) was added. The mixture was stirred vigorously at room temperature for 1 hour and then poured into 400 mL of water. Hydrochloric acid was added dropwise until the pH was adjusted to 1-2. The precipitate was filtered, suspended in 0.5 M aqueous H_2SO_4, and refluxed for 4 hours. The powder was subsequently collected by filtration, washed with deionized water and acetone, and briefly air-dried at 110 °C for 1 hour, yielding a free-flowing yellow powder.

Synthesis of sPIM-1-ES. sPIM-1-ES was prepared by reacting cPIM-1 (2.00 g, 3.58 mmol repeating unit) with 1,3-propanesultone (1093 mg, 8.95 mmol) in DMF (30 mL). Then K_2CO_3 (1237 mg, 8.95 mmol) was added into the solution. The reaction solution was stirred at 50 °C for 24 h. After cooling down to the room temperature, the mixture was precipitated by adding a 1 M HCl solution. The precipitated polymer was washed by water and cold ethanol, then dried in a vacuum overnight. The polymer obtained was a yellow-brown powder.

Synthesis of sPIM-Ph-ES. sPIM-Ph-ES was prepared by reacting cPIM-Ph (1.00 g, 1.22 mmol repeating unit) with 1,3-propanesultone (373 mg, 3.05 mmol) in DMF (30 mL). Then K_2CO_3 (421.5 mg, 3.05 mmol) was added into the solution. The reaction solution was stirred at 50 °C for 24 h. After cooling down to the room temperature, the mixture was precipitated by adding a 1 M HCl solution. The precipitated polymer was washed by water and cold ethanol, then dried in a vacuum overnight. The polymer obtained was a yellow powder.

Membrane fabrication

Polymer membranes were fabricated by casting polymer solutions on glass plates with a doctor blade (Elcometer, UK). For AO-PIM-1 membrane, polymer solution was prepared by dissolving polymers (2 g) in DMF (8.0 mL – 12.0 mL). Before casting, the solution was centrifuged. The membrane was obtained after heating the glass plate at 60°C in an oven for 2 days and immersed in DI water. For AO-PIM-1-Et membrane, polymer solution was prepared by dissolving polymers (1.5 g) in DMSO (15.0 g – 25.0 g depending on viscosity). The membrane was obtained after heating the glass plate at 80 ^{o}C in an oven for 12h and immersed in DI water. AO-PIM-1-De membrane was obtained by soaking the AO-PIM-1 membrane in 1 M alkaline solutions (KOH or NaOH) for 12 hours. PIM-SBI-OMe-CN, PIM-SBI-OH-AO and PIM-SBI-OMe-AO membranes were prepared using the same solution casting method. The polymer solutions were prepared by dissolving polymers (1.5 g) in DMSO (15.0 g – 25.0 g depending on viscosity) at 60°C and then casted on glass plates. The membranes were obtained after heating the glass plate at 70°C in an oven for 12h and immersed in DI water. cPIM-1, cPIM-Et, and cPIM-Ph membranes were prepared by dissolving 1g polymers in DMSO (10.0 g – 20.0 g depending on viscosity) at 50°C and heated at 60°C in an oven for two days and immersed in DI water. sPIM-1-ES and sPIM-Ph-ES membranes were prepared by dissolving 0.5g polymers in DMSO (5.0 g – 10.0 g depending on viscosity) at 50°C and heated at 60°C in an oven for two days and immersed in DI water.

NMR spectroscopy

NMR experiments were performed on a Bruker Avance III spectrometer equipped with a 7.0 T superconducting magnet operating at a ^1H frequency of 300.13 MHz and at a sample temperature of 302.5 ± 0.3 K unless stated otherwise. ^1H pulsed gradient stimulated echo (PGSTE) NMR was performed using a 5 mm ^1H radiofrequency coil, in a Bruker diff30 probe with a maximum gradient strength of 17.7 T m^{-1}. ^1H NMR spectra were acquired for each hydrated membrane sample with a 10 kHz spectral width, four signal averages and a repetition time of 5 s. Polymer films were exposed to water, 0.1 M LiCl, 0.1 M $MgCl_2$, or mixture of 0.1 M LiCl and 0.1 M $MgCl_2$, for overnight. The excess surface water was wiped off with tissue paper and then the membranes were rolled up and placed into NMR glass tubes (Norell, 5 mm). The water self-diffusion coefficients were derived following the same protocols reported in a recent study[7].

Neutron scattering

The Wide Angle Neutron Spin-Echo (WASP) spectrometer (https://www.ill.eu/users/instruments/instruments-list/wasp/description/instrument-layout) at ILL, Grenoble, employs uniquely shaped copper solenoid coils arranged in an anti-Helmholtz configuration to create a cylindrically symmetric magnetic field around the sample position. This setup achieves a field integral of up to 0.22 Tm in its current configuration. Using these magnetic coils, the nuclear spin of probe neutrons can be manipulated with high precision, enabling the neutron spin to act as an internal clock of remarkable accuracy. With a neutron wavelength of $\lambda = 6$ Å, as used in this experiment, we measured sample dynamics within the time range of $\tau = 4$ ps to 8 ns, covering momentum transfers from Q = 0.6 to 1.6 Å^{-1}.

Samples were loaded into aluminum annular cans, and scattering profiles were acquired at a temperature of 300 K. Data analysis addressed both polymer and water dynamics, with polymer dynamics modelled using a series of stretched exponential functions. These functions corresponded to two distinct polymer dynamics - one on the tens of picoseconds timescale and another on the hundreds of picoseconds timescale - with an additional nearly flat background (sub-ps timescale) included to best fit the data.

Water dynamics was analyzed using a Gaussian model to capture both localized and long-range behavior. The combined model was formulated by weighting contributions according to water uptake, as previously reported for other technologically significant membranes[7-9].

Electrolyte uptake

The electrolyte uptake (EU) or water uptake (WU) of the membrane is defined as the weight difference between the wet membrane after soaking in corresponding salt solution or DI water and the dry membrane. The dry membrane was obtained after drying in an oven at 60 °C for more than 24h. Electrolyte uptake and water uptake are calculated using the following equation:

$$EU/WU = \left(\frac{W_{wet} - W_{dry}}{W_{dry}}\right) \cdot 100\% \quad (1)$$

where W_{wet} and W_{dry} are the wet and dry membranes' weight, respectively. The standard deviations were derived from at least three membranes.

Swelling ratio

The swelling ratio (SR) of membranes is defined as the length difference between the wet membrane and the dry membrane. Swelling ratio is calculated using the following equation:

$$SR = \left(\frac{L_{wet} - L_{dry}}{L_{dry}}\right) \cdot 100\% \qquad (2)$$

where L_{wet} and L_{dry} are the wet and dry membranes' length, respectively. The standard deviations were derived from at least three membranes.

Ion conductivity

Through-plane membrane resistance was assessed through a two-electrode electrochemical impedance spectroscopy (EIS) technique utilizing a potentiostat, employing an AC bias of 10 mV and scanning frequencies ranging from 0.2 MHz to 10 Hz. Membranes saturated with an aqueous electrolyte were positioned between two stainless steel electrodes, each featuring an effective area of 2 cm^2, and securely enclosed within coin cells for ionic conductivity evaluations under elevated temperatures. The assembly procedure was conducted within the electrolyte solution to prevent the entrapment of air bubbles within the coin cells.

Ion conductivity of membranes can be calculated using the following equation:

$$\sigma = \frac{L}{RA} * 1000 \qquad (3)$$

where σ is the ion conductivity (mS cm^{-1}), L is the thickness of the membrane (cm), A is the effective area of membrane (cm^2), and R is the membrane resistance from Nyquist plots figures (Ω). The standard deviations were derived from at least three membranes.

Ion transference number

In order to test ion transference number, a two-chamber H-cell by one membrane was used. In this study, the transport of four cations (K^+, Na^+, Li^+, and Mg^{2+}) was investigated, thus four groups of solutions (0.1 M KCl/1 M KCl, 0.1 M NaCl/1 M NaCl, 0.1 M LiCl/1 M LiCl and 0.1 M $MgCl_2$/1 M $MgCl_2$) with both volumes of 10 mL were measured. Ag–AgCl electrodes were put in both cells, and the apparatus was left for about 30 mins to reach a steady state. Using the Linear sweep voltammetry (LSV) method to measure the potential by connecting the two Ag-AgCl electrodes and recording the membrane potential (the voltage value corresponding to current density 0). Apparent ion transference number (t) is calculated from the zero-current potential (V_0), which equals the membrane potential, based on the following equation:

$$V_0=[\frac{t_+}{z_+}-\frac{t_-}{z_-}][\frac{kT}{e}]\ln[\Delta C\frac{\gamma_{high}}{\gamma_{low}}] \qquad (4)$$

where k is the Boltzmann constant, T is the temperature (K), e is the elementary charge, ΔC is the ratio of high concentration to low concentration (ΔC = 10), γ_{high} and γ_{low} are the activity coefficients of the high-concentration solution and low-concentration solution[37], respectively.

Concentration-driven diffusion dialysis

To study the ion permeation through membranes, we performed a series of dialysis experiments using H-cell, with binary salt in feed solution. The volume of each cell is 100 mL. The effective area of membrane is 1.4 cm^2. Samples were collected regularly to measure the concentration of each cation by using Inductively coupled plasma mass spectrometry (ICP-MS).

The permeation rate is defined as the mole of a specific ion i extracted per membrane area per time. The permeation rate of i, $P_{i,t}$ (mol m^{-2} h^{-1}) from time 0 to time t can be calculated using the following equation:

$$P_{i,t} = \frac{C_{i,t}V_{C,t} - C_{i,0}V_{C,0}}{At} \quad (5)$$

Where $C_{i,t}$ refers to the concentration of i ion in the concentrate chamber (mol L^{-1}) at time t, $V_{C,t}$ and $V_{C,0}$ refer to the volume of the concentrate chamber (L) at time t and 0, and A is the effective area of the membrane (m^2). The error was derived from the linear fittings of the concentration profile. The uncertainty of selectivity is derived from that of ion permeation rates.

Energy Consumption

The specific energy consumption (SEC_{Li}[kWh mol^{-1}]), defined as the electrical work required to produce one mole of lithium, is calculated using the following equation:

$$SEC_{Li} = \frac{IA\int_0^t U_t dt}{C_{Li,t}\, V_{C,t} - C_{Li,0}\, V_{C,0}} \quad (6)$$

Where U_t is the applied voltage across the ED stack at time t. I (A m^{-2}) is the current density, A is the effective area (m^2); dt is the time interval for voltage recording. The voltage integral was approximated numerically with a 1st-order Backward-Euler scheme. $C_{Li,t}$ (mol L^{-1}) and $C_{Li,0}$ (mol L^{-1}) denote the concentrations of extracted Li^+ in concentrate chamber at time t and 0, respectively, while $V_{C,t}$ (L) and $V_{C,0}$ (L) represent the volumes of concentrate solutions at time t and 0, respectively.

Li_2CO_3 precipitation and purification

After the large-scale electrodialysis test, the concentrate solution, initially 200 mL in volume, was evaporated and reduced to approximately 10 mL. Li_2CO_3 was precipitated by adding 10 mL saturated Na_2CO_3 solution at 80°C. The resulting Li_2CO_3 precipitate was then washed with hot water (80°C) several times to remove impurities. After washing, the Li_2CO_3 powder was placed in an oven at 100°C for overnight. The ion composition of the solid was measured by ICP-MS. The X-ray diffraction pattern was obtained using a PANalytical X'Pert Pro diffractometer with Cu Kα radiation. The sample was scanned over a 2θ range of 3° to 90° at a step size of 0.02° and a counting time of 1s per step. The diffraction pattern obtained was analyzed to identify the crystalline phases present in the sample. The peak positions and intensities were also compared to standard reference patterns.

Equilibrium models

The construction of the amorphous cell of the pristine AO-PIM-1 and its control derivatives (AO-PIM-De and AO-PIM-Et) was performed with Polymatic [10]. The method of building amorphous polymer models has been widely used in the literature and proven effective in building microporous polymers, such as sulfonated PIM polymers in our previous work[11], generating valid models with properties (e.g.

density, porosity) similar to experimental results. The generation steps were as follows: 1) a structure with two repeating units was created and optimised by the Hartree-Fock method with the 6-31G* basis set; 2) atomic charges were computed via CHELPG method with MP2 theory coupled with cc -PVTZ(-f) basis set; 3) the monomer was extracted from the optimised structure and was employed as the starting configuration for the generation process via Polymatic. Specifically for the deprotonated AO-PIM-1 (AO-PIM-De), the charges were manually redistributed to ensure the net charge of N-O group is -1 e. A total of 150 monomers were packed in a single system. 5 different initial configurations by random packing were prepared to obtain statistically average outcomes. To simulate the hydrated state of polymer membranes, water molecules were added based on the electrolyte uptake of different types of polymers under the condition of 100% relative humidity. Salt ions were added based on the concentration of 1 M electrolytes. Their respective quantities are listed in Supplementary Table S8.

Molecular dynamics simulations were performed in Large-scale Atomic/Molecular Massively Parallel Simulator (LAMMPS)[12]. Polymer and ion interactions were described by the OPLS-AA force field[13]. The LJ parameters of monovalent (K^+, Na^+, Li^+) and divalent (Mg^{2+}) ions were taken from the results of Li et al. based on the optimised hydration free energies (HFE) parameter set[14, 15]. TIP3P water model [16] was used with its bond and angle constrained by the SHAKE algorithm[17]. A short-range cutoff of 12 Å was used for non-bonded interactions while the Particle-Particle Particle-Mesh solver (PPPM) with an accuracy of 3×10^{-5} was used for long-range interactions. Velocity-Verlet algorithm, with a timestep of 1 fs, was employed for time integration throughout the simulation. Temperature and pressure were regulated using a Nosé–Hoover thermostat and barostat within the *NVT* and *NPT* ensembles, with time constants of 0.1 ps. For the dry polymer model, the polymerized structure underwent a 21-step equilibration process [10] to obtain an experimentally comparable structure. For the hydrated model, the equilibration scheme was performed after randomly packing water molecules with the polymerized structure. A production run of 20 ns at 300 K NVT was performed based on the equilibrated structure. Mean squared displacement (MSD) was plotted every 10 ps over a trajectory of 20 ns. Self-diffusion coefficients (D_{self}) were then extracted from the slope of the linear portion of the MSD according to the Einstein relation.

Non-equilibrium models and molecular dynamics simulations

The non-equilibrium model is a sandwiched model enveloped by two carbon sheets, which is composed of the electrolyte reservoir, the polymer membrane, and the water reservoir. The components were prepared separately. 1) The construction of the polymer membrane employed the same polymerization and equilibration method described in the Equilibrium model section. Water molecules were added based on the water uptake of polymers at fully hydrated state. During the simulated polymerization and equilibration, wall conditions using 12-6 LJ potential were implemented on the z direction to prevent monomers and water molecules from crossing the z boundary. More specifically, two virtual flat walls were placed at the z boundary of the polymer cell. The strength, size, and cut-off distances for wall-particle interaction were determined as 1 kcal mol^{-1} $Å^{-2}$, 1 Å, and 1 Å respectively. 2) The water cell was prepared using the lattice method where the number of water molecules and the box dimension were determined to ensure the density close to 0.982 g cm^{-3}. This is for consistency with the target density where the TIP3P water model was originally parameterised[16]. The initial water cell was rescaled to the final target density while ensuring its final x and y dimension were identical to those of the equilibrated

polymer cell. 12-6 LJ-based wall conditions were also imposed at the z boundary during the rescaling process to prevent water molecules crossing the z boundary. 3) The electrolyte cell was built upon the water cell where metal ions were randomly inserted. The number of metal ions was determined according to the concentration of 0.1 M. The electrolyte cell was rescaled to the final density of close to 1 g/cm^3 to imitate the solution density of the mixture of electrolytes. The rescaling process was also performed under the wall constraint as described for the polymer and water cell. The three equilibrated systems were concatenated along the z axis and 21-step equilibration scheme was performed again for the sandwiched model. Upon equilibrium, two carbon sheets were placed at each end side of the sandwiched model with 1 atm external force exerted on them. The whole system was equilibrated again at 300K for 1 ns. The final snapshot from the last equilibration run was used to start the production run of non-equilibrium simulation for 15 ns where an electric field of 0.03 V Å^{-1} was applied on ions. The electric field was removed when ions completely passed the membrane and reached the water reservoir. Half of the carbon atoms of the spirobisindane unit was tethered to their original position via a harmonic spring of 0.1 kcal/mol/ Å^2 throughout the equilibration and non-equilibrium simulation. This is to prevent the drift-away of the membrane while maintaining the reasonable flexibility. The number of water molecules for the salt and water reservoir is 5292 while the number of water molecules within the polymer membrane is 1321. The number of ions is 9 for each cation (K^+, Na^+, Li^+ and Mg^{2+}) and 45 for the anion (Cl^-).

Umbrella sampling was used to compute the free energy of ion transporting within the membrane. The path along the z axis perpendicular to the membrane cross section starting from the inlet interface to the outlet interface was used as the reaction coordinate, specifically from –21 Å to 123 Å. A harmonic spring of 1.5 kcal mol^{-1} Å^{-2} was employed to steer the transmembrane process of ions. At each step, ions were progressively pulled towards the potential centre for 20 ps and then 50 ps production run was used for data acquisition. There are a total of 26 windows. The weighted histogram analysis method (WHAM) algorithm was used to generate the free energy profile[18].

Radial Distribution Functions (RDF)

RDF measures the likeliness of finding a particle at a distance of r away from a reference particle. It describes the interaction between two particles in a local environment. The RDFs of different components in the polymer model are calculated by the built-in $g(r)$ utility in VMD[19]. The formula is provided below:

$$g_{ab}(r) = \frac{1}{N_a N_b} \sum_{i=1}^{N_a} \sum_{j=1}^{N_b} \langle \delta(|r_{ij}| - r) \rangle \tag{7}$$

where N_a/N_b is the number of particles of atom type a and b respectively; r_{ij} is the radial distance between particle i of type a and particle j of type b; $< \cdots >$ is the ensemble average.

Radial number density distribution is also calculated for a direct comparison between different polymer systems. The formula is provided below:

$$n_{ab}(r) = \rho g_{ab}(r) \tag{8}$$

where ρ is the number density of the atom species of interest.

Coordination Number

Coordination number (CN) describes the number of particles within a certain distance from the reference particle. It is particularly useful to quantify the solvation structure of ions in an aqueous environment. CN can be derived from the RDF by integrating $g(r)$ over a defined range of distances. The formula isprovided below:

$$CN = 4\pi\rho \int_0^R g(r) r^2 dr \quad (9)$$

where ρ is the number density of water molecules, $g(r)$ is the RDF between metal ions and water molecules, R is the defined upper limit of distances.

Binding energy

Binding energies of electrolyte ions with the pristine amidoxime group were determined to indicate the affinity between ions and functional groups. The binding energy was calculated by comparing the single-point energy of the monomer, ion, and monomer-ion system, denoted as E_{mono}, E_{ion}, and $E_{\text{mono-ion}}$ respectively. As such, the binding energy is derived using the following formula: $E_{\text{bind}} = E_{\text{mono-ion}} - (E_{\text{mono}} + E_{\text{ion}})$. The energies of respective systems were computed using the DFT method. The monomer structure of the pristine AO-PIM-1 was optimised using B3LYP functional with 6-31G(d,p) basis set within the SMD solvation model with water as the implicit solvent. The frequency calculation was also performed to guarantee a true minimum. The relevant calculations employed the default convergence criteria including the Maximum Force of 4.5×10^{-4} Hartree/Bohr, the Root Mean Square (RMS) Force of 3.0×10^{-4} Hartree/Bohr, the Maximum Displacement of 1.8×10^{-3} Bohr, and the RMS Displacement of 1.2×10^{-3} Bohr. The single-point energy of ions was computed using the same setting. To obtain the complex structure of monomer-ion, a single ion was manually placed close to the amidoxime group to mirror the binding situation. The same geometry optimization and frequency calculation were performed for the complex structure. All DFT calculations were implemented in Gaussian 16 [20].

Pore analysis

Pore analysis was conducted using the Zeo++ package[21]. Pore size distributions were measured using 1 Å probe with 70000 Monte Carlo (MC) samples. Pore size distributions of membranes at the hydrated state were also computed with water molecules removed from the system before implementing Zeo++.

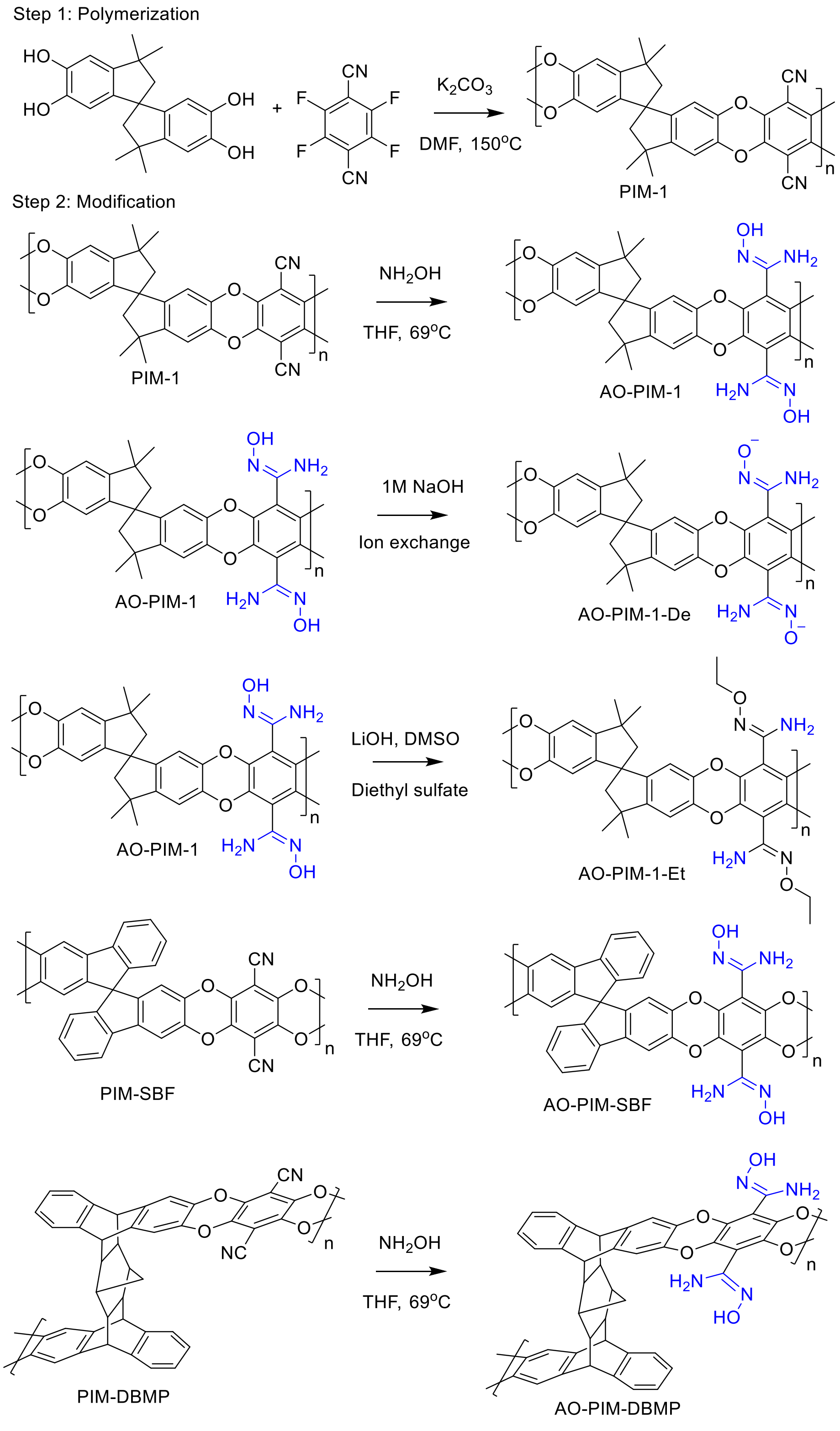

Supplementary Figure S1. Synthetic route of PIM-1 polymer and post synthetic modification to hydrophilic PIM polymers with amidoxime groups. Modifications of PIM-SBF and PIM-DBMP are also included.

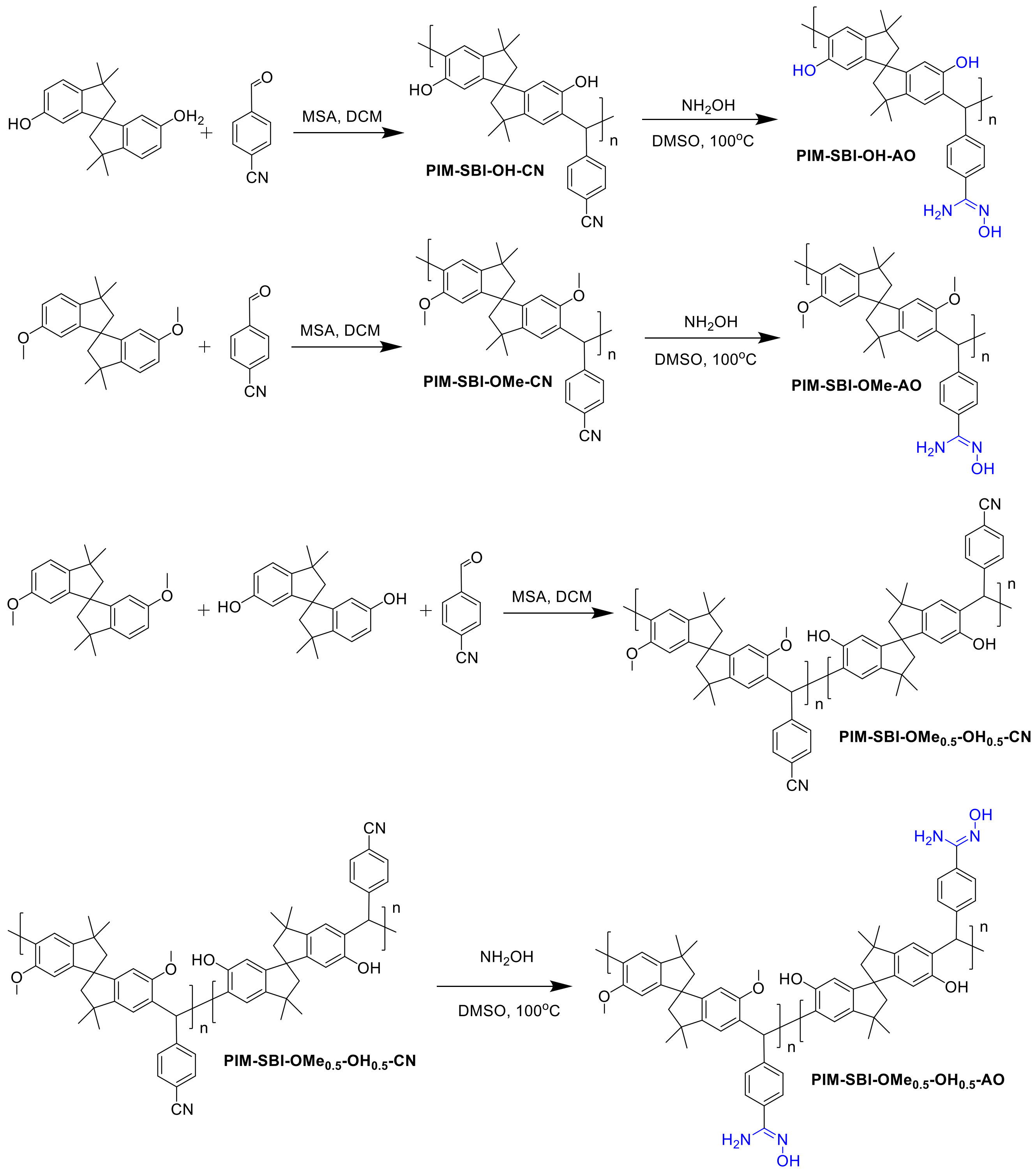

Supplementary Figure S2. Synthetic route of ether-free PIM polymers and post-synthetic modification to hydrophilic AO-PIM polymers.

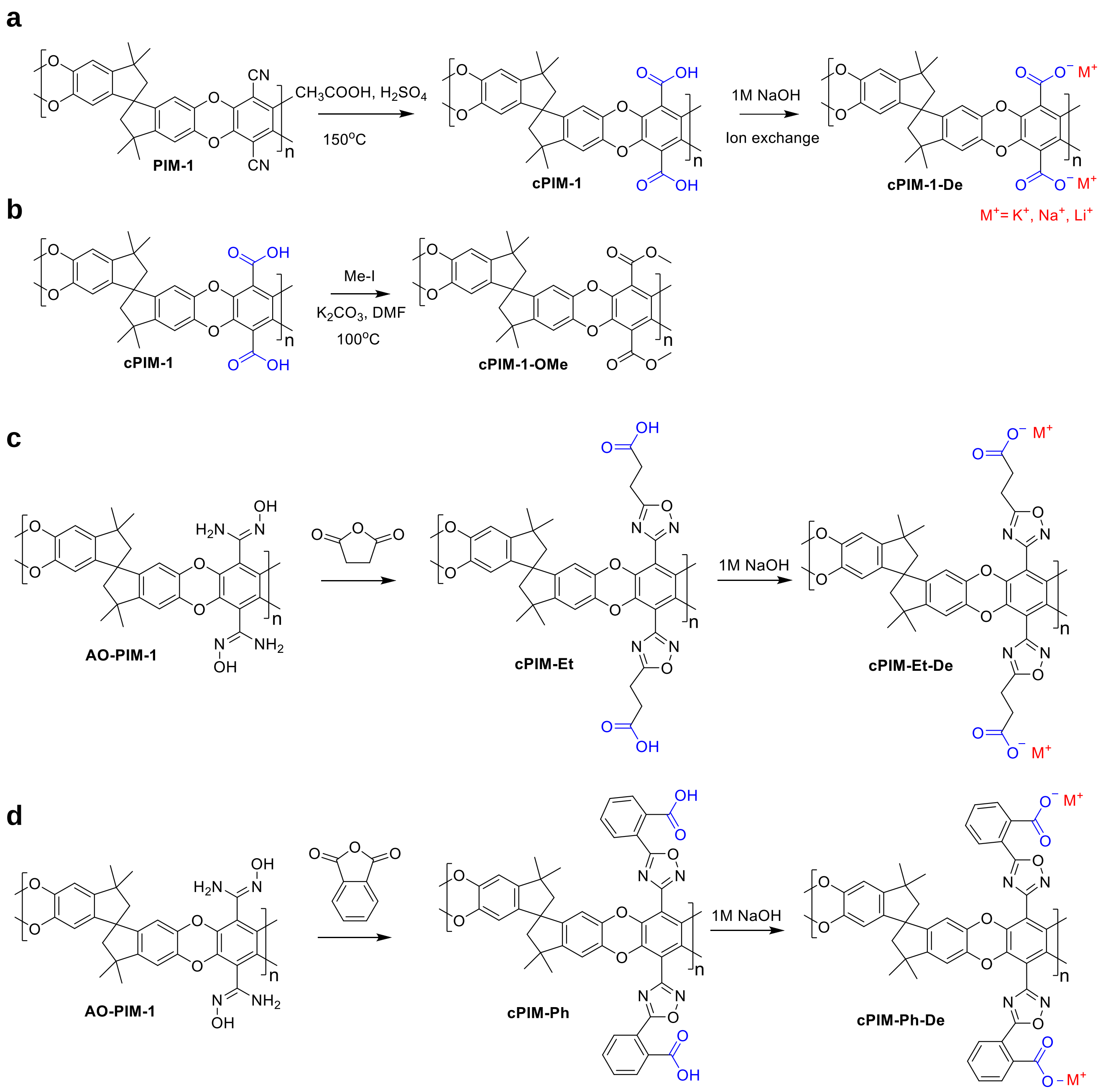

Supplementary Figure S3. Synthetic route of PIM polymers with carboxylic acid and modifications.

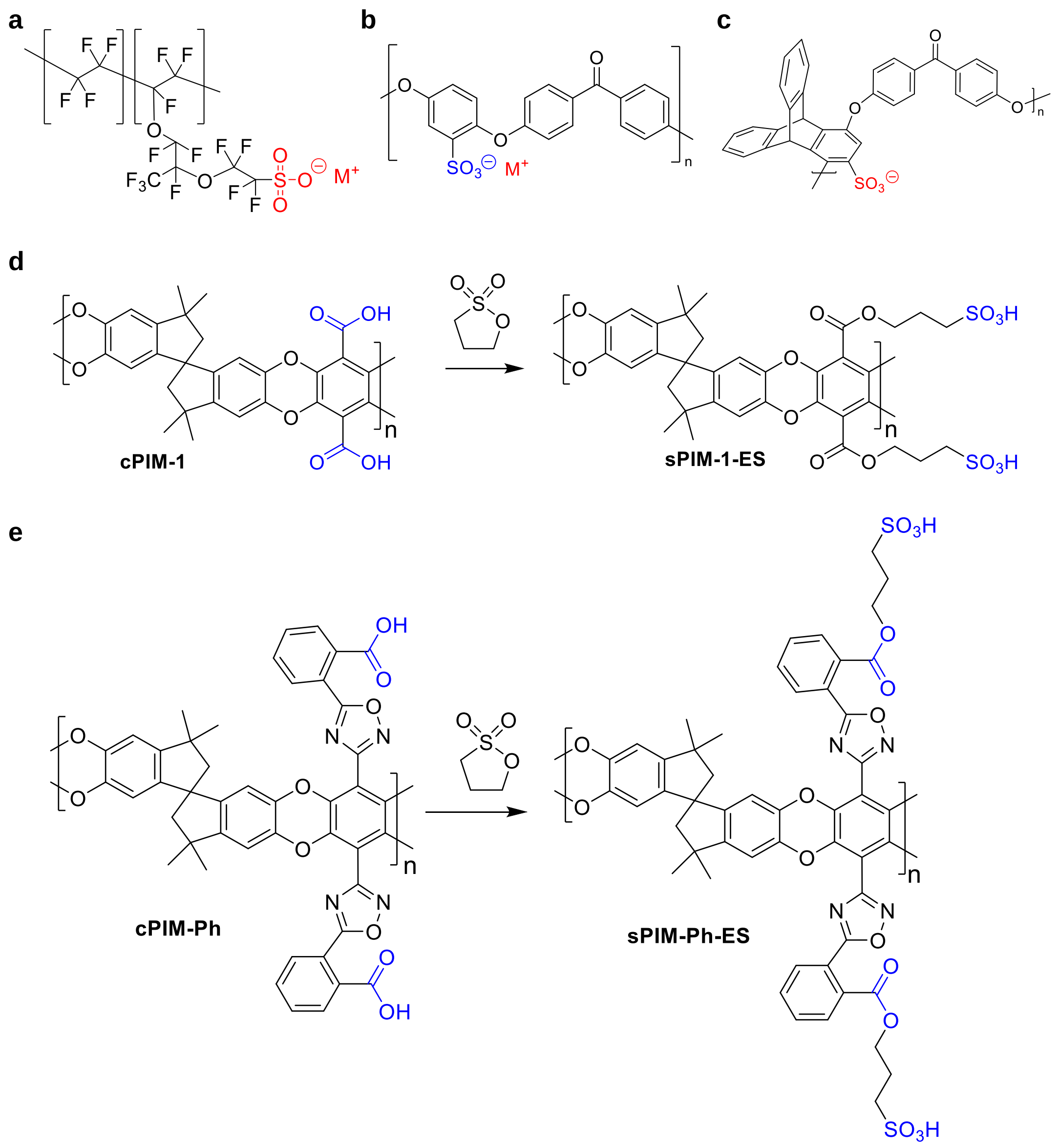

Supplementary Figure S4. Sulfonated polymer membranes and synthesis of sulfonated PIM polymers.

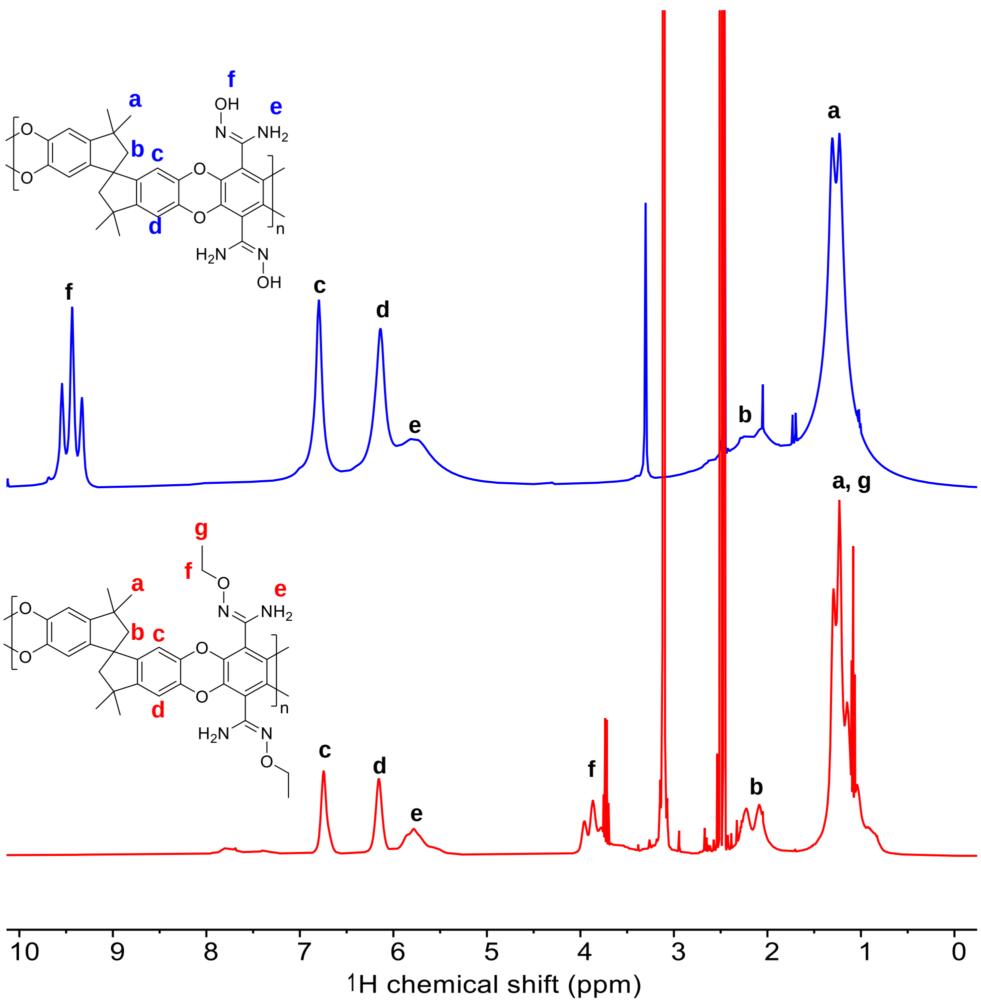

Supplementary Figure S5. ^{1}H NMR spectra of AO-PIM polymers.

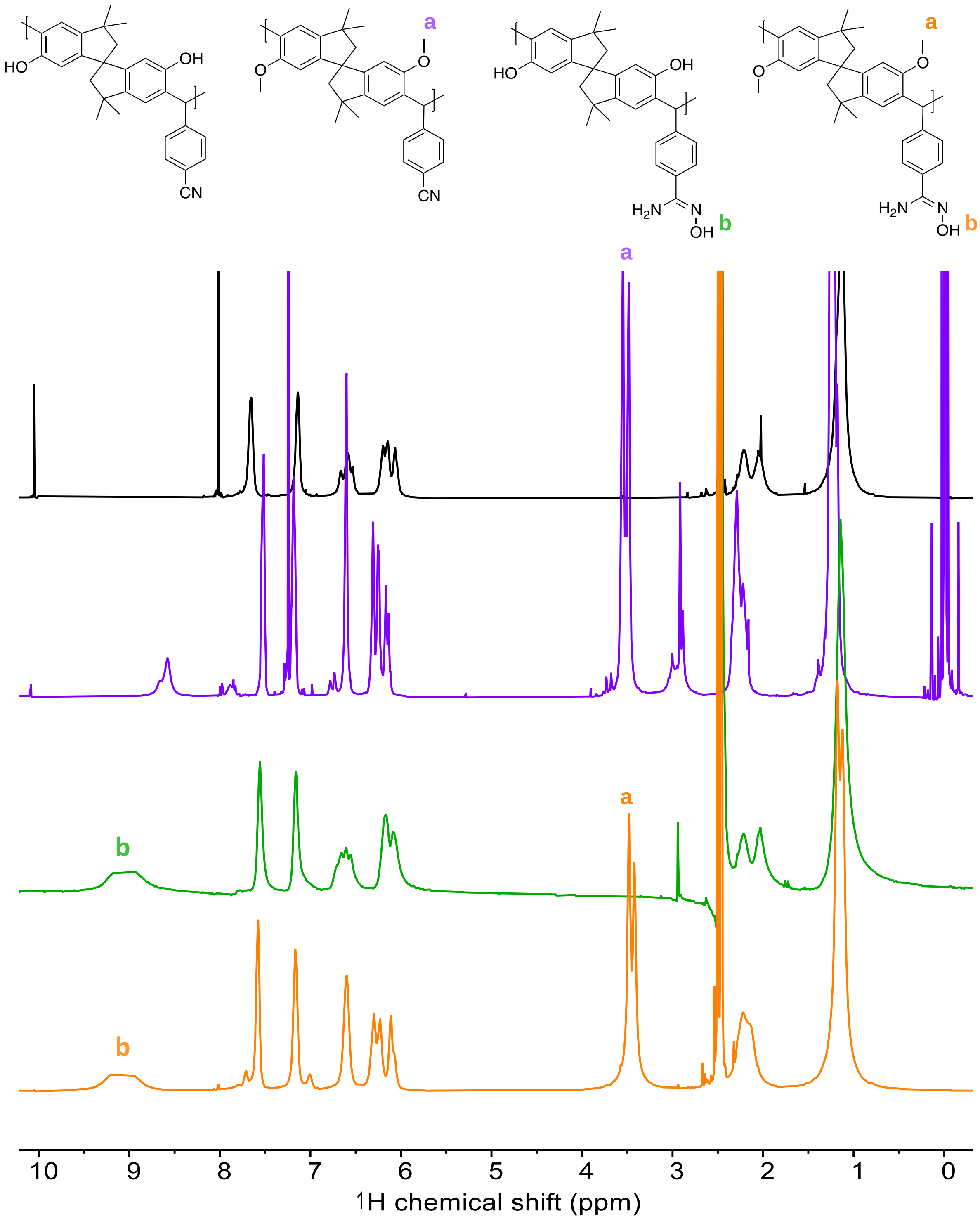

Supplementary Figure S6. ^{1}H NMR spectra of ether-free PIM polymers.

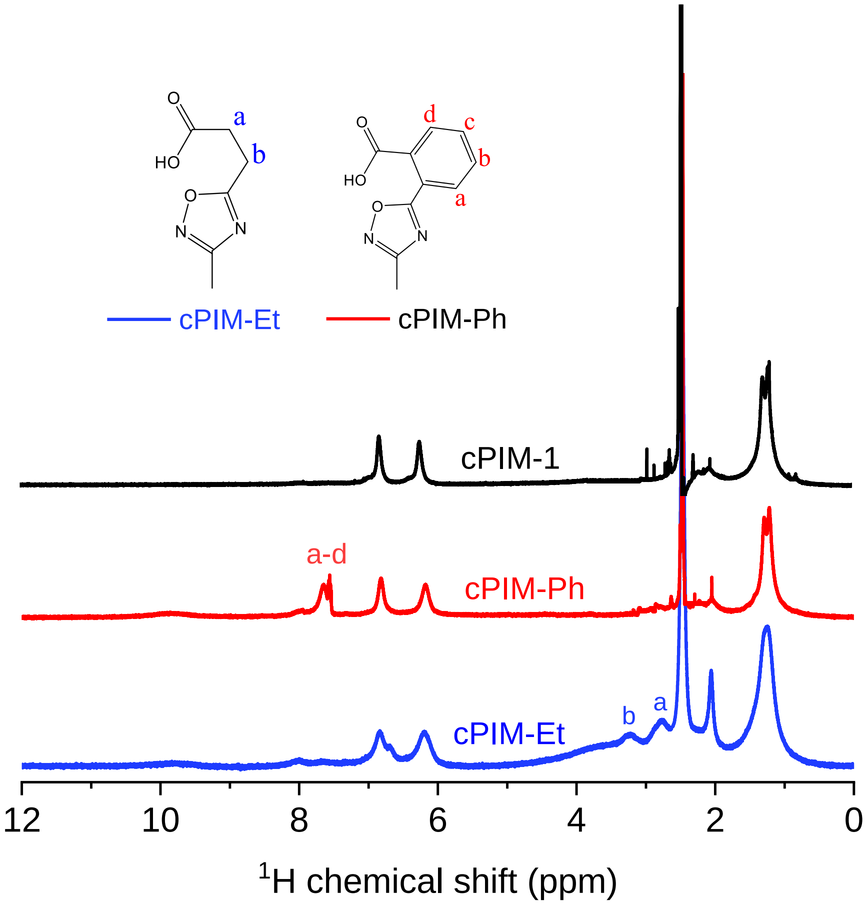

Supplementary Figure S7. ^{1}H NMR spectra of PIM polymers with carboxylic acid groups.

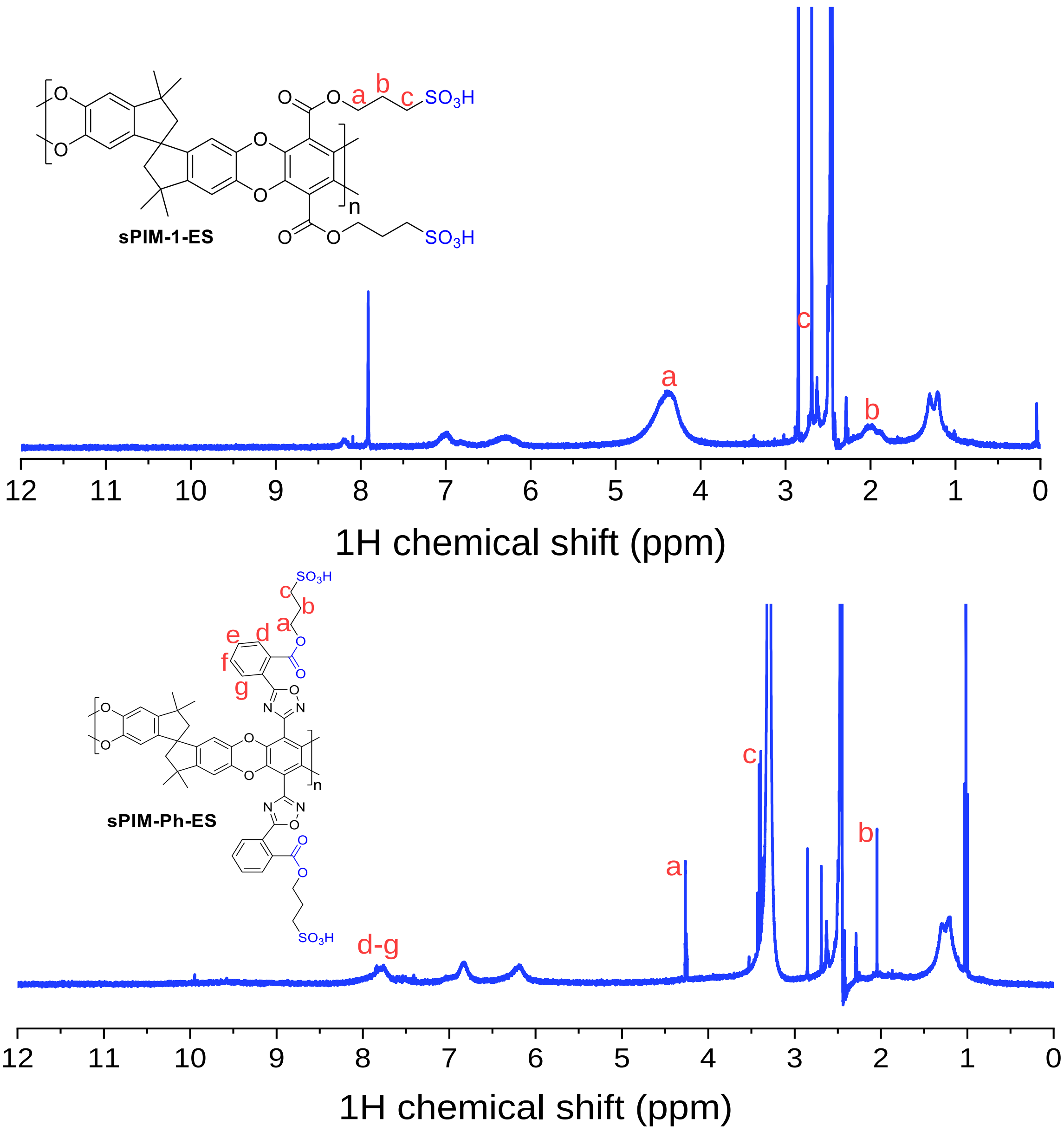

Supplementary Figure S8. ^{1}H NMR spectra of PIM polymers with sulfonic acid groups.

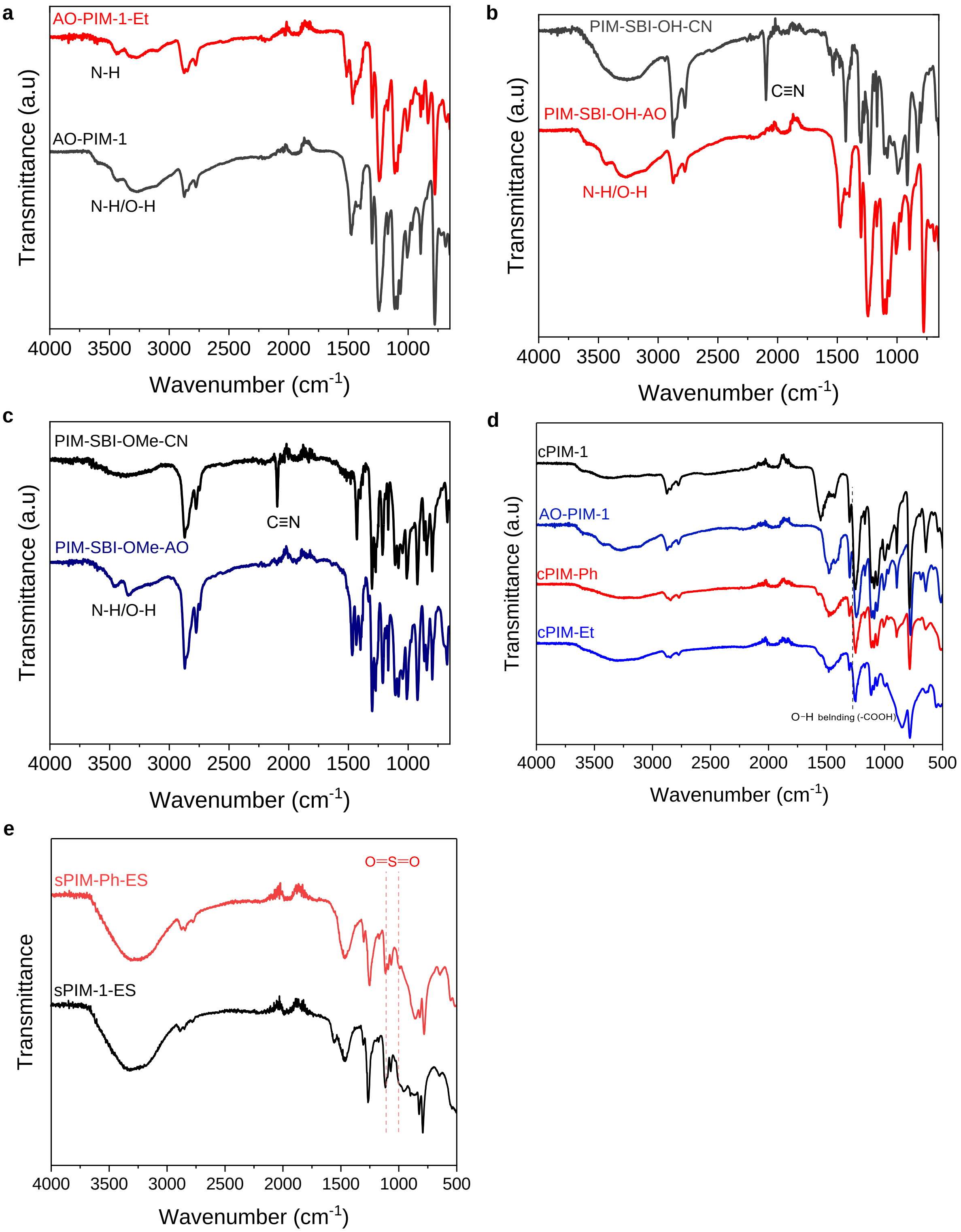

Supplementary Figure S9. FTIR spectra of polymers. **a**, FTIR spectra of AO-PIM-1 and AO-PIM-1-Et. **b**, FTIR spectra of PIM-SBI-OH-CN and PIM-SBI-OH-AO. **c**, FTIR spectra of PIM-SBI-OMe-CN and PIM-SBI-OMe-AO. **d**, FTIR spectra of AO-PIM-1, cPIM-1, cPIM-Et and cPIM-Ph. **e**, FTIR spectra of sPIM-1-ES and sPIM-Ph-ES.

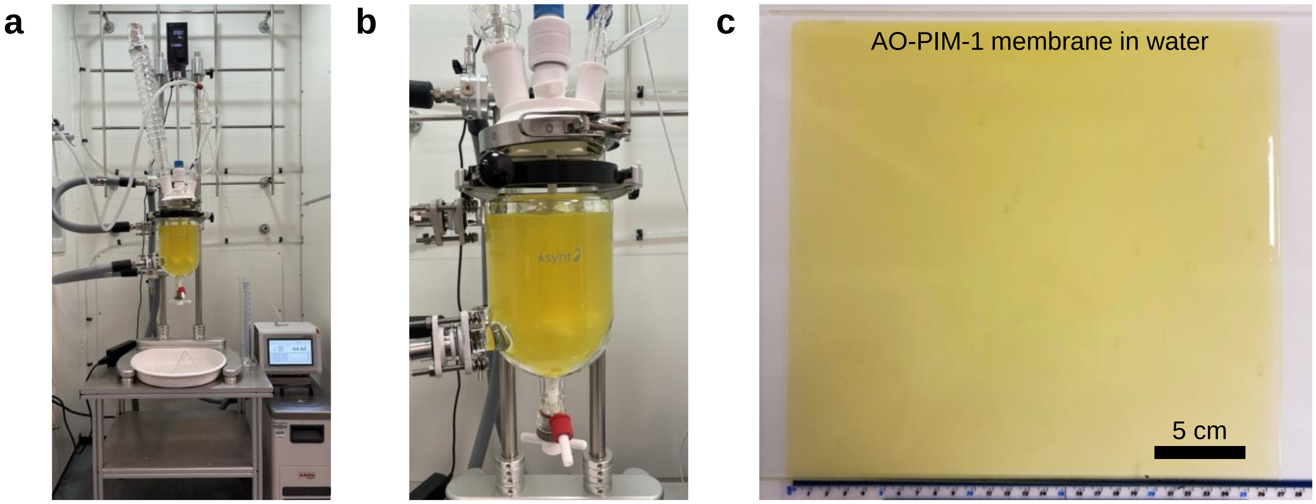

Supplementary Figure S10. Upscaling of PIM synthesis and membranes. a, b, Photos of polymer synthesis and **c**, Photo of AO-PIM-1 membranes in water bath.

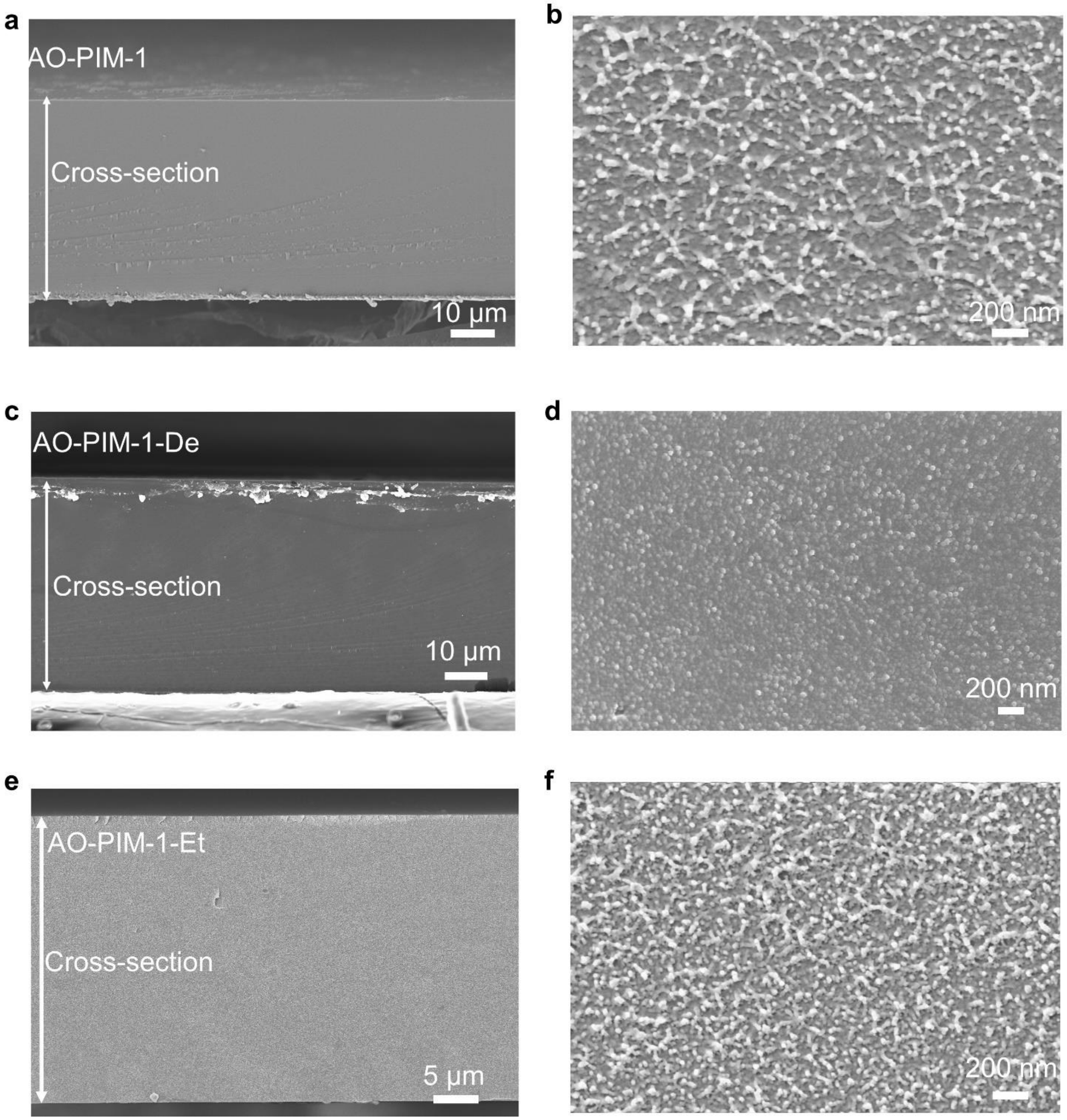

Supplementary Figure S11. Cross-sectional SEM images of membranes derived from PIM-1. (**a-b**) AO-PIM-1. (**c-d**) AO-PIM-1-De. (**e-f**) AO-PIM-1-Et. The membranes are dense without phase separation.

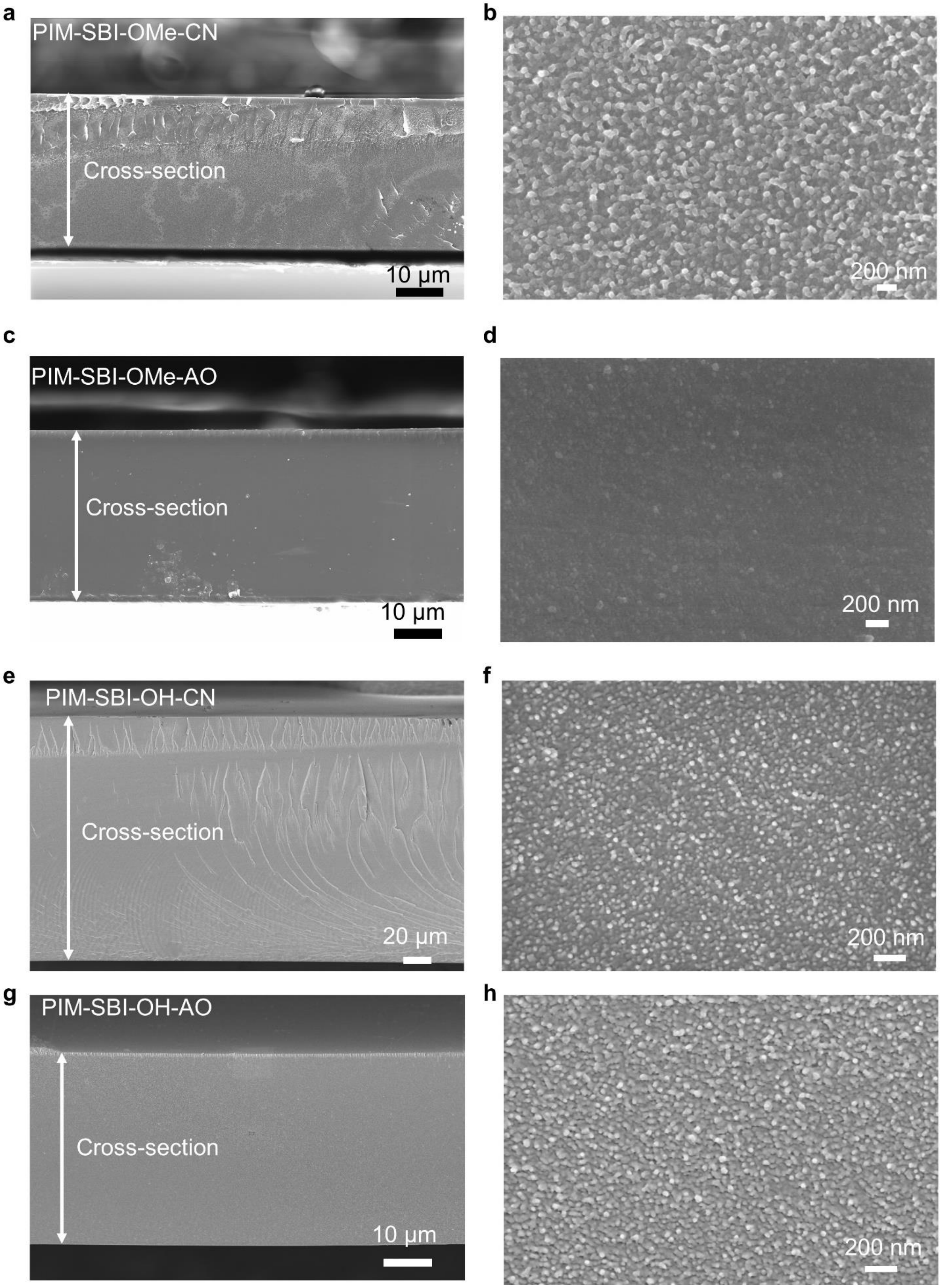

Supplementary Figure S12. Cross-sectional SEM images of ether-free PIM membranes. (**a-b**) PIM-SBI-OMe-CN. (**c-d**) PIM-SBI-OMe-AO. (**e-f**) PIM-SBI-OH-CN. (**g-h**) PIM-SBI-OH-AO.

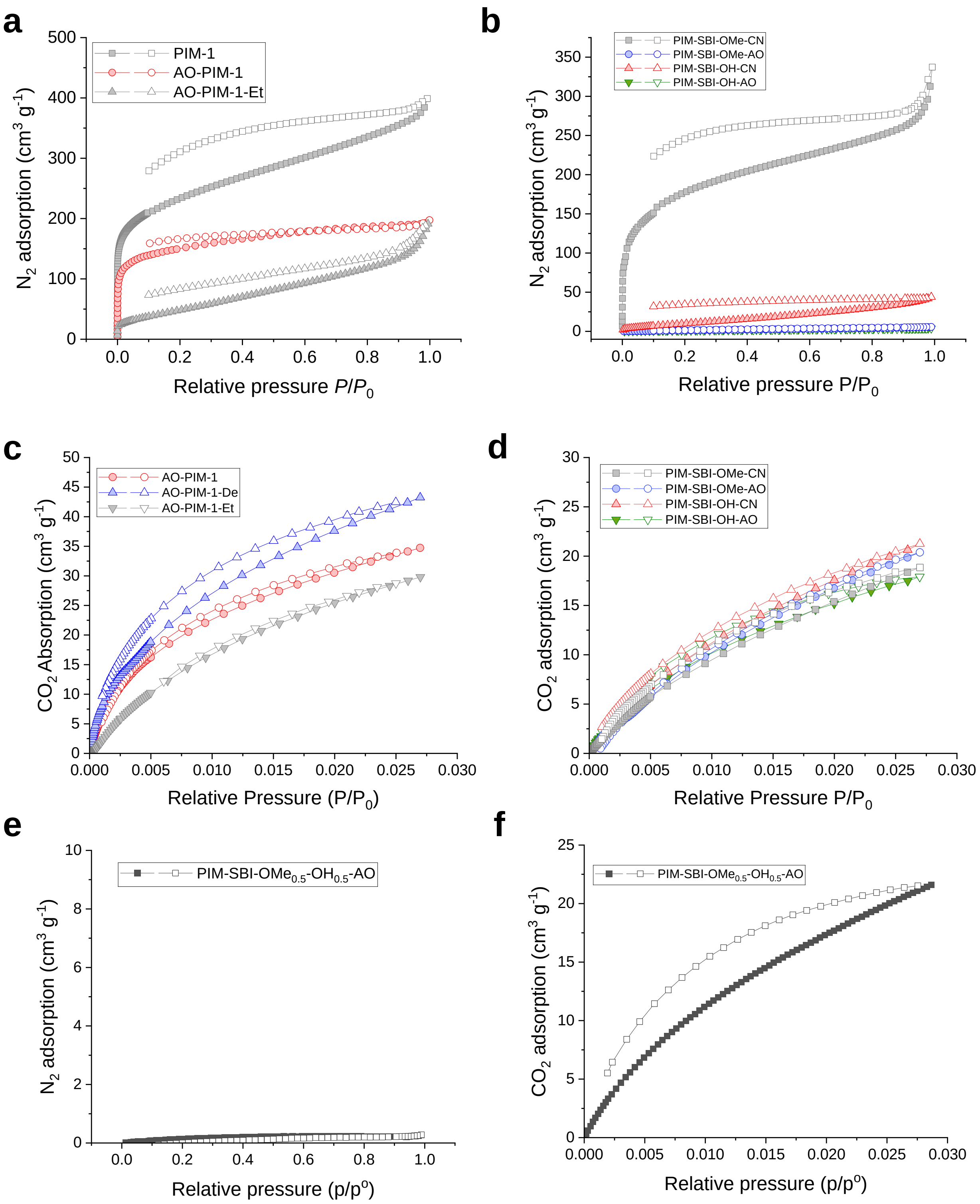

Supplementary Figure S13. N_2 and CO_2 adsorption isotherms. (**a-b**) N_2 adsorption at 77K. (**c-d**) CO_2 adsorption at 273K. (**e-f**) N_2 adsorption at 77K and CO_2 adsorption at 273K for PIM-SBI-OMe$_{0.5}$-OH$_{0.5}$-AO. For modified polymers with hydrophilic groups, the low N_2 adsorption is due to restricted kinetic diffusion of N_2 in the rigid hydrogen-bonded network, which has been reported in the literature, such as carboxylate PIM polymers. CO_2 adsorption isotherms show relatively low CO_2 adsorption capacity in ether-free PIM polymers (panels d and f), suggesting the presence of ultramicropores.

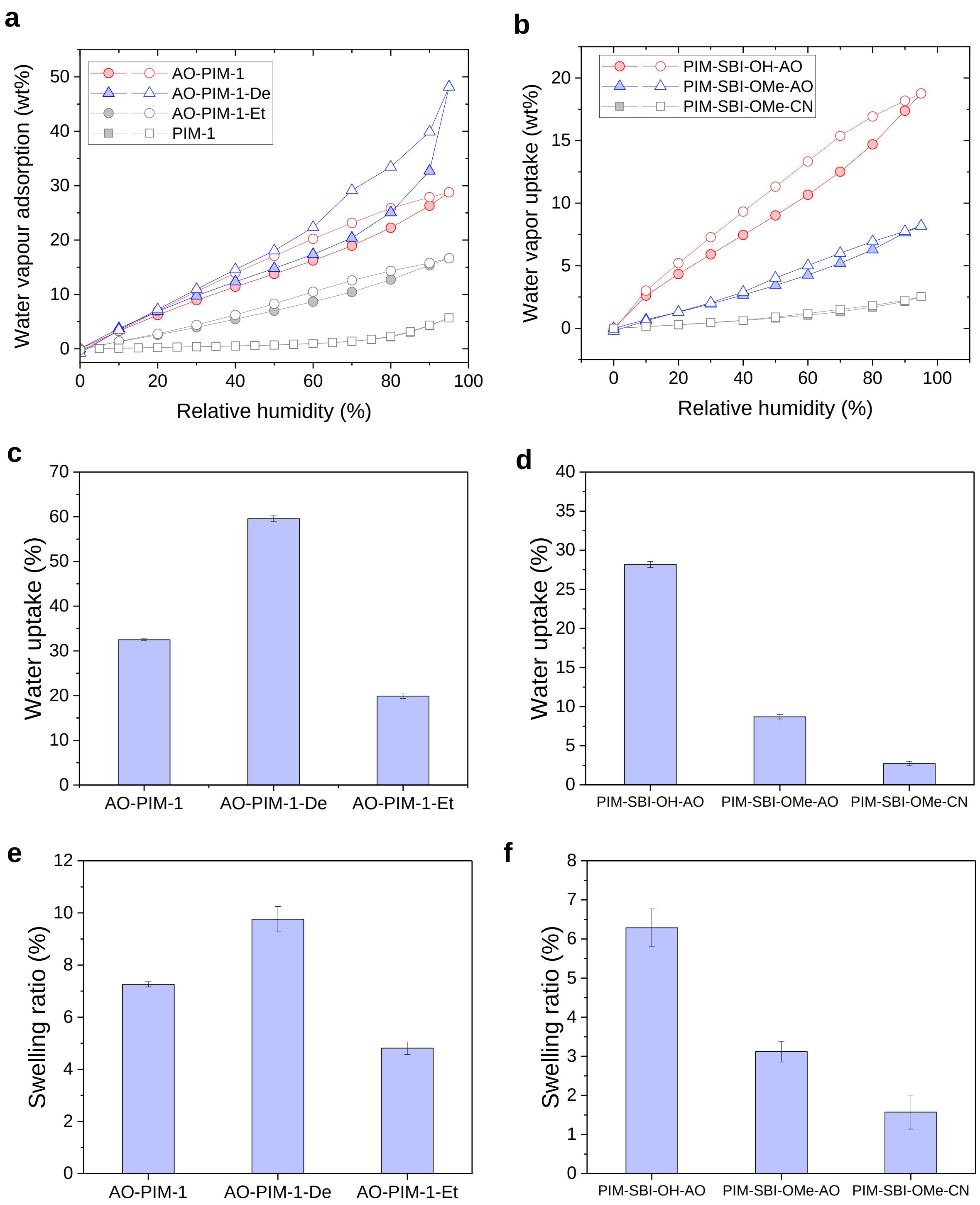

Supplementary Figure S14. Water uptake and swelling ratio of membranes. a, Water vapour uptake profiles of PIM-1, AO-PIM-1, AO-PIM-1-De and AO-PIM-1-Et. **b**, Water vapor uptake of PIM-SBI-OH-AO, PIM-SBI-OMe-AO and PIM-SBI-OMe-CN. **c**, Water uptake of AO-PIM-1, AO-PIM-1-De and AO-PIM-1-Et. **d**, Water uptake of PIM-SBI-OH-AO, PIM-SBI-OMe-AO and PIM-SBI-OMe-CN. **e**, Swelling ratio of AO-PIM-1, AO-PIM-1-De and AO-PIM-1-Et. **f**, Swelling ratio of PIM-SBI-OH-AO, PIM-SBI-OMe-AO and PIM-SBI-OMe-CN. In **c-f**, the error bars represent the standard deviation (s.d.); the data are presented as the mean ± s.d. (n=4).

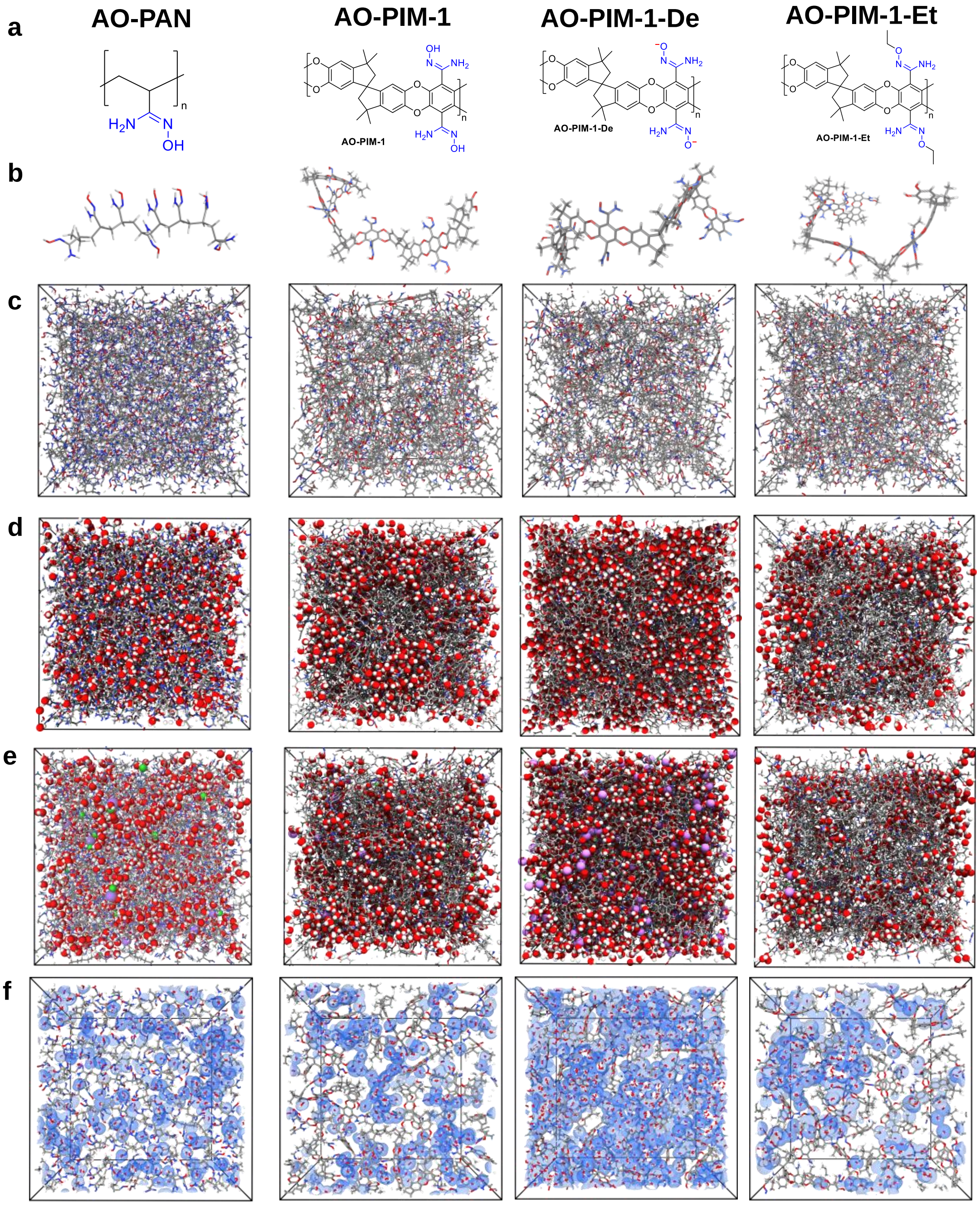

Supplementary Figure S15. Modelling of polymer membranes. a, Chemical structures of polymers. **b**, Models of polymer chain segments. **c**, Polymer models in solid state. The size of amorphous cells are: AO-PAN (50.2 Å), AO-PIM-1 (49.5 Å), AO-PIM-1-De (49.2 Å), and AO-PIM-1-Et (52.0 Å). **d**, Models of hydrated polymers with water adsorption similar to experimentally measured values. The size of amorphous cells are: AO-PAN (53.7 Å), AO-PIM-1 (52.0 Å), AO-PIM-1-De (55.9 Å), and AO-PIM-1-Et (53.8 Å). **e**, Models of hydrated polymers with 1M LiCl salt ions loaded in the cell. Color of atoms and ions: red - oxygen; blue – nitrogen; grey – carbon; white – hydrogen; purple - lithium ions. The size of amorphous cells are: AO-PAN (53.8 Å), AO-PIM-1 (52.3 Å), AO-PIM-1-De (56.5 Å), and AO-PIM-1-Et (53.7 Å). **f**, Cross-sections of amorphous cells of hydrated polymers, 10 Å in thickness. Polymer chains are shown in line-and-stick representation where grey, red, navy and white represent carbon, oxygen, nitrogen and hydrogen, respectively. Light blue shading highlights the isosurface of water to visualize water clusters.

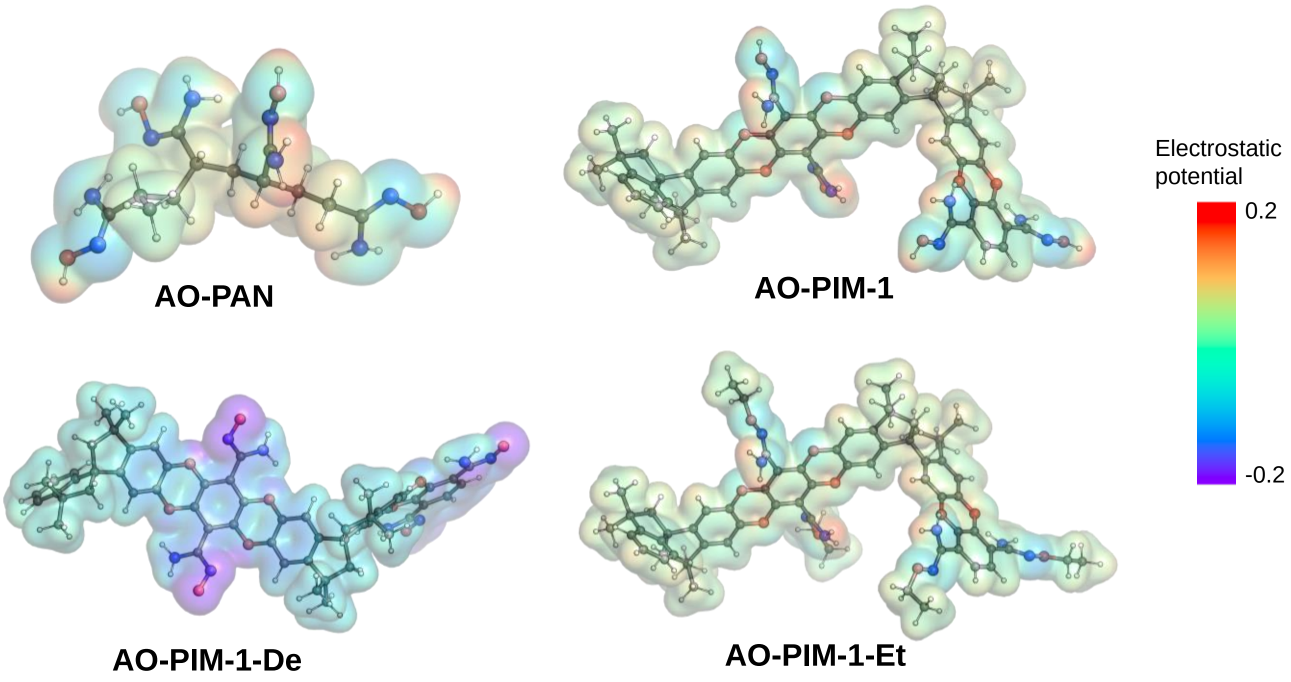

Supplementary Figure S16. Electrostatic potential (ESP) of polymer chain repeating units.

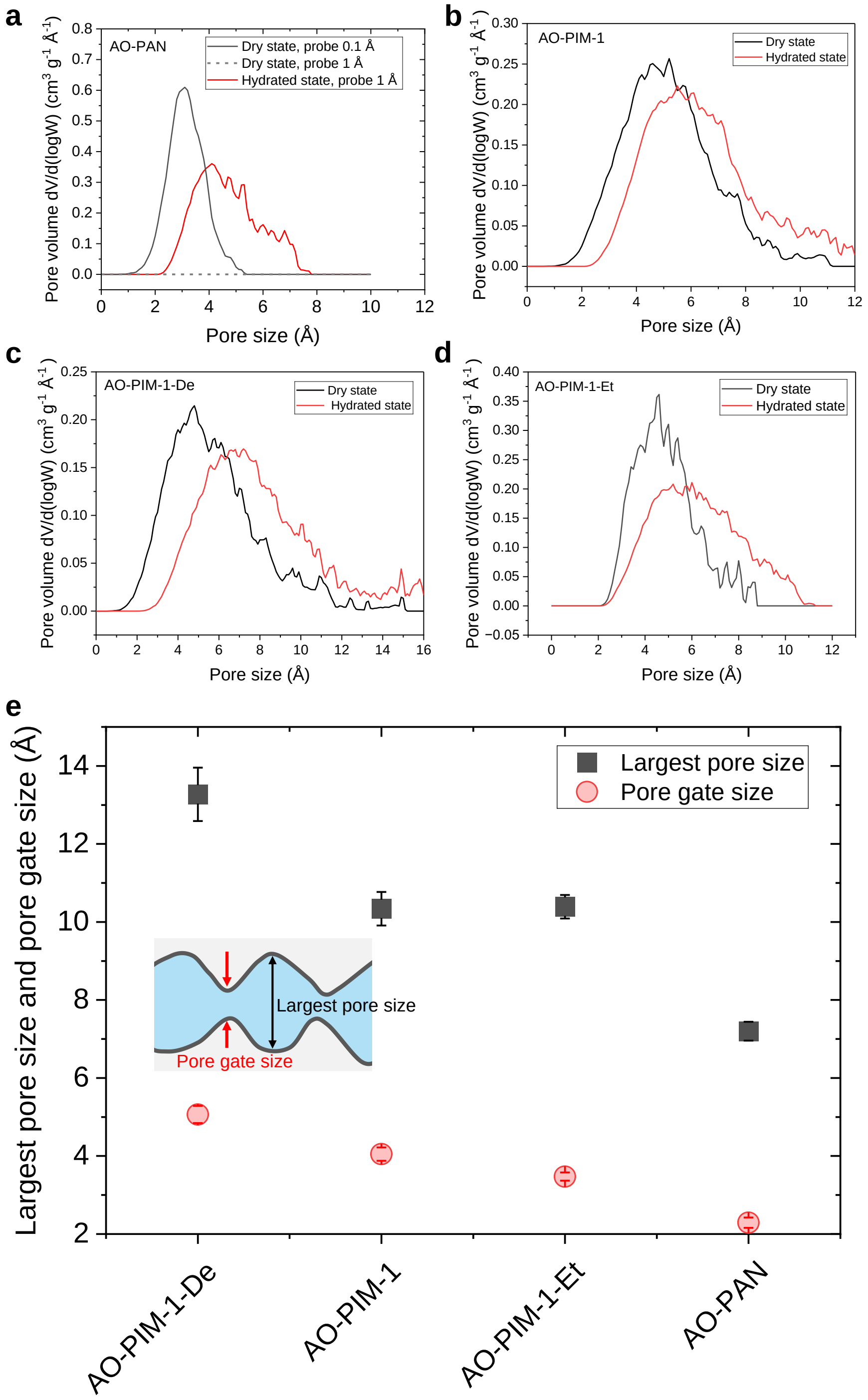

Supplementary Figure S17. Pore size distributions derived from computational dry and hydrated polymer models. a, AO-PAN at dry and hydrated states. **b**, AO-PIM-1 at dry and hydrated states. **c**, AO-PIM-1-De at dry and hydrated states. **d**, AO-PIM-1-Et at dry and hydrated states. **e**, Statistical analysis of average largest pore size and pore gate size, according to the hour-glass model (shown in the inset). In **e**, the error bars represent the standard deviation (s.d.); the data are presented as the mean ± s.d. (n=10).

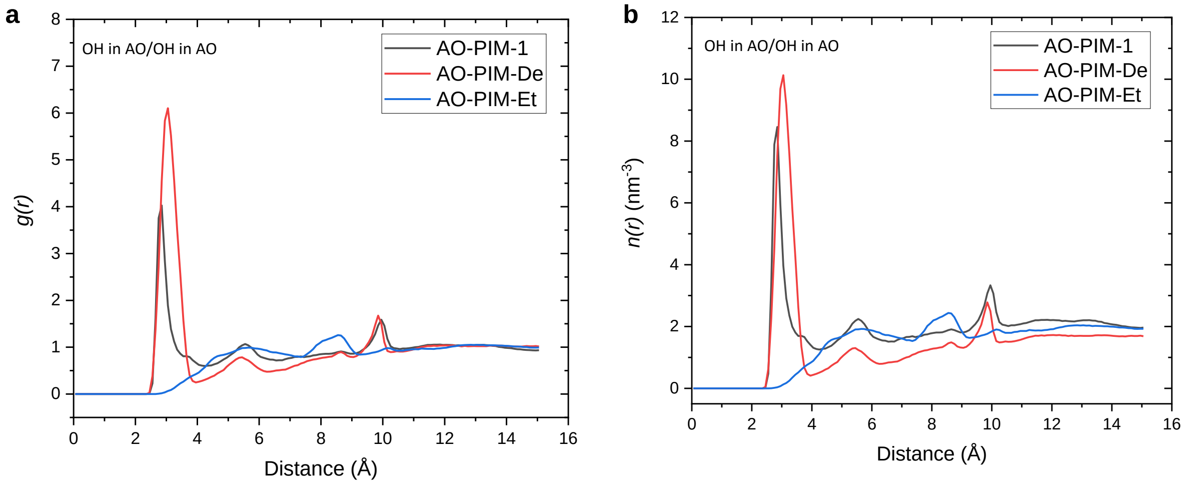

Supplementary Figure S18. Radial distribution functions derived from hydrated polymer models. **a**, RDF as a function of the distance between oxygen atoms in amidoxime groups. **b**, Radial number density distribution function as a function of the distance between oxygen atoms in amidoxime groups. The results suggest strong interactions between amidoxime groups via hydrogen bonding in AO-PIM-1. In contrast, the distance between negatively charged AO groups in AO-PIM-1-De suggests electrostatic repulsions. In AO-PIM-1-Et, the hydrogen bonding between hydroxyl groups is not observed, though strong interactions between amine groups might occur (not calculated here).

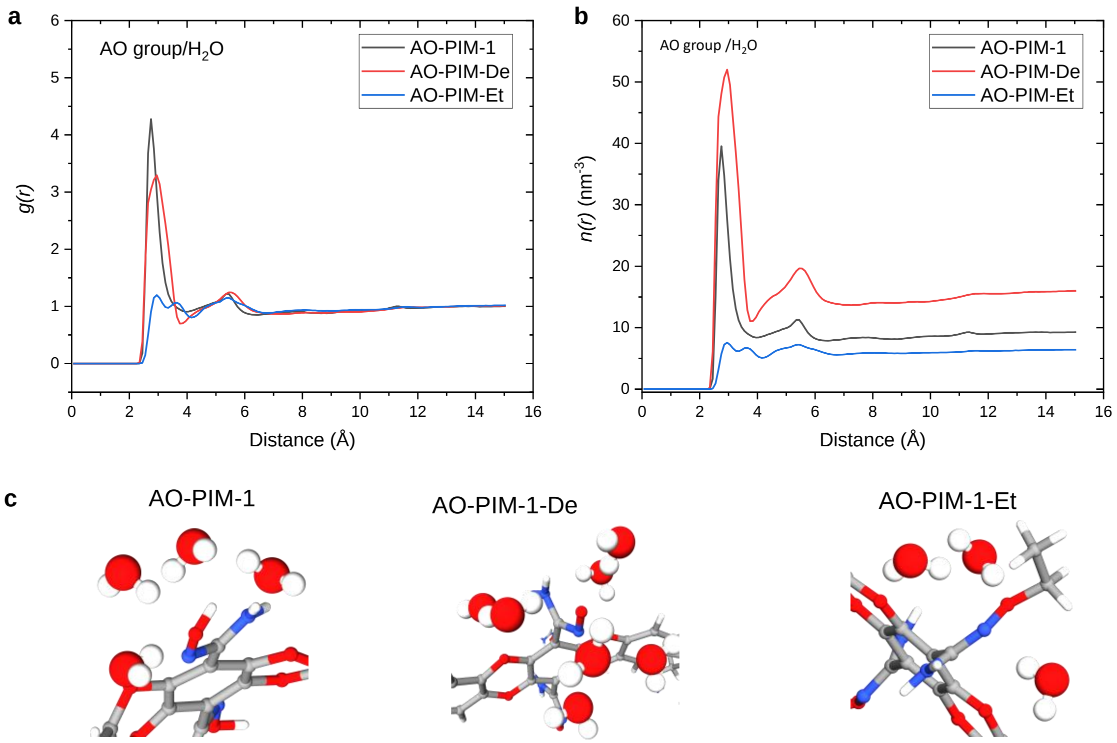

Supplementary Figure S19. Comparison of radial distribution functions. a, RDF as a function of the distance between oxygen atoms in amidoxime groups and surrounding water molecules. **b**, Radial number density distribution functions as a function of the distance between oxygen atoms in amidoxime groups and surrounding water molecules. **c**, Models showing the comparison of the number of water molecules located within the first hydration shell of functional groups.

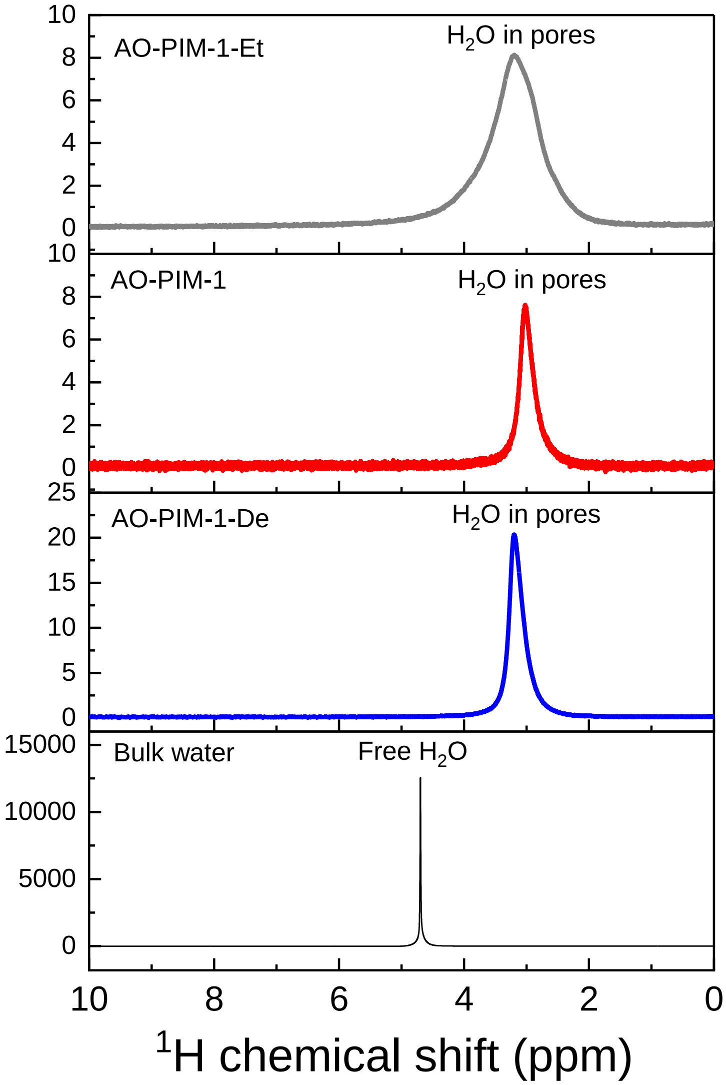

Supplementary Figure S20. ^{1}H NMR spectra of bulk water and water confined in micropores in PIM membranes. The distinct peaks centered at 3 ppm in the ^{1}H ssNMR spectra corresponds to water confined in micropores in AO-PIM-1 polymer membranes, consistent with our previous study[22]. In AO-PIM-1-De, the peak became sharp and moved towards higher chemical shifts due to the higher water uptake. In AO-PIM-1-Et, the peak broadening is due to restricted motion in the micropores.

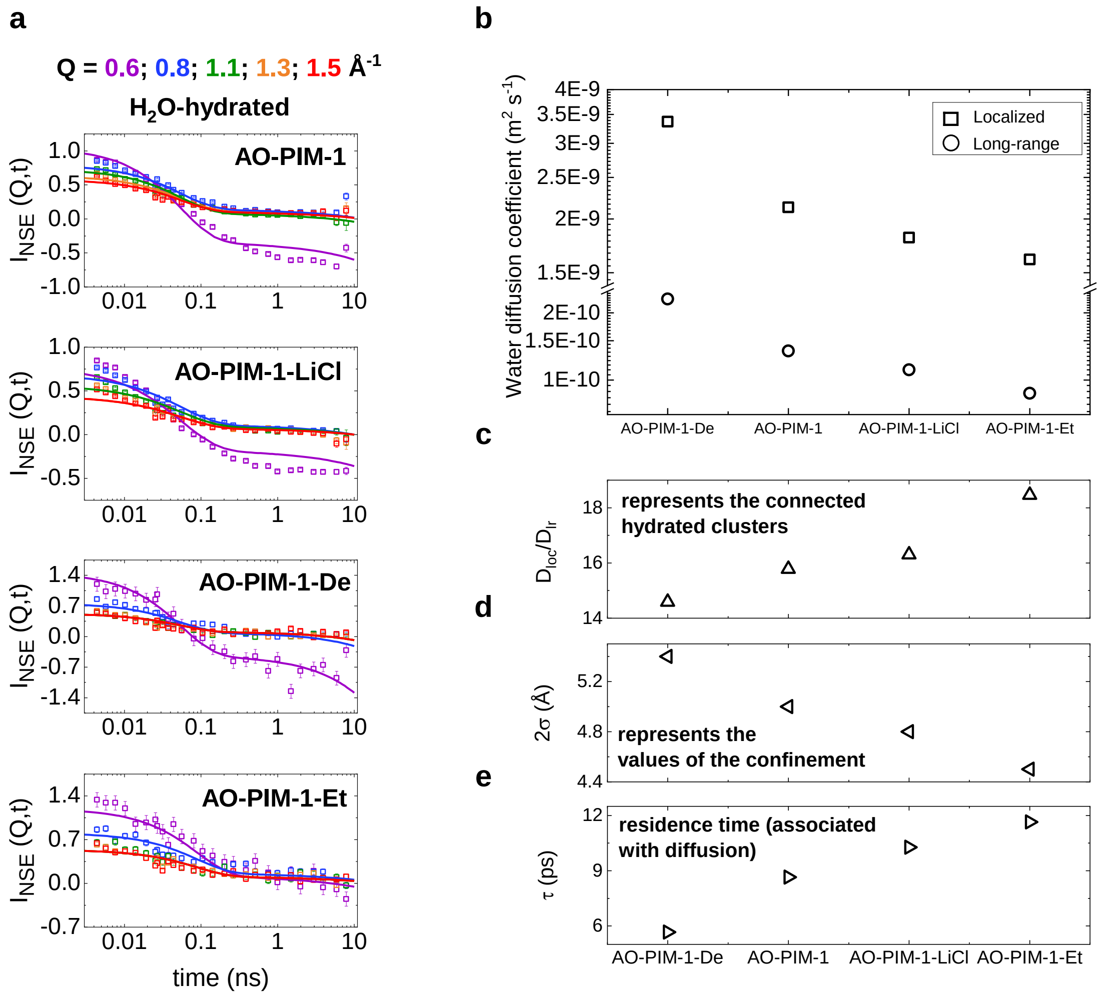

Supplementary Figure S21. Neutron Spin Echo profiles and data analysis. **a**, Water dynamics *Q*-dependency of *I(Q,t)* over the time range of τ = 4 ps to 8 ns, covering momentum transfers from Q = 0.6 to 1.6 Å^{-1}. **b**, Localized and long-range water self-diffusion coefficient derived from the neutron scattering measurements. **c**, Ratio of localized diffusion over long-range diffusion, D_{loc}/D_{lr}. **d**, Confinement size; **e**, Residence time.

QENS is a powerful technique that enables quantitative analysis of molecular dynamics on the nano- to picosecond scale, both in bulk and within confined geometries. As such, it has been widely used to study water mobility in various membranes[7-9]. In nanoconfined systems, modeling the intermediate scattering function, *I(Q,t)*, with the Gaussian model allows for the separation of dynamics within pores or nanodroplets (D_{loc}) and across them (D_{lr}), as well as for the characterization of key parameters related to these dynamics, such as residence time (τ) and confinement size (2σ)[8]. Furthermore, since the local diffusion coefficient reflects molecular motion within confined regions, while the long-range diffusion coefficient provides insights into diffusion on the nanometer scale, their ratio (D_{loc}/D_{lr}) indicates the likelihood of a molecule diffusing from one confined domain to another. This ratio thus reflects the degree of connectivity among hydrated clusters, as observed previously in Nafion and other membranes[7-9].

Analysis of the diffusion coefficient suggests a substantial reduction compared to bulk water, which is not surprising given the level of water uptake in these membranes (below 60 wt%). While the analysis of D_{loc}/D_{lr} implies a certain level of connectivity across the entire set of samples analyzed, it suggests

that AO-PIM-1-De has a higher degree of interconnected water clusters, a conclusion further supported by trends observed in residence time. Interestingly, the analysis of 2σ closely matches the values for pore gate size, though it is slightly higher than the calculated values. Together, these results align with our other experimental and modeling data, reinforcing our overall conclusions. The data presented in **a** represent the scattering profile of the sample acquired at specific Q values (Q = momentum transfer). Specifically, we presented a few Q-values: 0.6, 0.8, 1.1, 1.3, and 1.5 $Å^{-1}$, which are shown in purple, blue, green, orange, and red, respectively. The error bar on the raw data represents statistical error, which is related to the acquisition time, which is related to the acquisition time. The longer the scattering profile requires, the smaller the error becomes.

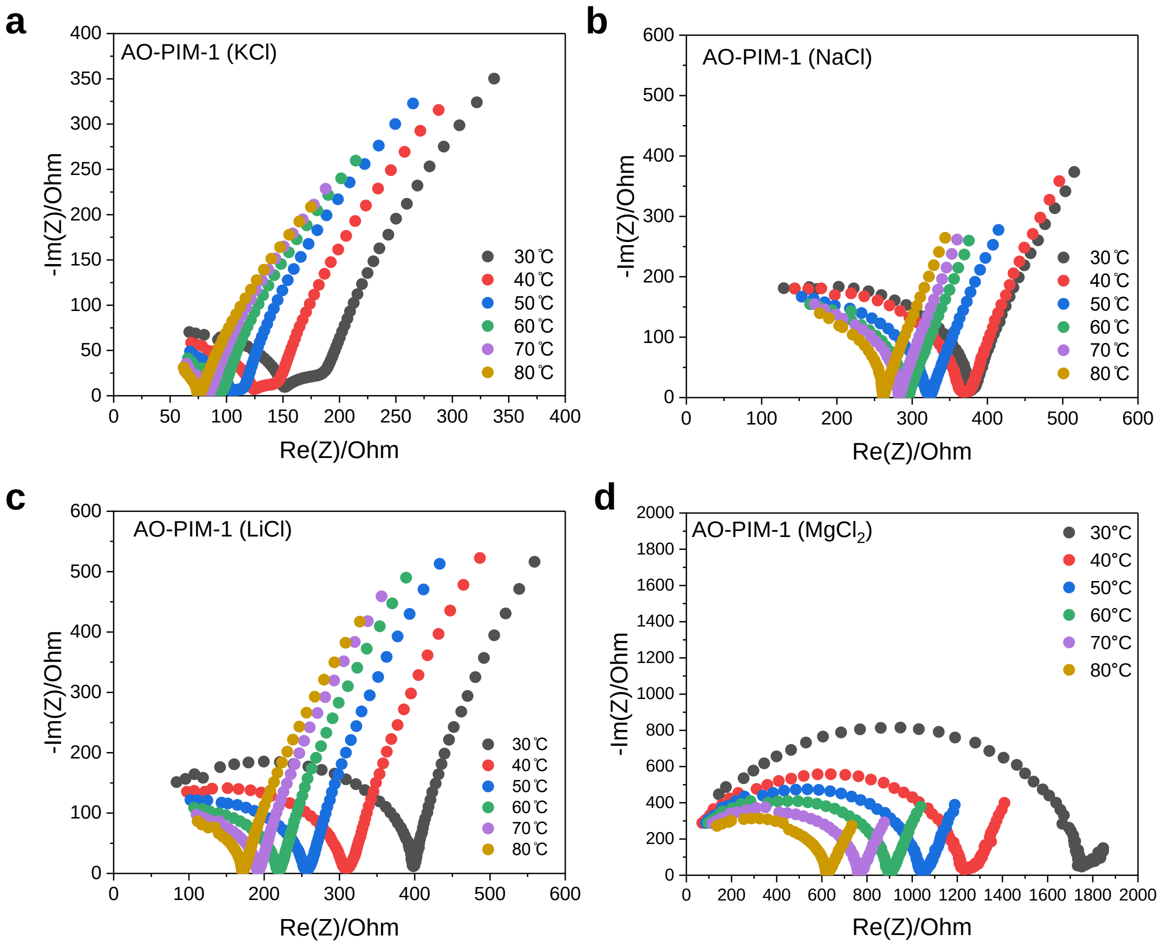

Supplementary Figure S22. Nyquist plots of AO-PIM-1 under different temperatures. a, AO-PIM-1 in KCl solution under different temperatures. **b**, AO-PIM-1 in NaCl solution under different temperatures. **c**, AO-PIM-1 in LiCl solution under different temperatures. **d**, AO-PIM-1 in $MgCl_2$ solution under different temperatures. Concentrations of all salt solutions are 0.1 M.

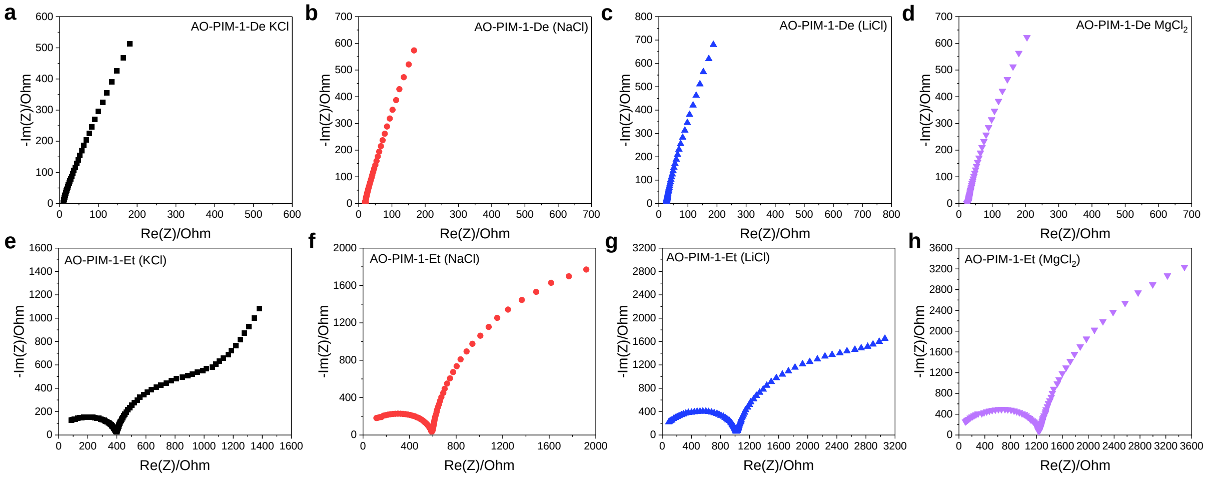

Supplementary Figure S23. Nyquist plots of AO-PIM-1-De and AO-PIM-1-Et. a, AO-PIM-1-De in KCl solution. **b**, AO-PIM-1-De in NaCl solution. **c**, AO-PIM-1-De in LiCl solution. **d**, AO-PIM-1-De in $MgCl_2$ solution. **e**, AO-PIM-1-Et in KCl solution. **f**, AO-PIM-1-Et in NaCl solution. **g**, AO-PIM-1-Et in LiCl solution. **h**, AO-PIM-1-Et in $MgCl_2$ solution. Concentrations of all salt solutions are 0.1 M.

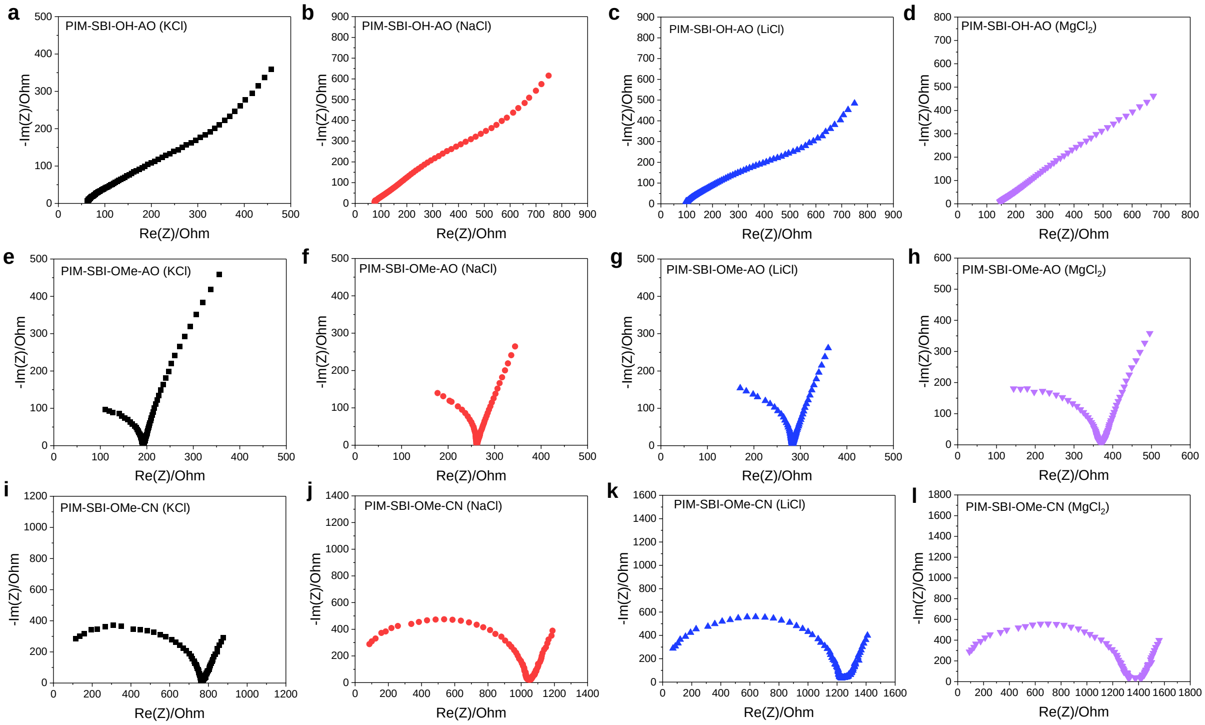

Supplementary Figure S24. Nyquist plots of PIM-SBI membranes. **a**, PIM-SBI-OH-AO in KCl solution. **b**, PIM-SBI-OH-AO in NaCl solution. **c**, PIM-SBI-OH-AO in LiCl solution. **d**, PIM-SBI-OH-AO in $MgCl_2$ solution. **e**, PIM-SBI-OMe-AO in KCl solution. **f**, PIM-SBI-OMe-AO in NaCl solution. **g**, PIM-SBI-OMe-AO in LiCl solution. **h**, PIM-SBI-OMe-AO in $MgCl_2$ solution. **i**, PIM-SBI-OMe-CN in KCl solution. **j**, PIM-SBI-OMe-CN in NaCl solution. **k**, PIM-SBI-OMe-CN in LiCl solution. **l**, PIM-SBI-OMe-CN in $MgCl_2$ solution. Concentrations of all salt solutions are 0.1 M.

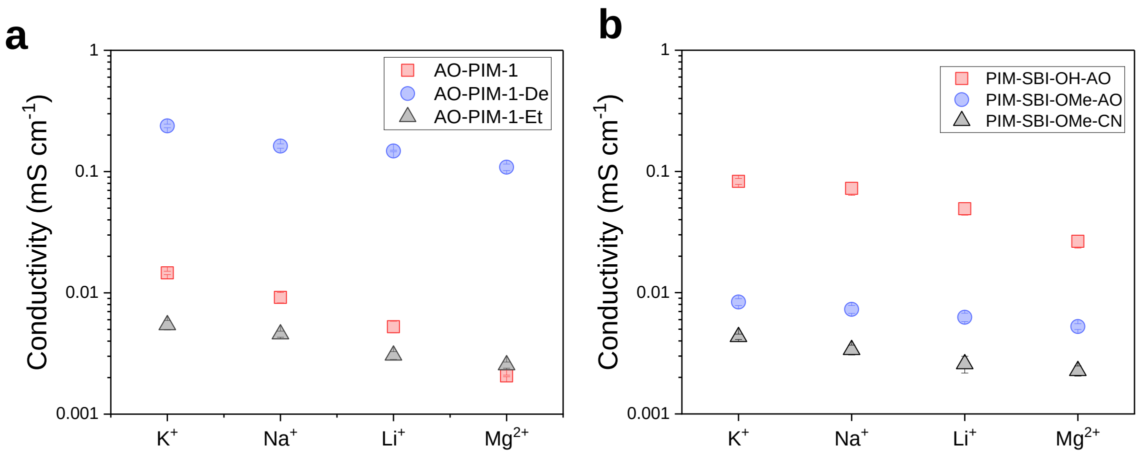

Supplementary Figure S25. Ion conductivity of membranes in four salt solutions (KCl, NaCl, LiCl, and $MgCl_2$). a, Conductivity of AO-PIM-1, AO-PIM-1-De, and AO-PIM-1-Et in different salt solutions. **b**, Conductivity of PIM-SBI-OH-AO, PIM-SBI-OMe-AO, and PIM-SBI-OMe-CN in different salt solutions. The data are presented as the mean ± s.d. (n=3) and the error bars represent the standard deviation (s.d.).

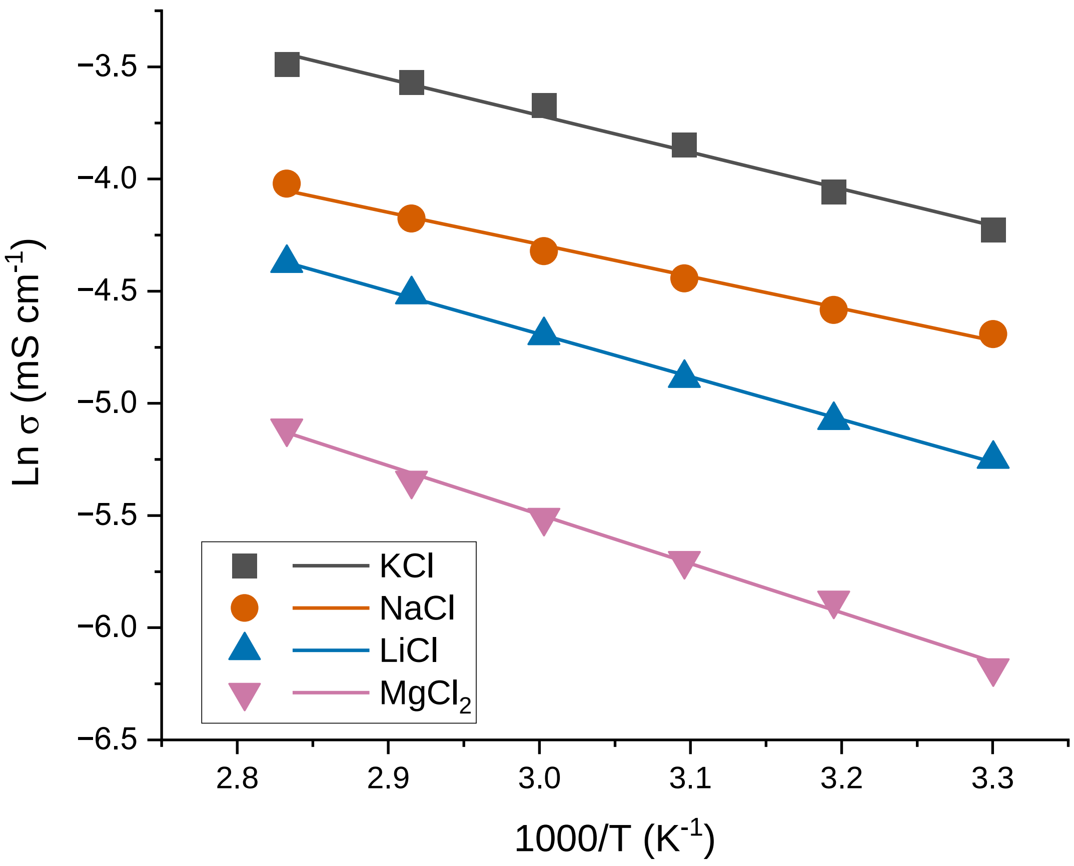

Supplementary Figure S26. Arrhenius plot of ion conductivity as a function of temperature. The activation energy for KCl, NaCl, LiCl, and $MgCl_2$ are 13.6, 11.9, 15.9, 18.2 kJ mol^{-1}, respectively.

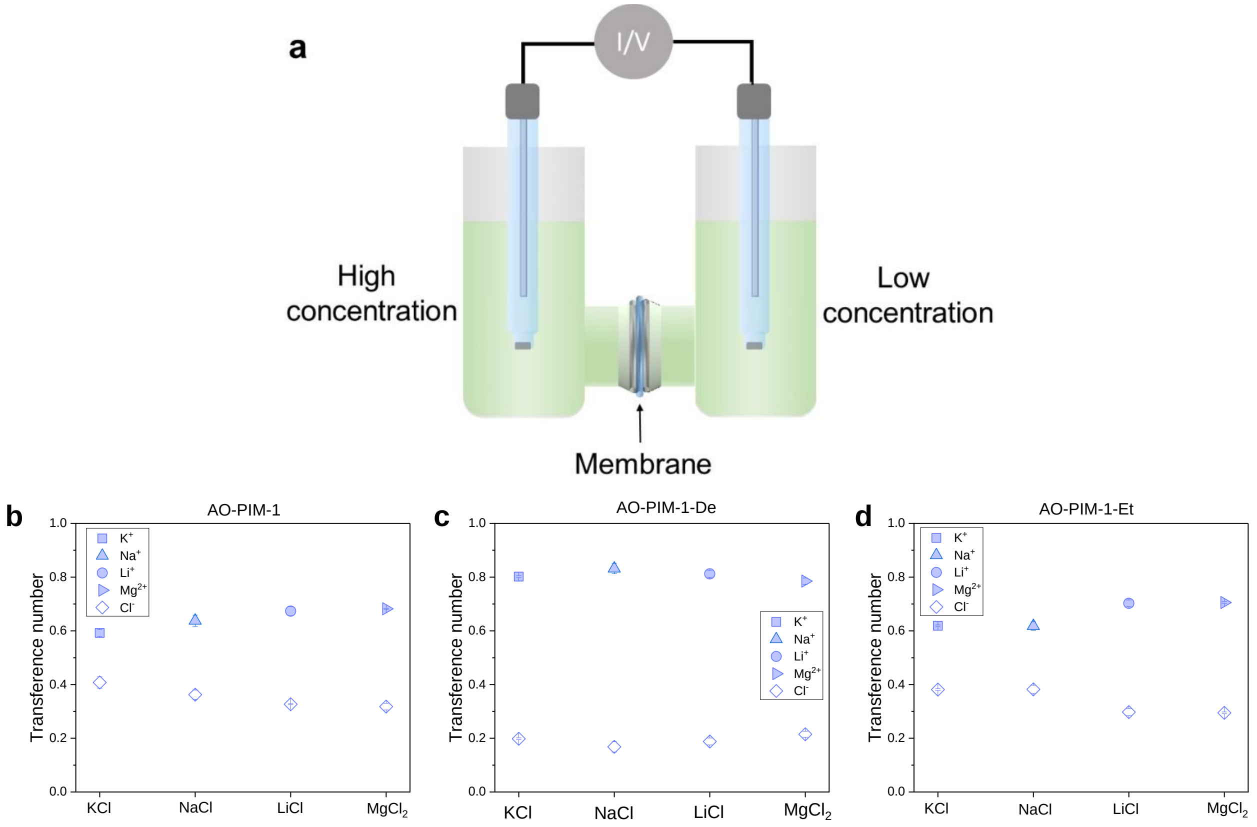

Supplementary Figure S27. Ion transference number. a, Schematic diagram of ion transport number measurement. **b**, Cation and anion transference number in AO-PIM-1. **c**, Cation and anion transference number in AO-PIM-1-De. **d**, Cation and anion transference number in AO-PIM-1-Et. The data are presented as the mean ± s.d. (n=5) and the error bars represent the standard deviation (s.d.). The ion transport numbers quantify the different contributions of cations and anions to conductivity. For example, in AO-PIM-1 membrane, cations carry ~60% of the charge transport, while anions carry ~40%. In AO-PIM-1-De, the cation transference number increases to 0.8 due to electrostatic interactions.

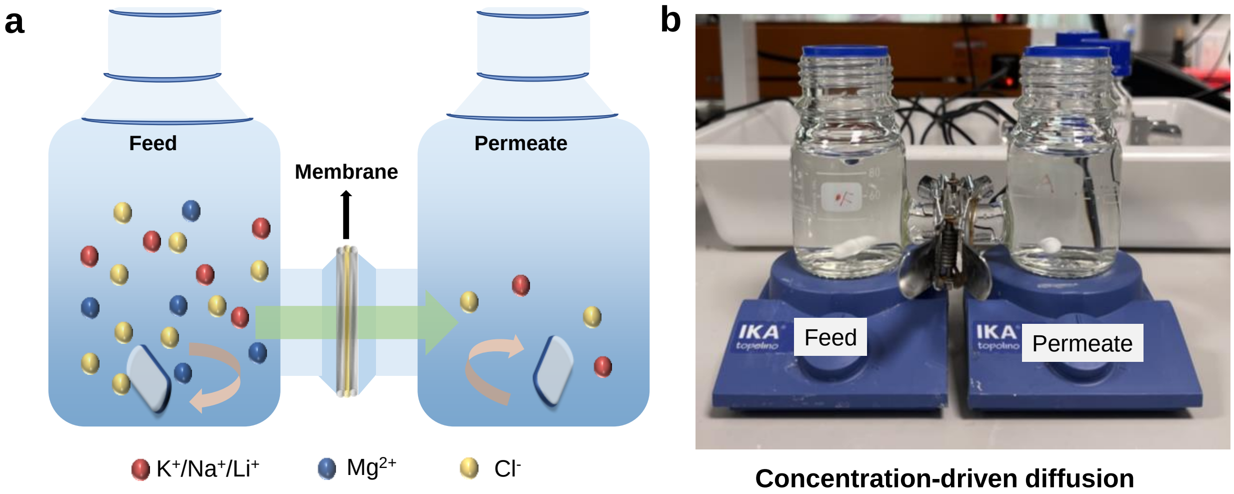

Supplementary Figure S28. Ion permeation through membranes in an H-cell. a, Schematic diagram of ion transport through the membrane in the concentration diffusion process. **b**, Photo of concentration-driven diffusion cell.

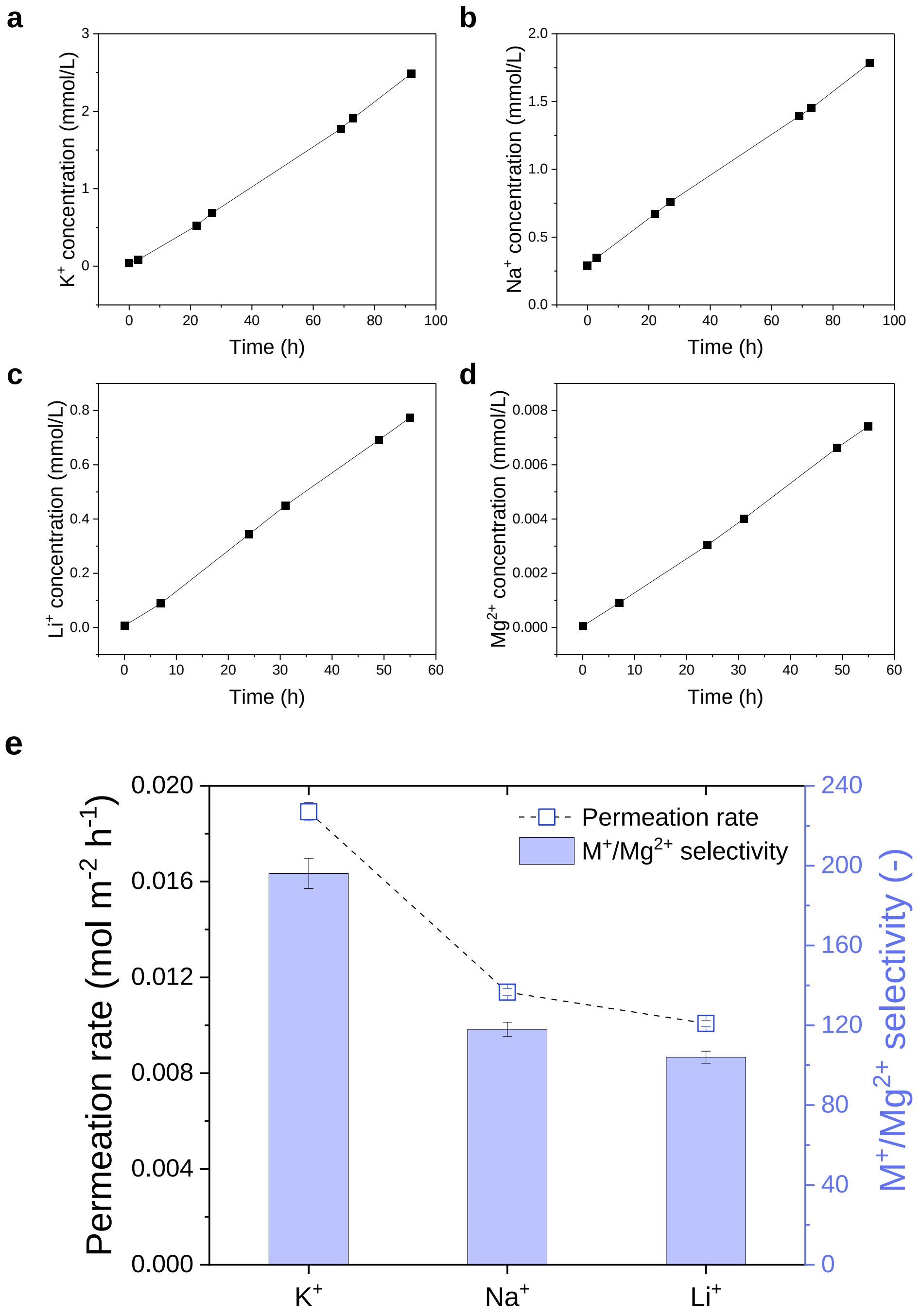

Supplementary Figure S29. Concentration-driven diffusion of single salt ions through AO-PIM-1 membranes. a, The concentration of K^+ through AO-PIM-1 as a function of time (single KCl solution). **b**, The concentration of Na^+ through AO-PIM-1 as a function of time (single NaCl solution). **c**, The concentration of Li^+ through AO-PIM-1 as a function of time (single LiCl solution). **d**, The concentration of Mg^{2+} through AO-PIM-1 as a function of time (single $MgCl_2$ solution). **e**, Comparison of permeation rate and selectivity of AO-PIM-1 for ion separation in single ion component system. In **e**, the error bars of permeation rate data represent the standard errors derived from linear fittings of the concentration profiles, and the error bars of selectivity represent uncertainties derived from the permeation rates.

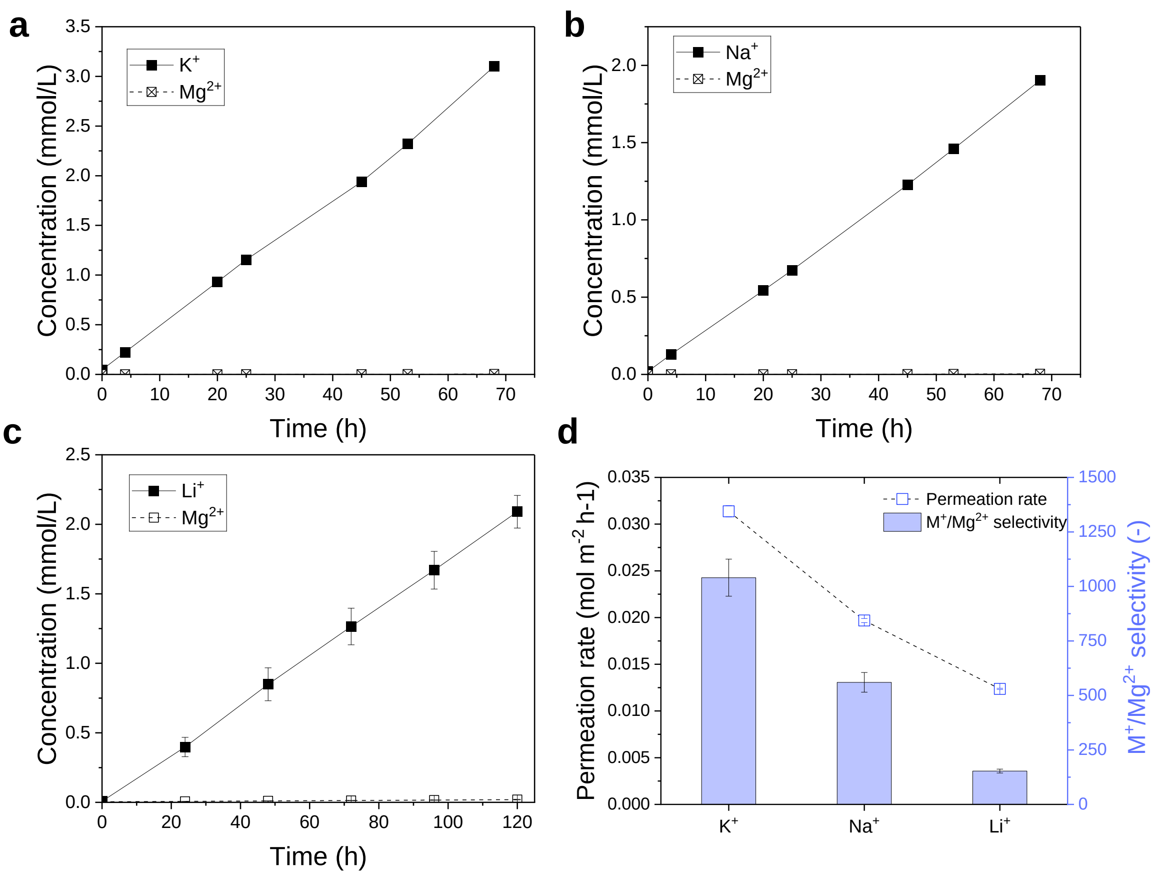

Supplementary Figure S30. Concentration-driven diffusion of binary ion mixtures through AO-PIM-1 membrane. a, The concentration of K^+ through AO-PIM-1 as a function of time (KCl-$MgCl_2$ binary solution). **b**, The concentration of Na^+ through AO-PIM-1 as a function of time (NaCl-$MgCl_2$ binary solution). **c**, The concentration of Li^+ through AO-PIM-1 as a function of time (LiCl-$MgCl_2$ binary solution). **d**, Comparison of permeation rate and selectivity of AO-PIM-1 for ion separation in different systems. In **c**, the data are presented as the mean ± s.d. (n=3) and the error bars represent the standard deviation (s.d.). In **d**, the error bars of permeation rate data represent the standard errors derived from linear fittings of salt concentration profiles of three independent experiments, and the error bars of selectivity represent uncertainties derived from the permeation rates.

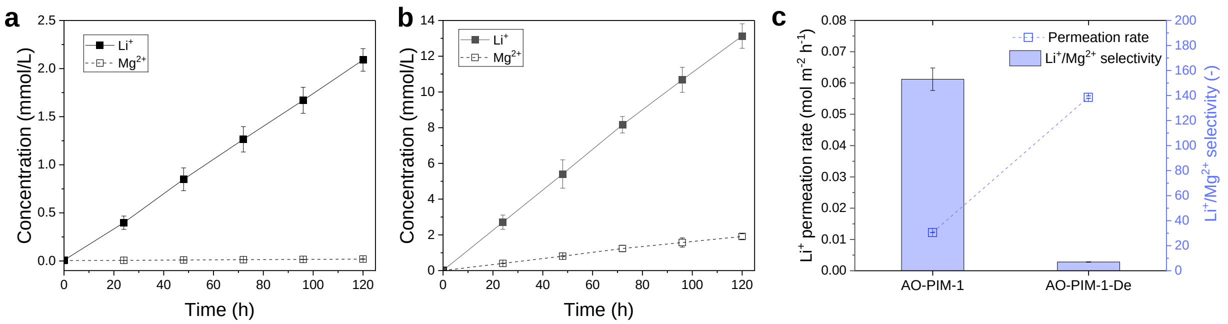

Supplementary Figure S31. Comparison of concentration-driven diffusion through AO-PIM-1 and AO-PIM-1-De membranes. a, The concentration of Li^+ and Mg^{2+} through AO-PIM-1 as a function of time ($LiCl$-$MgCl_2$ binary mixture). **b**, The concentration of Li^+ and Mg^{2+} through AO-PIM-1-De as a function of time ($LiCl$-$MgCl_2$ binary mixture). **c**, Comparison of permeation rate and selectivity of AO-PIM-1 and AO-PIM-1-De for ion separation. In a and b, the data are presented as the mean ± s.d. (n=3) and the error bars represent the standard deviation (s.d.). In **c**, the error bars of permeation rate data represent the standard errors derived from linear fittings of salt concentration profiles of three independent experiments, and the error bars of selectivity data represent uncertainties derived from the permeation rates.

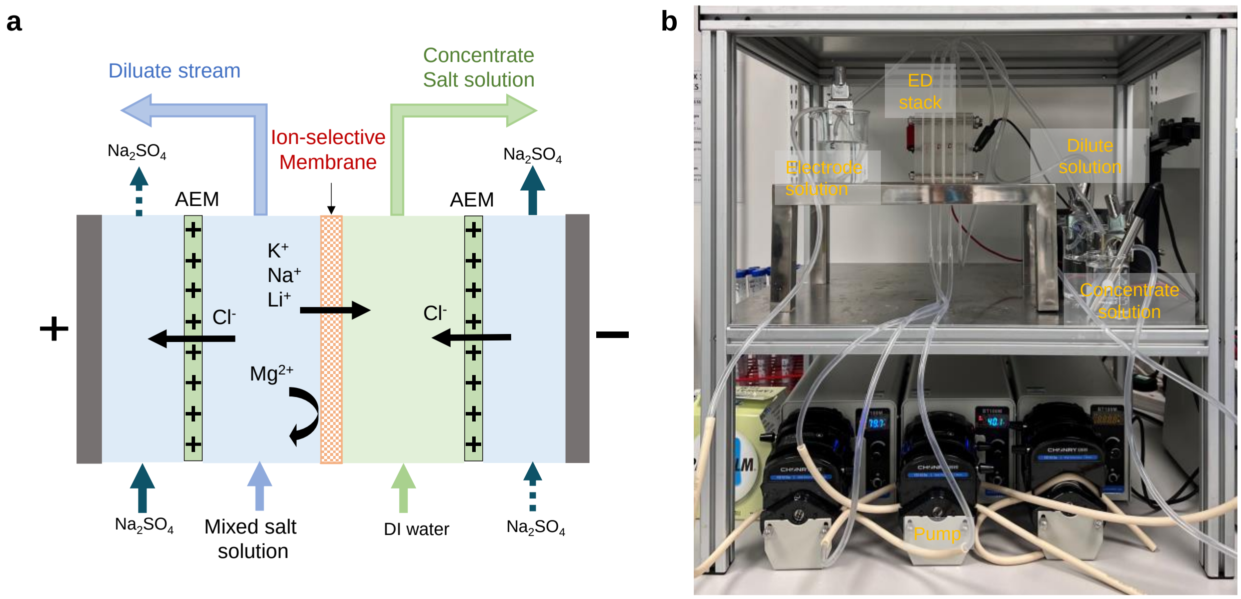

Supplementary Figure S32. Electrodialysis testing system. **a**, Schematic diagram of electrodialysis process for monovalent/divalent ion separation. **b**, Photo of a lab-scale electrodialysis cell for ion separation, with effective area of 2 cm^2.

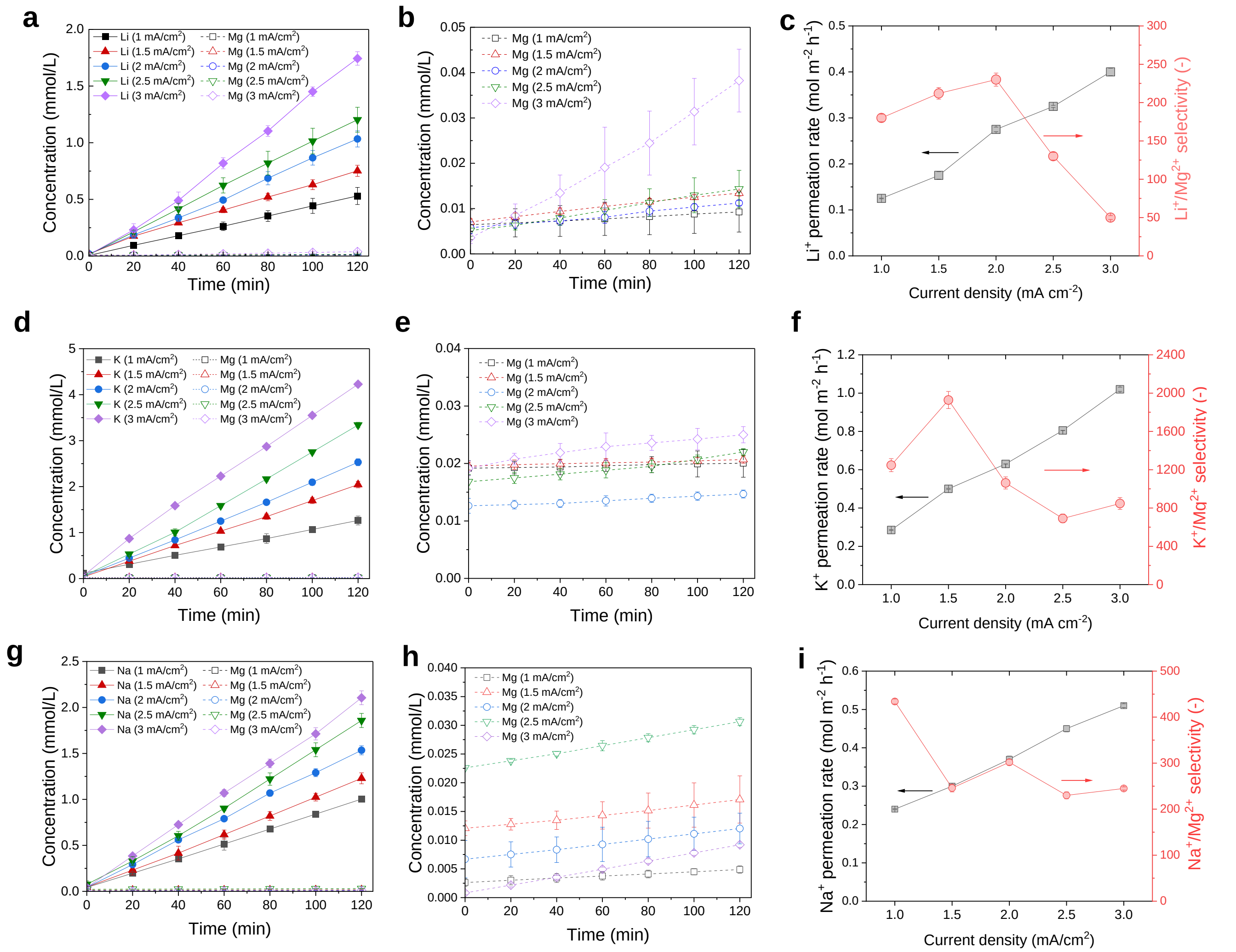

Supplementary Figure S33. Electrodialysis ion separation performance of binary mixtures with AO-PIM-1 membranes at different current densities. **a**, Concentrations of Li^+ and Mg^{2+} in concentrate chamber under different current densities ($LiCl$-$MgCl_2$ binary mixture). **b**, Concentrations of Mg^{2+} in concentrate chamber under different current densities ($LiCl$-$MgCl_2$ binary mixture). **c**, Permeation rate and selectivity of AO-PIM-1 membrane for Li/Mg separation under different current densities. **d**, Concentrations of K^+ and Mg^{2+} in concentrate chamber under different current densities (KCl-$MgCl_2$ binary mixture). **e**, Concentrations of Mg^{2+} in concentrate chamber under different current densities (KCl-$MgCl_2$ binary mixture). **f**, Permeation rate and selectivity of AO-PIM-1 for K/Mg separation. **g**, Concentrations of Na^+ and Mg^{2+} in concentrate chamber under different current densities ($NaCl$-$MgCl_2$ binary mixture). **h**, Concentrations of Mg^{2+} in concentrate chamber under different current densities ($NaCl$-$MgCl_2$ binary mixture). **i**, Permeation rate and selectivity of AO-PIM-1 for $NaCl$-$MgCl_2$ binary mixture separation. In **a-b**, **d-e**, and **g-h**, the data are presented as the mean ± s.d. (n=3) and the error bars represent the standard deviation (s.d.). In **c, f**, **i**, the error bars of permeation rate data represent the standard errors derived from linear fittings of the salt concentration profiles of three independent experiments, and the error bars of selectivity data represent uncertainties derived from the permeation rates.

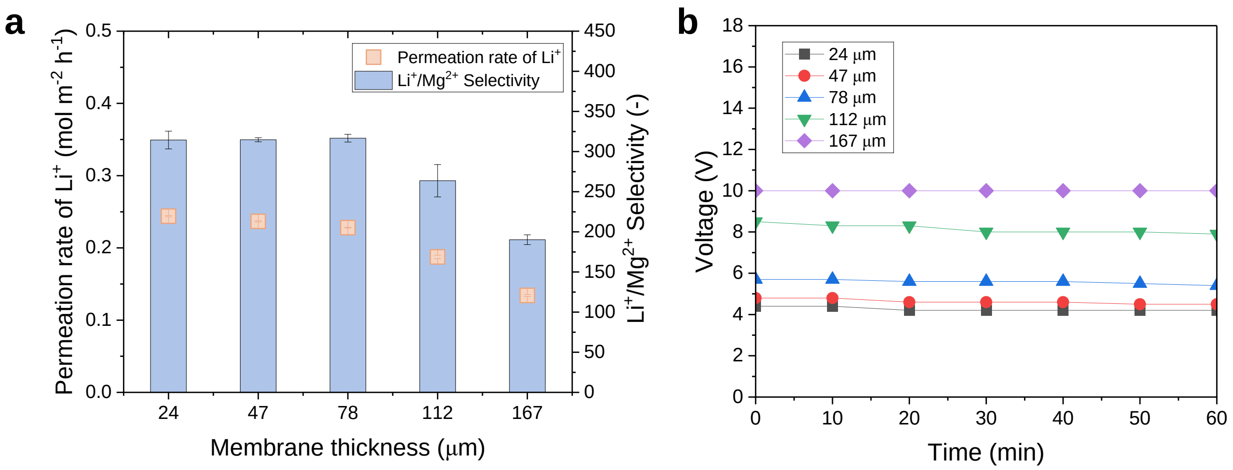

Supplementary Figure S34. Electrodialysis separation performance of AO-PIM-1 membranes with varied thickness. a, Ion permeation rate and selectivity. **b**, Variation of voltage with time. In **a**, the error bars of permeation rate data represent the standard errors derived from linear fittings of salt concentration profiles, and the error bars of selectivity data represent uncertainties derived from the permeation rates.

Under the driving force of the electric field, the ion permeation rate is quite different from that under concentration-driven diffusion. Here we listed the ion permeation rate calculations under the two phenomena:

In concentration-driven diffusion process, the equation of ion permeation rate is:

$$J = \frac{D \times C}{l} \tag{10}$$

Where J is the ion permeation rate (mol m^{-2} h^{-1}), D is diffusion coefficient of ions in the membrane (m^2 h^{-1}), C is the concentration gradient of ions across the membrane (mol m^{-3}), l is the thickness of the membrane (m).

While in the electrodialysis process, ion permeation rate is determined by the following equation, derived from Nernst–Planck equation:

$$J = -D \times \left(\frac{dC}{dx}\right) + \frac{zDFC}{RT} \times \frac{dV}{dx} \tag{11}$$

Where J is the ion permeation rate (mol m^{-2} h^{-1}), D is diffusion coefficient of ions in the membrane (m^2 h^{-1}), *dC/dx* is the concentration gradient across the membrane (mol m^{-3} m^{-1}), *z* is the ion valence. F is the Faraday constant (96485 C mol^{-1}), *dV/dx* is the electric field applied across the membrane (V m^{-1}), *R* is the gas constant (8.314J mol^{-1} K^{-1}), *T* is the temperature (K).

As we can see from the above two equations, in concentration diffusion process, ion permeation rate is directly in diverse proportion with membrane thickness, as described by Fick's first law. In electrodialysis tests, the electric field plays a more important role in driving the ion transport. In our electrodialysis testing, we applied a constant current density (2 mA cm^{-2}), thicker membranes led to slightly higher resistance for ion transport, as reflected by the higher voltage value (the voltage of the whole cell, not the voltage across the membrane). Hence lower ion permeation rates were observed when the membranes were thicker than 100μm. For membrane with lower thickness, the influence of membrane thickness is much weaker under the same current density (2 mA cm^{-2}), because the ion permeation across the membranes correspond to the electrons through the external circuit.

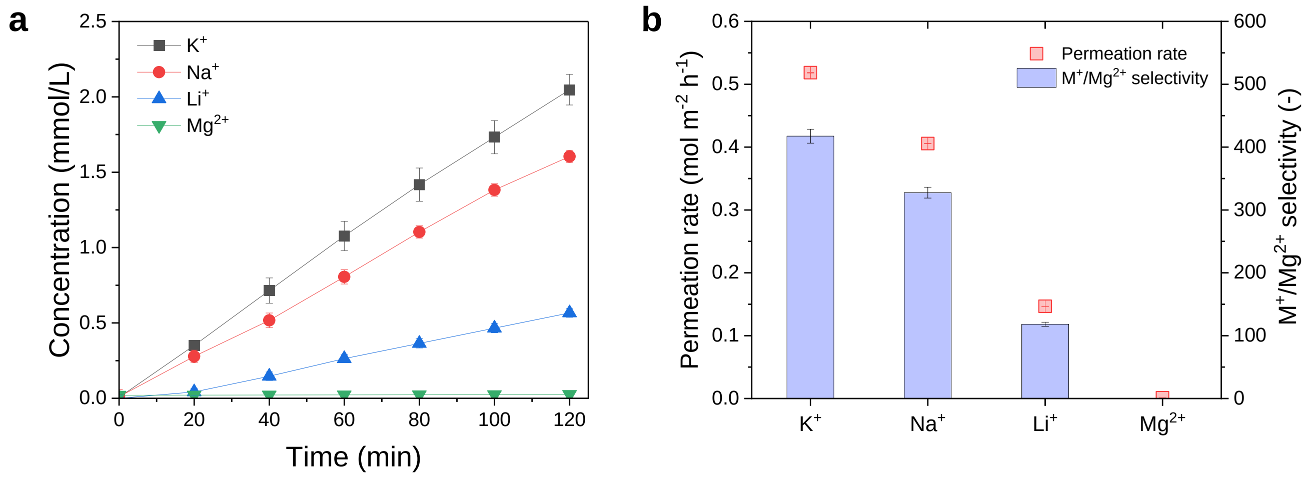

Supplementary Figure S35. Electrodialysis separation of mixed K/Na/Li/Mg salt solutions with AO-PIM-1 membrane. a, Ion concentrations profiles in concentrate chamber. **b**, Ion permeation rate and selectivity. The salt concentrations are 0.1 M each. In **a**, the data are presented as the mean ± s.d. (n=3) and the error bars represent the standard deviation (s.d.). In **b**, the error bars of permeation rate data represent the standard errors derived from linear fittings of salt concentration profiles of three independent experiments, and the error bars of selectivity data represent uncertainties derived from the permeation rates.

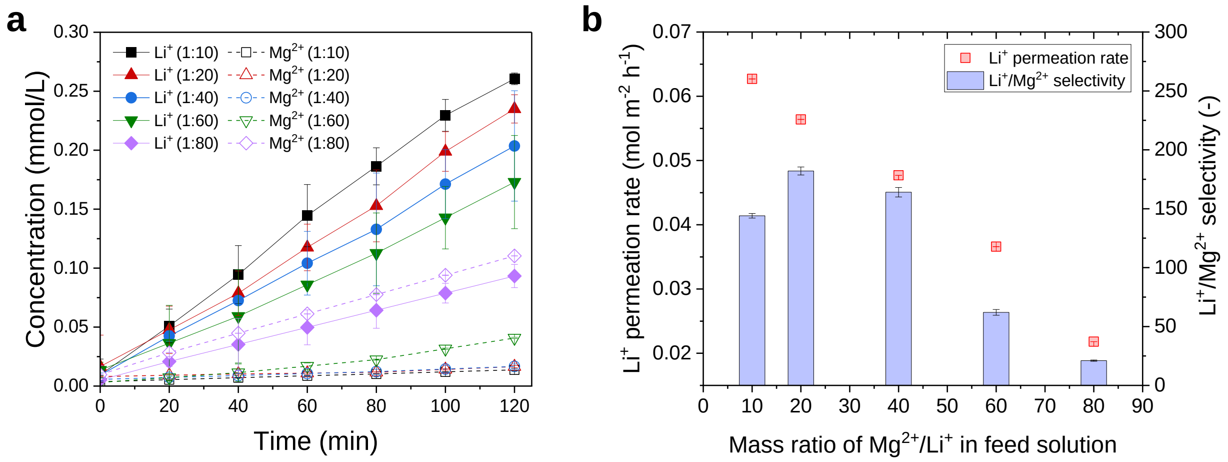

Supplementary Figure S36. Ion separation of AO-PIM-1 with different initial Li/Mg mass ratios. **a**, Ion concentrations in concentrate chamber with AO-PIM-1 membrane under different initial mass ratios of Li/Mg as a function of time (Mg/Li mass ratios=10, 20, 40, 60, 80). **b**, Permeation rate and selectivity under different initial mass ratios of Mg/Li. The LiCl salt concentration is fixed at 0.01mol L^{-1}. In **a**, the data are presented as the mean ± s.d. (n=3) and the error bars represent the standard deviation (s.d.). In **b**, the error bars of permeation rate data represent the standard errors derived from linear fittings of salt concentration profiles of three independent experiments, and the error bars of selectivity data represent uncertainties derived from the permeation rates.

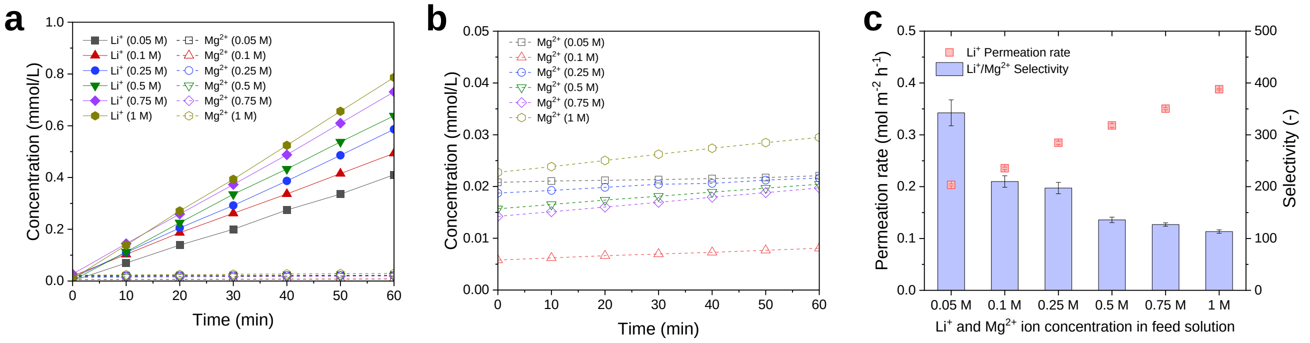

Supplementary Figure S37. Ion separation performance of AO-PIM-1 membrane with different feed concentrations. a, Ion concentrations in concentrate chamber with AO-PIM-1 membrane with varied feed concentration (0.05 M to 1 M). **b**, Concentration profiles of Mg^{2+} in concentrate chamber as a function of time. **c**, Li^+ permeation rate and Li^+/Mg^{2+} selectivity as a function of feed concentration. In **c**, the error bars of permeation rate data represent the standard errors derived from linear fittings of salt concentration profiles, and the error bars of selectivity data represent uncertainties derived from the permeation rates.

With the feed concentration increased from 0.05M to 1M, the Li^+ permeation rate increased from 0.2 to 0.4 mol m^{-2} h^{-1}, while the Li^+/Mg^{2+} selectivity decreased from 350 to about 100. In the electrodialysis process, it was generally reported that the ion selectivity decreases with increasing feed solution concentration. The selectivity change could be attributed to several factors, including back diffusion, charge screening, increased ion competition, and concentration polarization[23]. At higher feed ion concentrations, both Li^+ and Mg^{2+} ions are present in greater numbers near the membranes. This increased concentration might reduce the ability of the membrane to differentiate between ions based on size, charge, or mobility. Consequently, Mg^{2+} ions, which are typically less mobile due to its higher charge and hydration energy, compete more effectively with Li^+ for transport through the membrane. Potentially more Mg^{2+} are partitioning into the pore entrances due to their strong binding with the functional groups, and block the pathways for monovalent ions. At high concentrations, the driving force for ion migration (the electric field) interacts more uniformly with ions, making it harder to selectively separate them based on their mobility or charge-to-size ratio. Literature work suggests that the electromigration of Mg^{2+} ions is more influenced by the Mg^{2+} concentration[24]. Another reason might be due to the change of solvation shells. Li^+ and Mg^{2+} have different hydration energies, but at high concentrations, the hydration shells of ions can overlap or interact, which could also limit the selectivity. To maintain selectivity in electrodialysis processes, it is crucial to optimize operating conditions, such as diluting the feed solution, or operating within an optimal concentration range. Nevertheless, the AO-PIM-1 membrane maintained high selectivity (>100) at high concentrations.

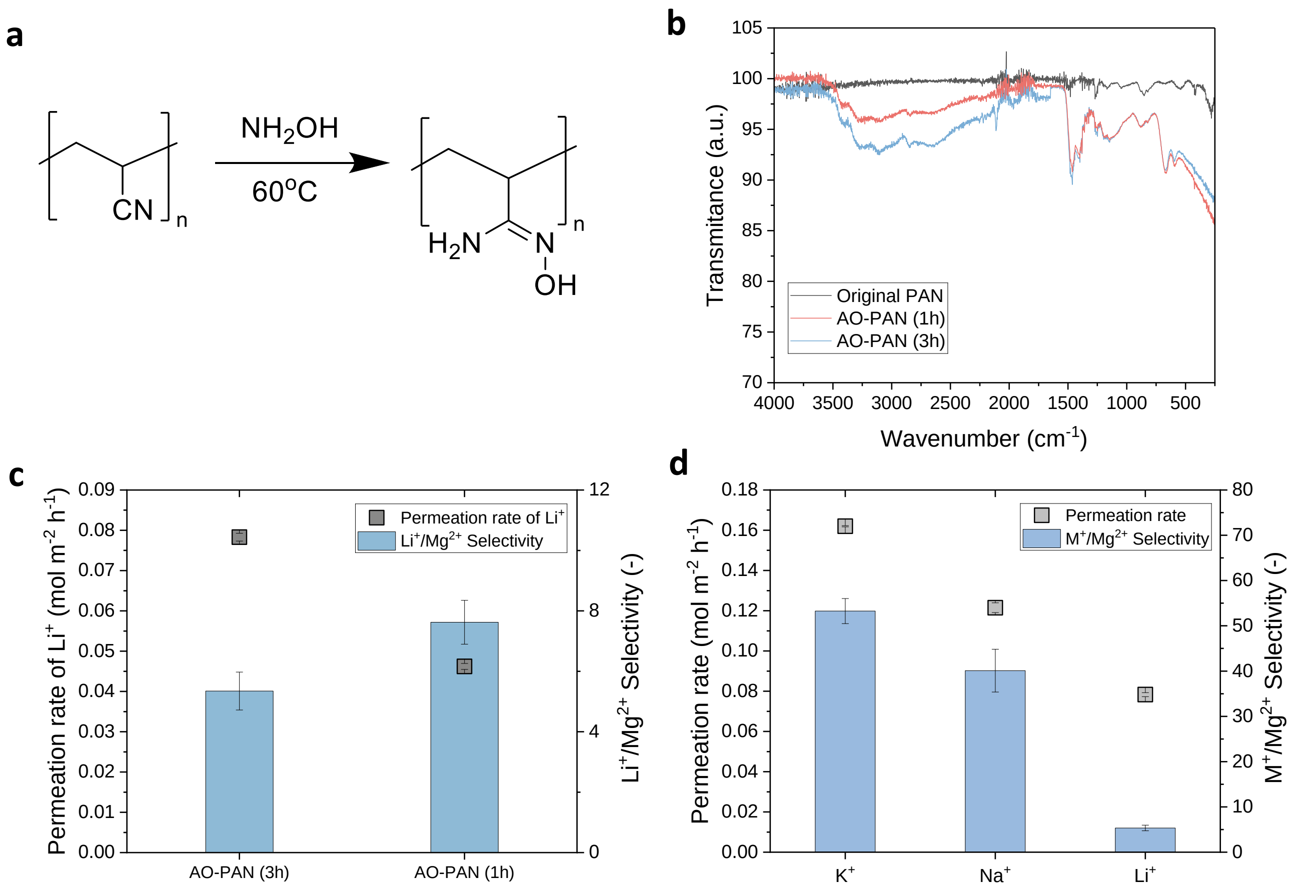

Supplementary Figure S38. Electrodialysis separation performance of AO-PAN membrane. a, Synthesis of AO-PAN. **b**, FTIR spectra of PAN, AO-PAN (1h) and AO-PAN (3h). **c**, Ion permeation rate and selectivity for Li/Mg binary mixture for AO-PAN (1 h) and AO-PAN (3h). **d**, Ion permeation rate and selectivity of AO-PAN (3h) for K/Na/Li/Mg mixtures. The salt concentrations are 0.1 M each. In **c-d**, the error bars of permeation rate data represent the standard errors derived from linear fittings of salt concentration profiles of three independent experiments, and the error bars of selectivity data represent uncertainties derived from the permeation rates.

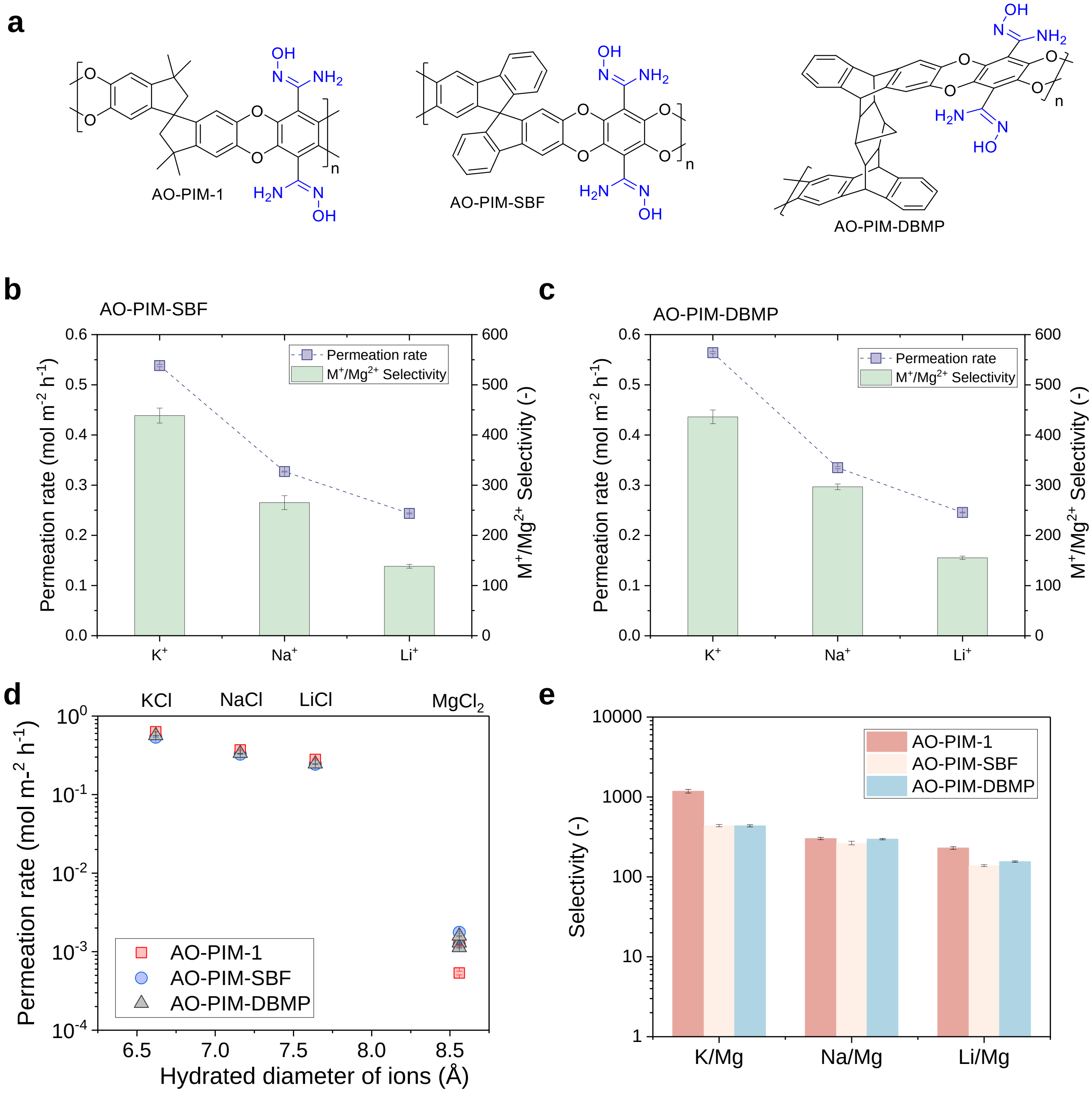

Supplementary Figure S39. Electrodialysis separation performance of AO-PIM membranes with more rigid backbones. a, Chemical structures of AO-PIM-1, AO-PIM-SBF, and AO-PIM-DBMP. **b**, Ion permeation rate and selectivity of AO-PIM-SBF for separation of K/Mg, Na/Mg, Li/Mg binary mixtures. **c**, Ion permeation rate and selectivity of AO-PIM-DBMP for separation of K/Mg, Na/Mg, Li/Mg binary mixtures. **d**, Comparison of permeation rates of AO-PIM-1, AO-PIM-SBF, and AO-PIM-DBMP. **e**, Comparison of selectivity of AO-PIM-1, AO-PIM-SBF, and AO-PIM-DBMP. In **b-e**, the error bars of permeation rate data represent the standard errors derived from linear fittings of salt concentration profiles of three independent experiments, and the error bars of selectivity data represent uncertainties derived from the permeation rates.

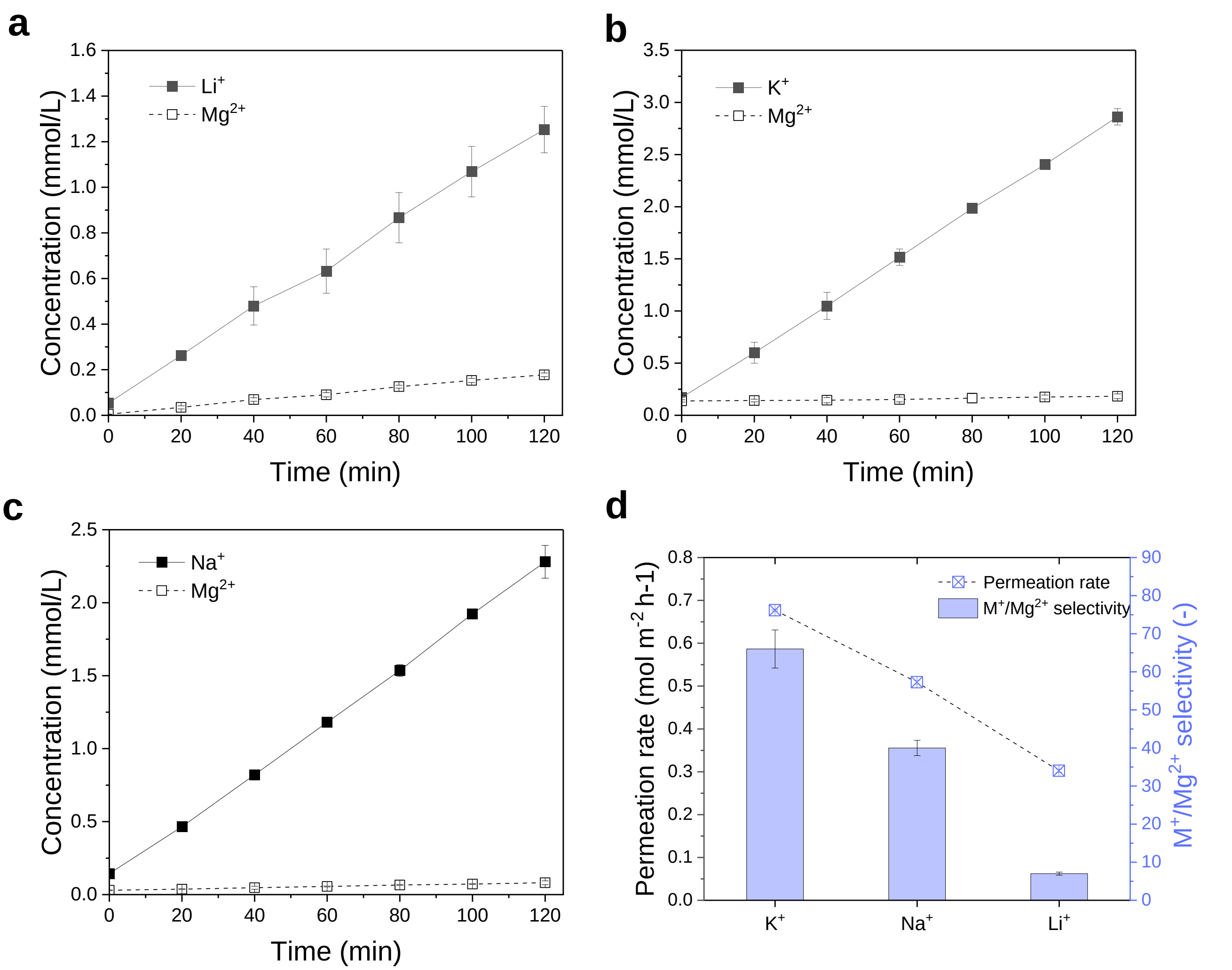

Supplementary Figure S40. Electrodialysis separation of binary mixtures through AO-PIM-1-De membrane. **a**, Concentration profiles of Li^+ and Mg^{2+} in concentrate chamber as a function of time ($LiCl$-$MgCl_2$ binary solution). **b**, Concentration profiles of K^+ and Mg^{2+} in concentrate chamber as a function of time (KCl-$MgCl_2$ binary solution). **c**, Concentration profiles of Na^+ and Mg^{2+} in concentrate chamber as a function of time ($NaCl$-$MgCl_2$ binary solution). **d**, Permeation rate and selectivity of AO-PIM-1-De for ion separation. In **a-c**, the data are presented as the mean ± s.d. (n=3) and the error bars represent the standard deviation (s.d.). In **d**, the error bars of permeation rate data represent the standard errors derived from linear fittings of salt concentration profiles of three independent experiments, and the error bars of selectivity data represent uncertainties derived from the permeation rates.

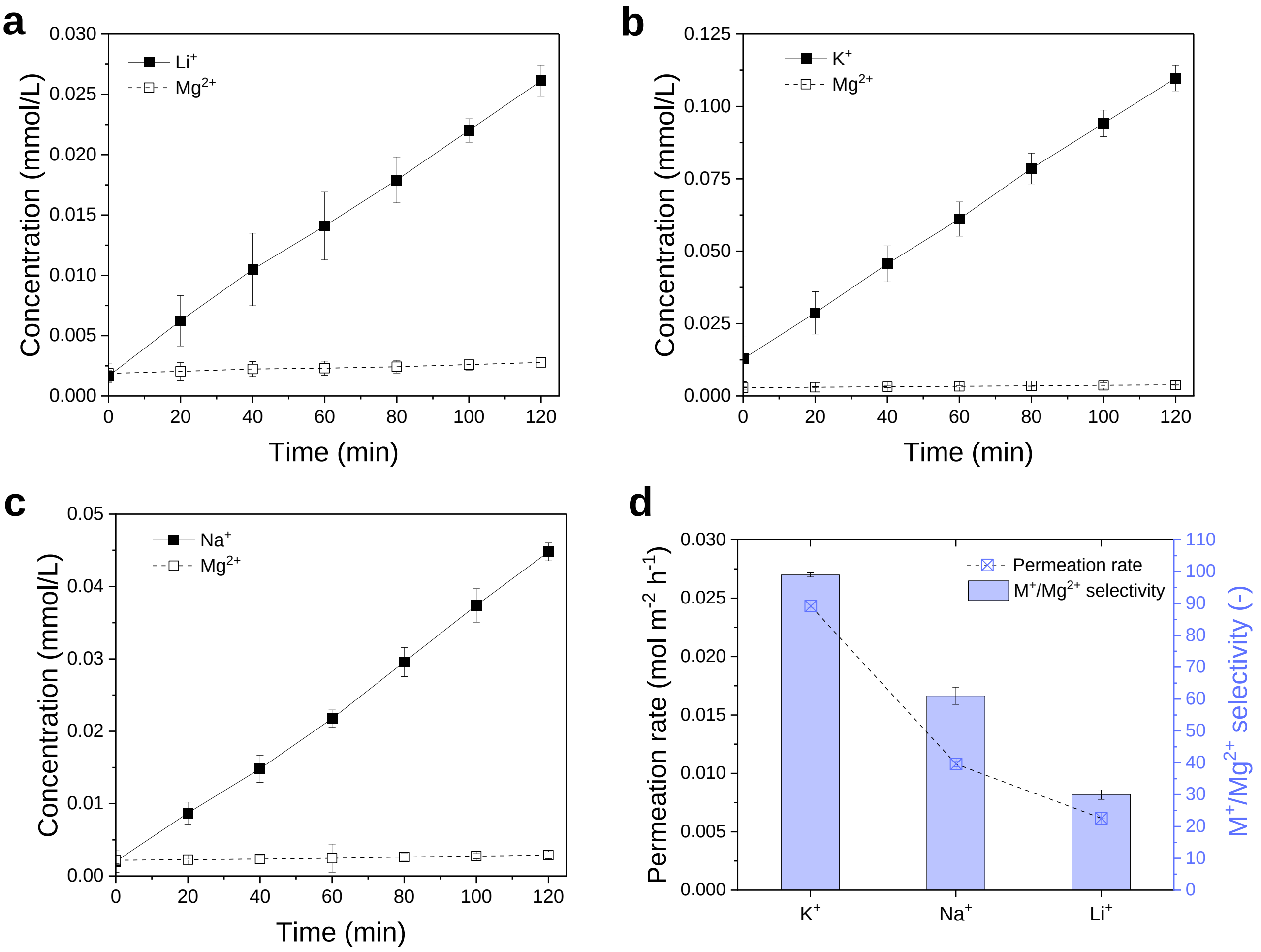

Supplementary Figure S41. Electrodialysis separation of binary mixtures through AO-PIM-1-Et membrane. a, Concentration profiles of Li^+ and Mg^{2+} in concentrate chamber as a function of time ($LiCl$-$MgCl_2$ binary solution). **b**, Concentration profiles of K^+ and Mg^{2+} in concentrate chamber as a function of time ($LiCl$-$MgCl_2$ binary solution). **c**, Concentration profiles of Na^+ and Mg^{2+} in concentrate chamber as a function of time ($LiCl$-$MgCl_2$ binary solution). **d**, The permeation rate and selectivity of AO-PIM-1-Et for ion separation. In **a-c**, the data are presented as the mean ± s.d. (n=3) and the error bars represent the standard deviation (s.d.). In **d**, the error bars of permeation rate data represent the standard errors derived from linear fittings of salt concentration profiles of three independent experiments, and the error bars of selectivity data represent uncertainties derived from the permeation rates.

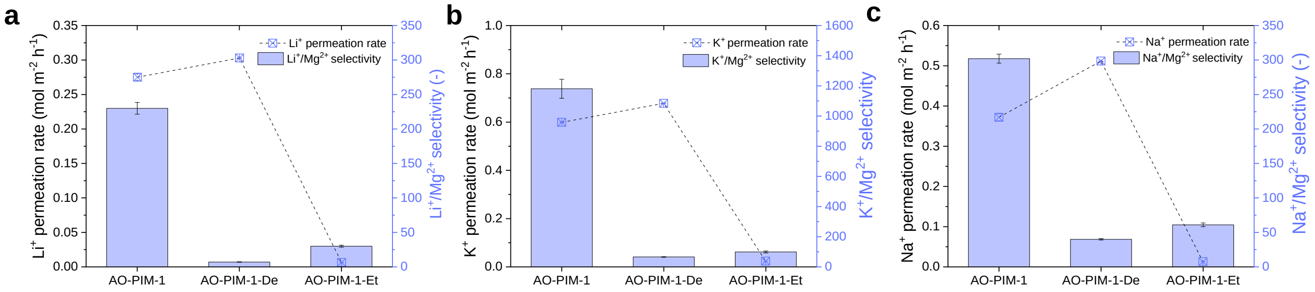

Supplementary Figure S42. Comparison of permeation rate and selectivity of AO-PIM membranes for ion separation. a, Comparison of permeation rate and selectivity of AO-PIM-1, AO-PIM-1-De, and AO-PIM-1-Et for Li/Mg separation. **b**, Comparison of permeation rate and selectivity of AO-PIM-1, AO-PIM-1-De, and AO-PIM-1-Et for K/Mg separation. **c**, Comparison of permeation rate and selectivity of AO-PIM-1, AO-PIM-1-De, and AO-PIM-1-Et for Na/Mg separation. The error bars of permeation rate data represent the standard errors derived from linear fittings of salt concentration profiles of three independent experiments, and the error bars of selectivity data represent uncertainties derived from the permeation rates.

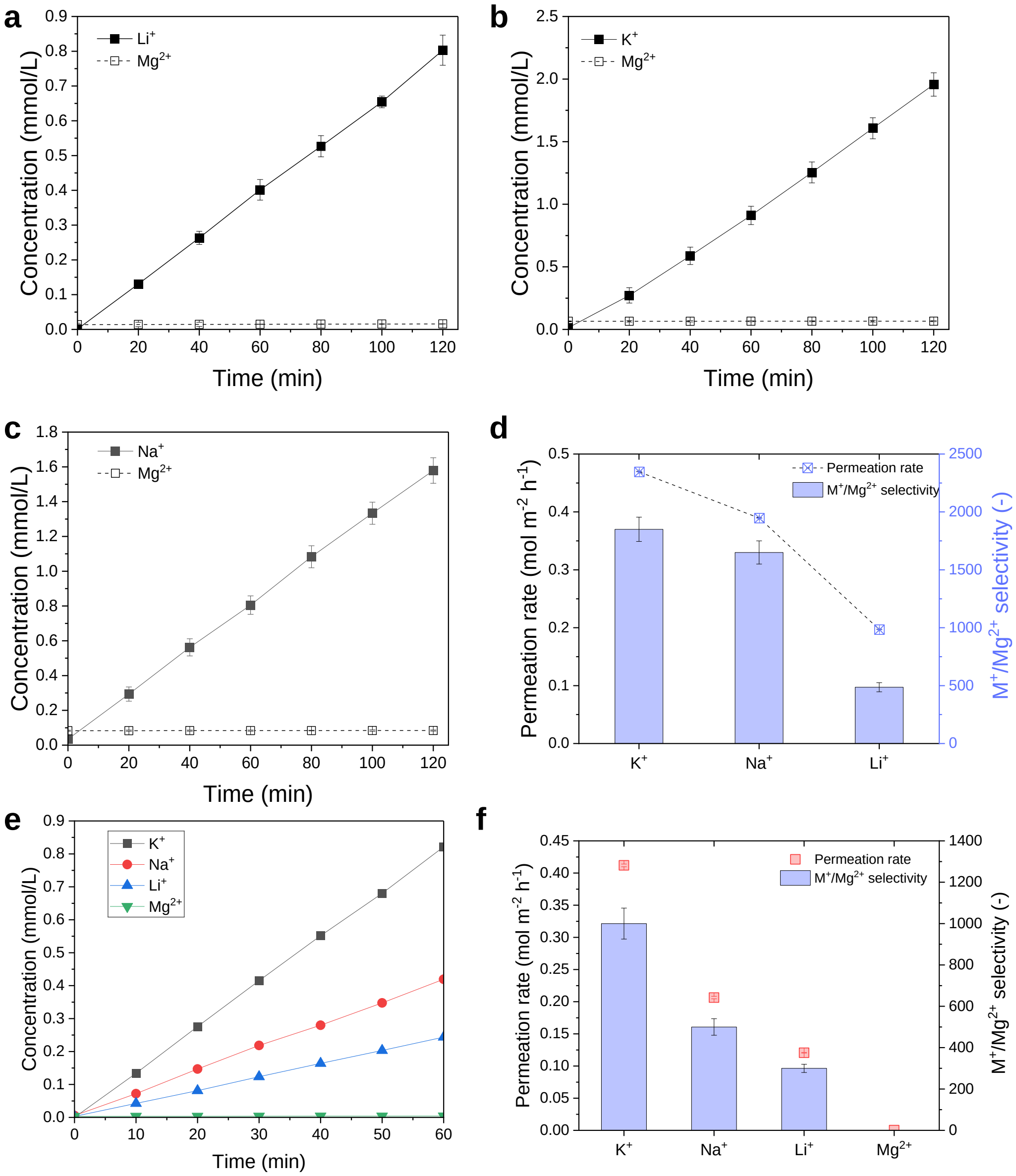

Supplementary Figure S43. Electrodialysis ion separation performance of PIM-SBI-OH-AO membrane in binary mixtures and K/Na/Li/Mg mixtures. a, Concentration profiles of Li^+ and Mg^{2+} in concentrate chamber as a function of time (Li/Mg binary system). **b**, Concentration profiles of K^+ and Mg^{2+} in concentrate chamber as a function of time (K/Mg binary system). **c**, Concentration profiles of Na^+ and Mg^{2+} in concentrate chamber as a function of time (Na/Mg binary system). **d**, Permeation rate and selectivity of PIM-SBI-OH-AO for ion separation. **e**, Concentration profiles of K^+, Na^+, Li^+, and Mg^{2+} in concentrate chamber as a function of time (KCl/NaCl/LiCl/$MgCl_2$ mixture, 0.1 M each). **f**, Permeation rate and selectivity of PIM-SBI-OH-AO for ion separation. In **a-c**, the data are presented as the mean ± s.d. (n=3) and the error bars represent the standard deviation (s.d.). In **d**, the error bars of permeation rate data represent the standard errors derived from linear fittings of salt concentration profiles of three independent experiments, and the error bars of selectivity data represent uncertainties derived from the permeation rates. In **f**, the error bars of permeation rate data represent the standard errors derived from linear fittings of salt concentration profiles, and the error bars of selectivity data represent uncertainties derived from the permeation rates.

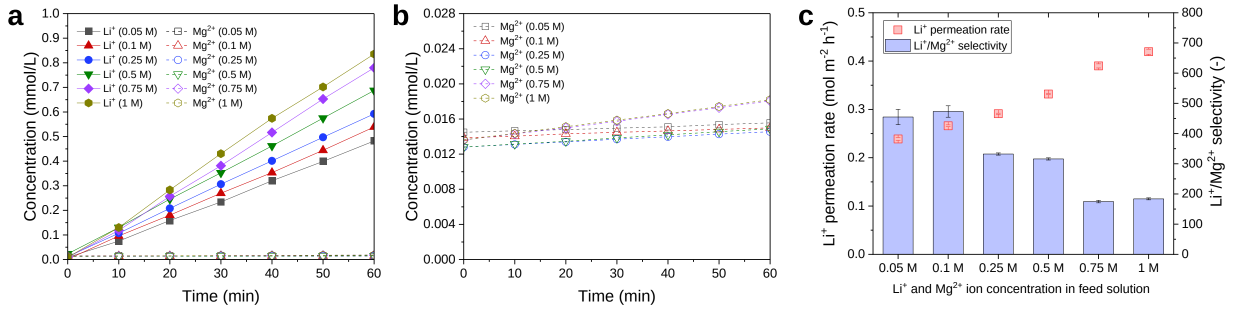

Supplementary Figure S44. Electrodialysis performance of PIM-SBI-OH-AO membrane with different feed concentrations. a, Concentration profiles of Li^+ and Mg^{2+} in concentrate chamber as a function of time (Li/Mg binary system). The Li^+ and Mg^{2+} concentration in feed solution varied from 0.05M to 1M. **b**, Concentration profiles of Mg^{2+} in concentrate chamber as a function of time. **c**, Permeation rate and selectivity of PIM-SBI-OH-AO for ion separation. In **c**, the error bars of permeation rate data represent the standard errors derived from linear fittings of salt concentration profiles, and the error bars of selectivity data represent uncertainties derived from the permeation rates.

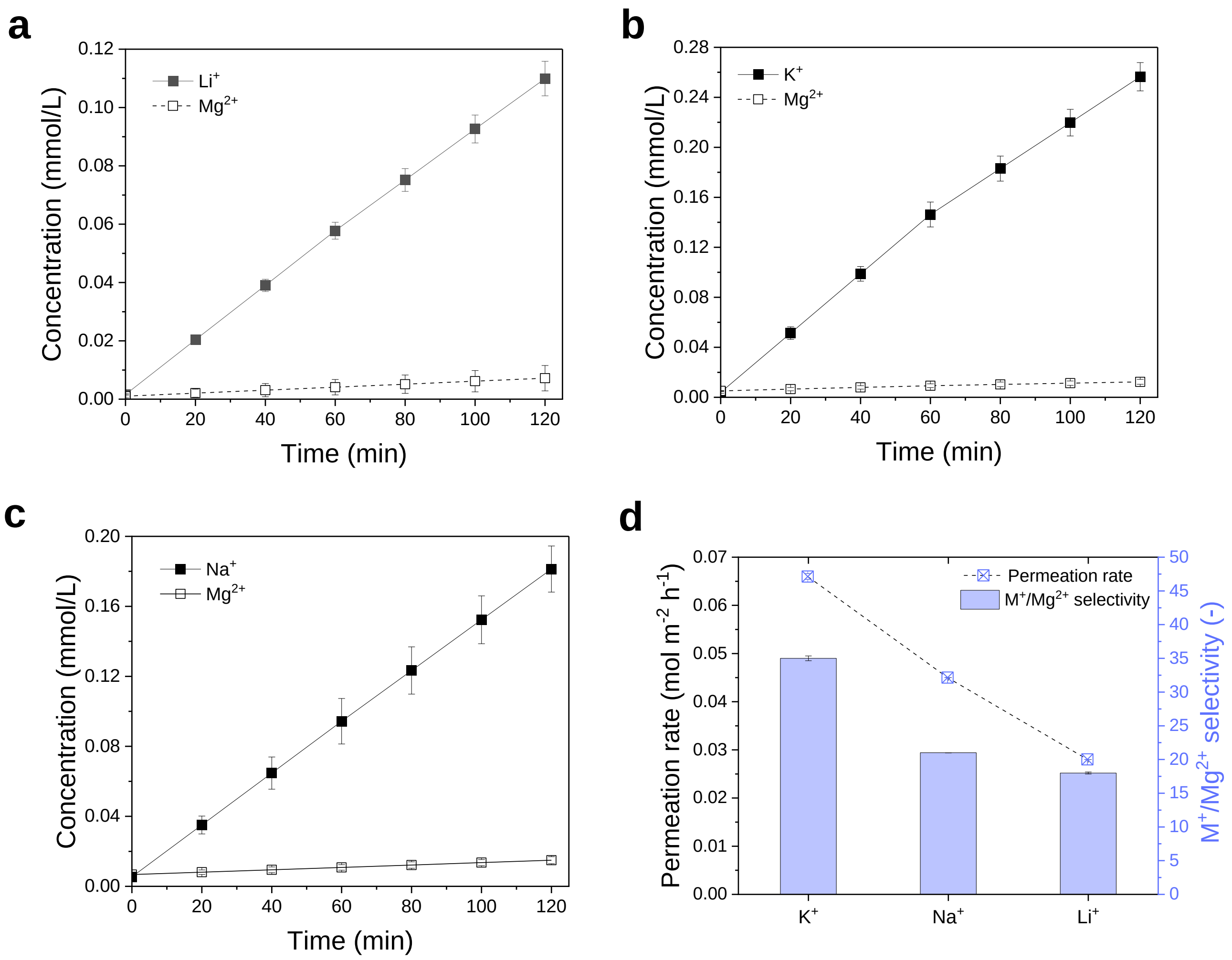

Supplementary Figure S45. Electrodialysis separation of binary mixtures through PIM-SBI-OMe-AO membrane. a, Concentration profiles of Li^+ and Mg^{2+} in concentrate chamber as a function of time ($LiCl$-$MgCl_2$ binary solution). **b**, Concentration profiles of K^+ and Mg^{2+} in concentrate chamber as a function of time (KCl-$MgCl_2$ binary solution). **c**, Concentration profiles of Na^+ and Mg^{2+} in concentrate chamber as a function of time ($NaCl$-$MgCl_2$ binary solution). **d**, Permeation rate and selectivity of PIM-SBI-OMe-AO for ion separation. In **a-c**, the data are presented as the mean ± s.d. (n=3) and the error bars represent the standard deviation (s.d.). In **d**, the error bars of permeation rate data represent the standard errors derived from linear fittings of salt concentration profiles of three independent experiments, and the error bars of selectivity data represent uncertainties derived from the permeation rates.

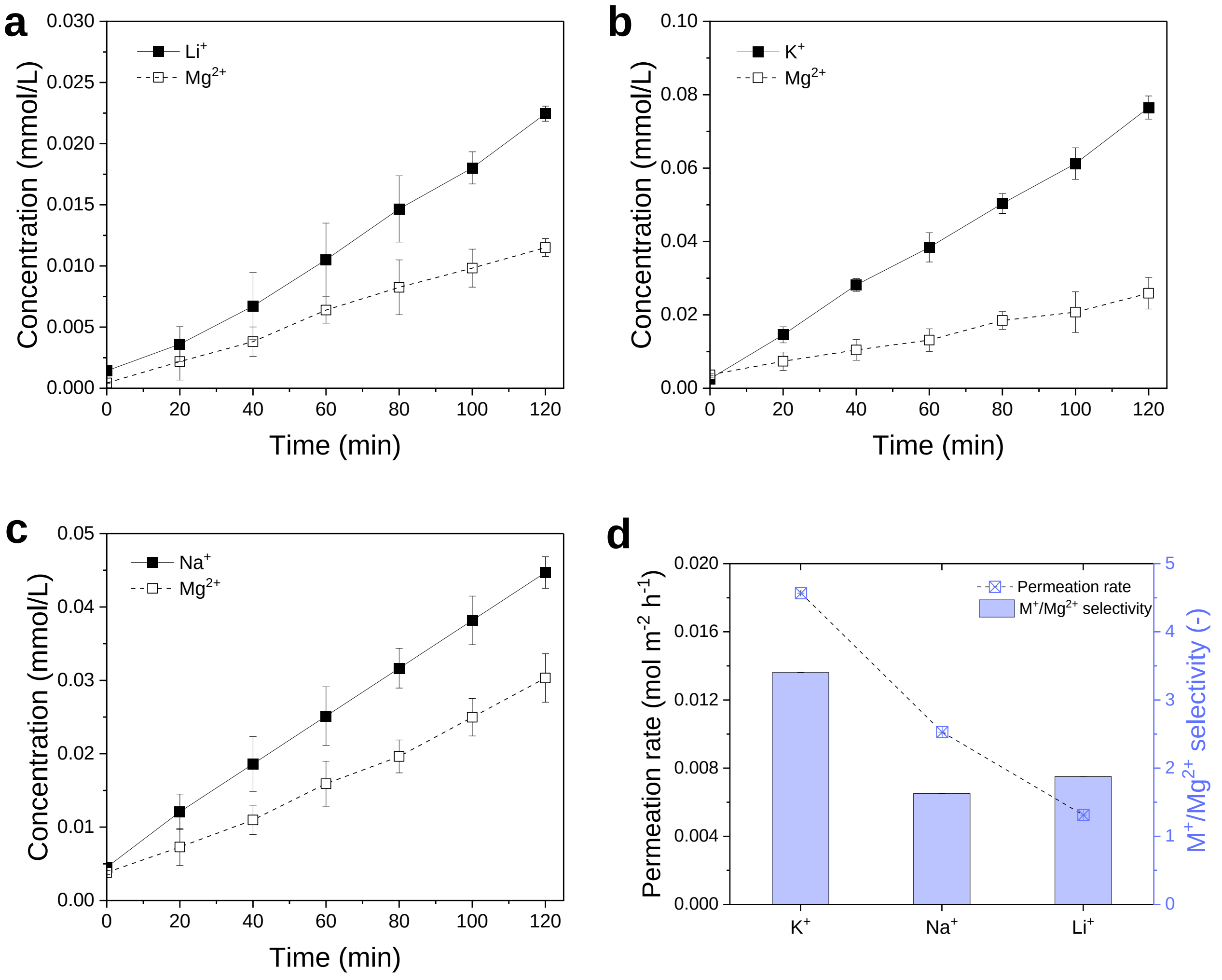

Supplementary Figure S46. Electrodialysis separation of binary mixtures through PIM-SBI-OMe-CN membrane. **a**, Concentration profiles of Li^+ and Mg^{2+} in concentrate chamber as a function of time ($LiCl$-$MgCl_2$ binary solution). **b**, Concentration profiles of K^+ and Mg^{2+} in concentrate chamber as a function of time (KCl-$MgCl_2$ binary solution). **c**, Concentration profiles of Na^+ and Mg^{2+} in concentrate chamber as a function of time ($NaCl$-$MgCl_2$ binary solution). **d**, The permeation rate and selectivity of PIM-SBI-OMe-CN for ion separation. In **a-c**, the data are presented as the mean ± s.d. (n=3) and the error bars represent the standard deviation (s.d.). In **d**, the error bars of permeation rate data represent the standard errors derived from linear fittings of salt concentration profiles of three independent experiments, and the error bars of selectivity data represent uncertainties derived from the permeation rates.

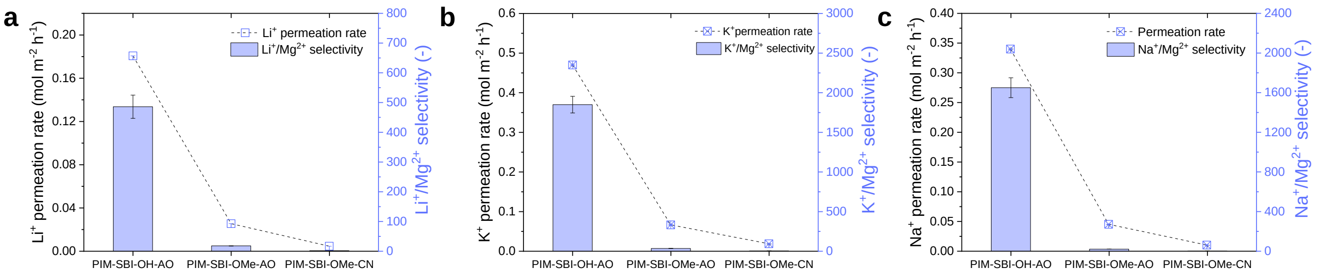

Supplementary Figure S47. Comparison of permeation rate and selectivity of ether-free PIM membranes for ion separation. a, Comparison of permeation rate and selectivity of PIM-SBI-OH-AO, PIM-SBI-OMe-AO, and PIM-SBI-OMe-CN for Li/Mg separation. **b**, Comparison of permeation rate and selectivity of PIM-SBI-OH-AO, PIM-SBI-OMe-AO, and PIM-SBI-OMe-CN for K/Mg separation. **c**, Comparison of permeation rate and selectivity of PIM-SBI-OH-AO, PIM-SBI-OMe-AO, and PIM-SBI-OMe-CN for Na/Mg separation. The error bars of permeation rate data represent the standard errors derived from linear fittings of salt concentration profiles of three independent experiments, and the error bars of selectivity data represent uncertainties derived from the permeation rates.

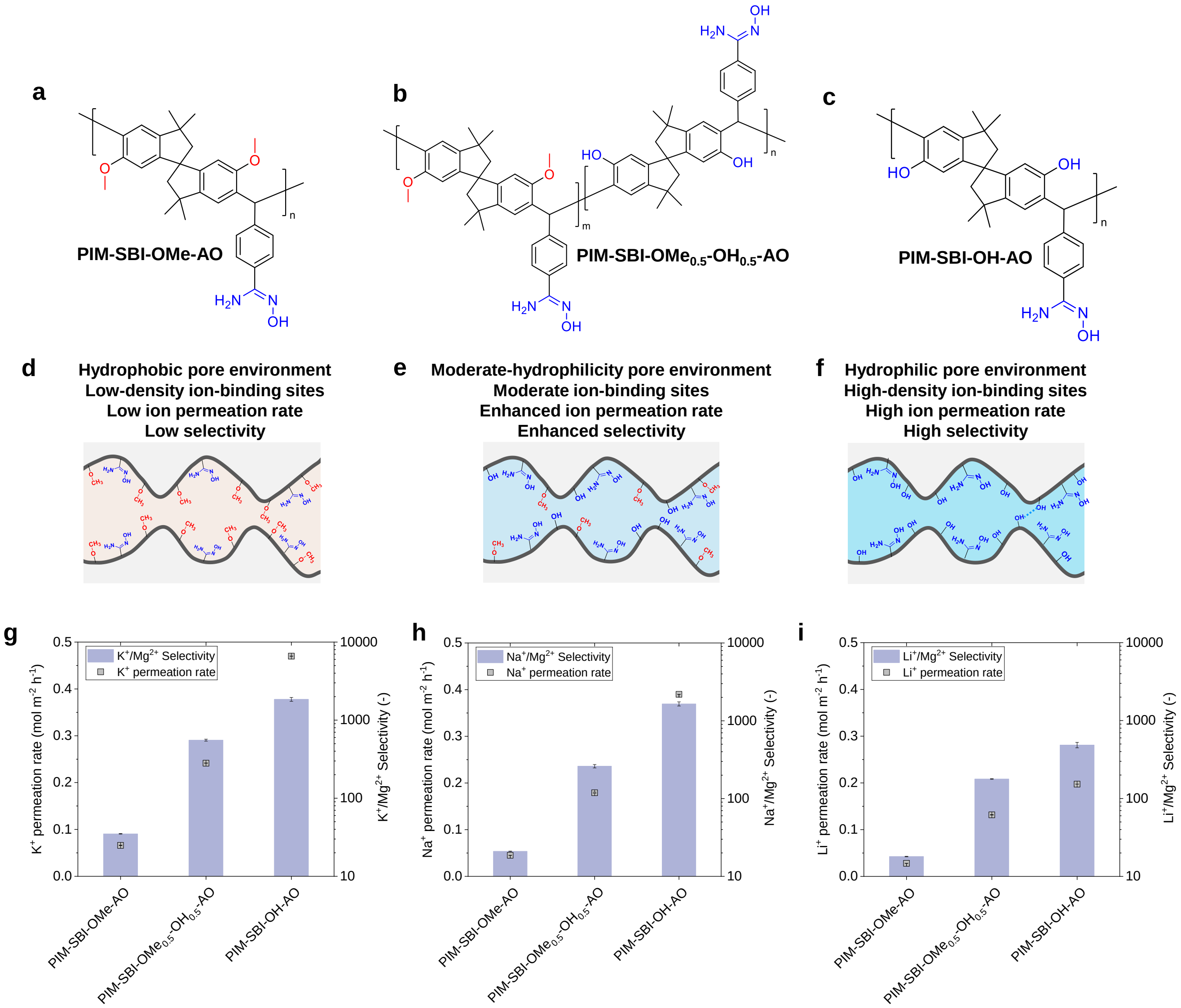

Supplementary Figure S48. Electrodialysis performance of AO-PIM membranes with varied functional groups. a, Structures of PIM-SBI-OMe-AO, **b**, PIM-SBI-OMe$_{0.5}$-OH$_{0.5}$-AO, and **c**, PIM-SBI-OH-AO. d, Schematic diagram showing the relatively hydrophobic pore environment of PIM-SBI-OMe-AO**. e**, Schematic diagram showing the moderate-hydrophilicity pore environment of PIM-SBI-OMe$_{0.5}$-OH$_{0.5}$-AO**. f**, Schematic diagram showing the hydrophilic pore environment of PIM-SBI-OH-AO**. g**, Comparison of permeation rate and selectivity of PIM-SBI-OH-AO, PIM-SBI-OMe-AO, and PIM-SBI-OMe-CN for separation of KCl-$MgCl_2$ binary solution. **h**, Comparison of permeation rate and selectivity of PIM-SBI-OMe-AO, PIM-SBI-OMe$_{0.5}$-OH$_{0.5}$-AO, and PIM-SBI-OH-AO for separation of NaCl-$MgCl_2$ binary solution. **i**, Comparison of permeation rate and selectivity of PIM-SBI-OMe-AO, PIM-SBI-OMe$_{0.5}$-OH$_{0.5}$-AO, and PIM-SBI-OH-AO for separation of LiCl-$MgCl_2$ binary solution. In **g-i**, the error bars of permeation rate data represent the standard errors derived from linear fittings of salt concentration profiles of three independent experiments, and the error bars of selectivity data represent uncertainties derived from the permeation rates.

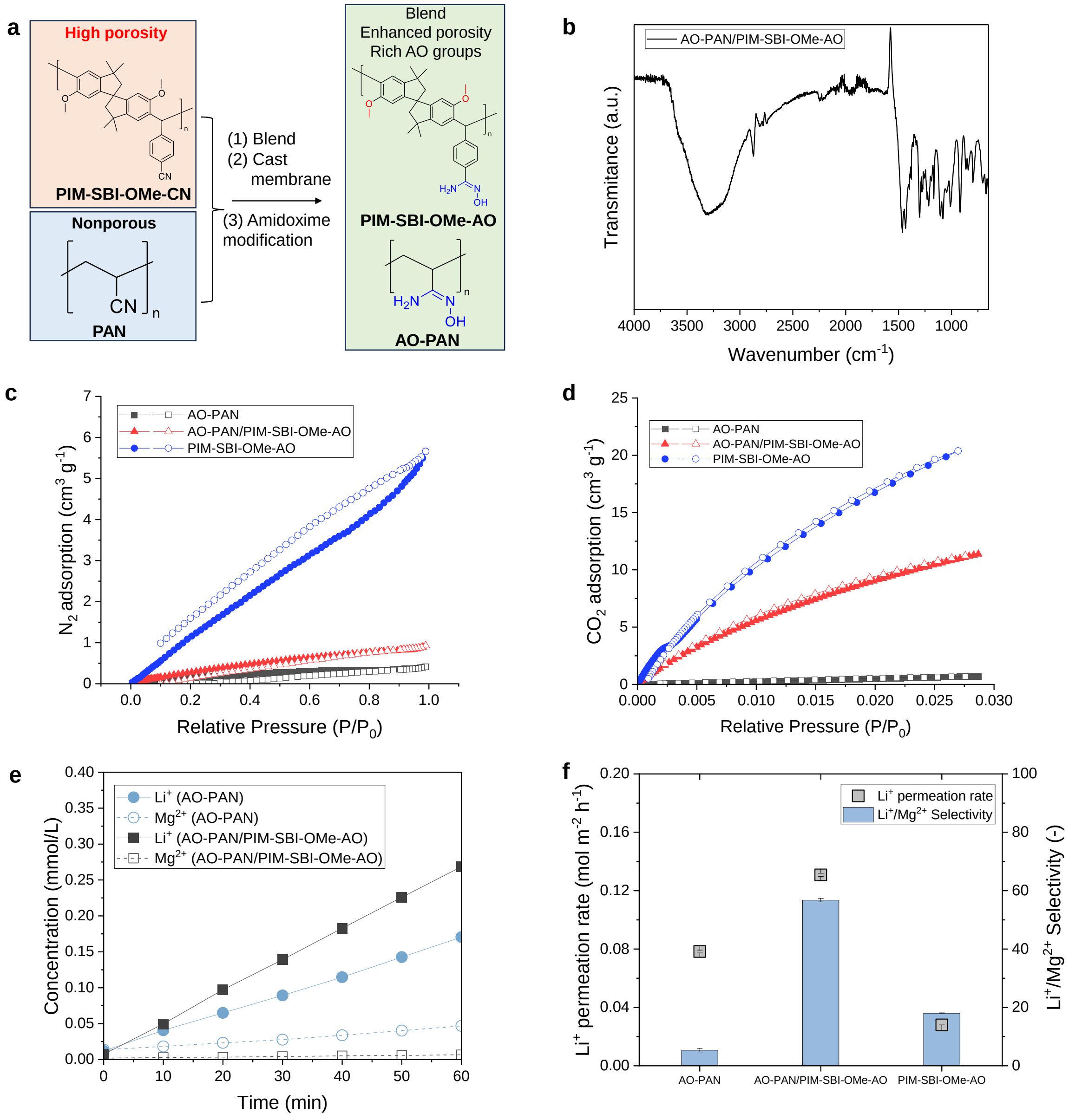

Supplementary Figure S49. Electrodialysis separation performance of blend membranes from AO-PAN and AO-PIM polymer. a, Structures of PIM-SBI-OMe-CN and PAN and modification to AO polymers. Blended AO-PAN/PIM-SBI-OMe-AO membranes were made through AO modification of blended PAN/PIM-SBI-OMe-CN membranes. PAN and PIM-SBI-OMe-CN polymers were blended with 1:1 mass ratio and dissolved in DMSO solvent. After fully stirred, the blended polymer solution was cast into membrane. Dried PAN/PIM-SBI-OMe-AO membrane samples were immersed in 50 mL of an aqueous hydroxylamine solution with concentration of 5 g L^{-1} at 60°C under N_2 for 3h. After the reaction, the membranes were thoroughly washed multiple times with deionized water. **b,** FTIR spectrum of blend membrane. **c**, N_2 adsorption of AO-PAN, AO-PAN/PIM-SBI-OMe-AO, and PIM-SBI-OMe-AO. **d**, CO_2 adsorption of AO-PAN, AO-PAN/PIM-SBI-OMe-AO, and PIM-SBI-OMe-AO. **e**, Ion permeation profiles. **f**, Comparison of permeation rate and selectivity of AO-PAN, AO-PAN/PIM-SBI-OMe-AO, and PIM-SBI-OMe-AO for Li/Mg separation. In **f**, the error bars of permeation rate data represent the standard errors derived from linear fittings of salt concentration profiles, and the error bars of selectivity data represent uncertainties derived from the permeation rates.

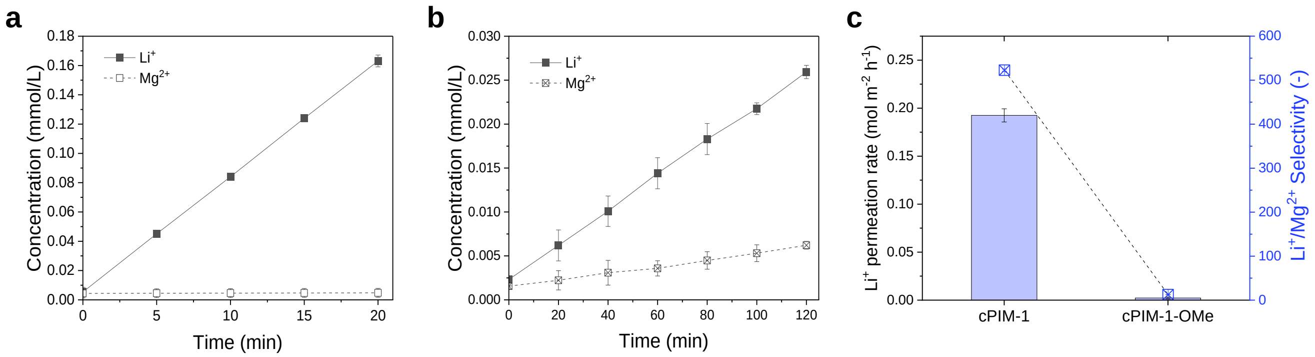

Supplementary Figure S50. Comparison of electrodialysis separation performance of cPIM-1 and cPIM-1-OMe membranes. a, Concentration profiles of Li^+ and Mg^{2+} in concentrate chamber for cPIM-1 membrane as a function of time. **b**, Concentration profiles of Li^+ and Mg^{2+} in concentrate chamber for cPIM-1-OMe membrane as a function of time. **c**, Permeation rate and selectivity of cPIM-1 and cPIM-1-OMe for Li/Mg separation. In **a-b**, the data are presented as the mean ± s.d. (n=3) and the error bars represent the standard deviation (s.d.). In **c**, the error bars of permeation rate data represent the standard errors derived from linear fittings of average salt concentration profiles of three independent experiments, and the error bars of selectivity data represent uncertainties derived from the permeation rates.

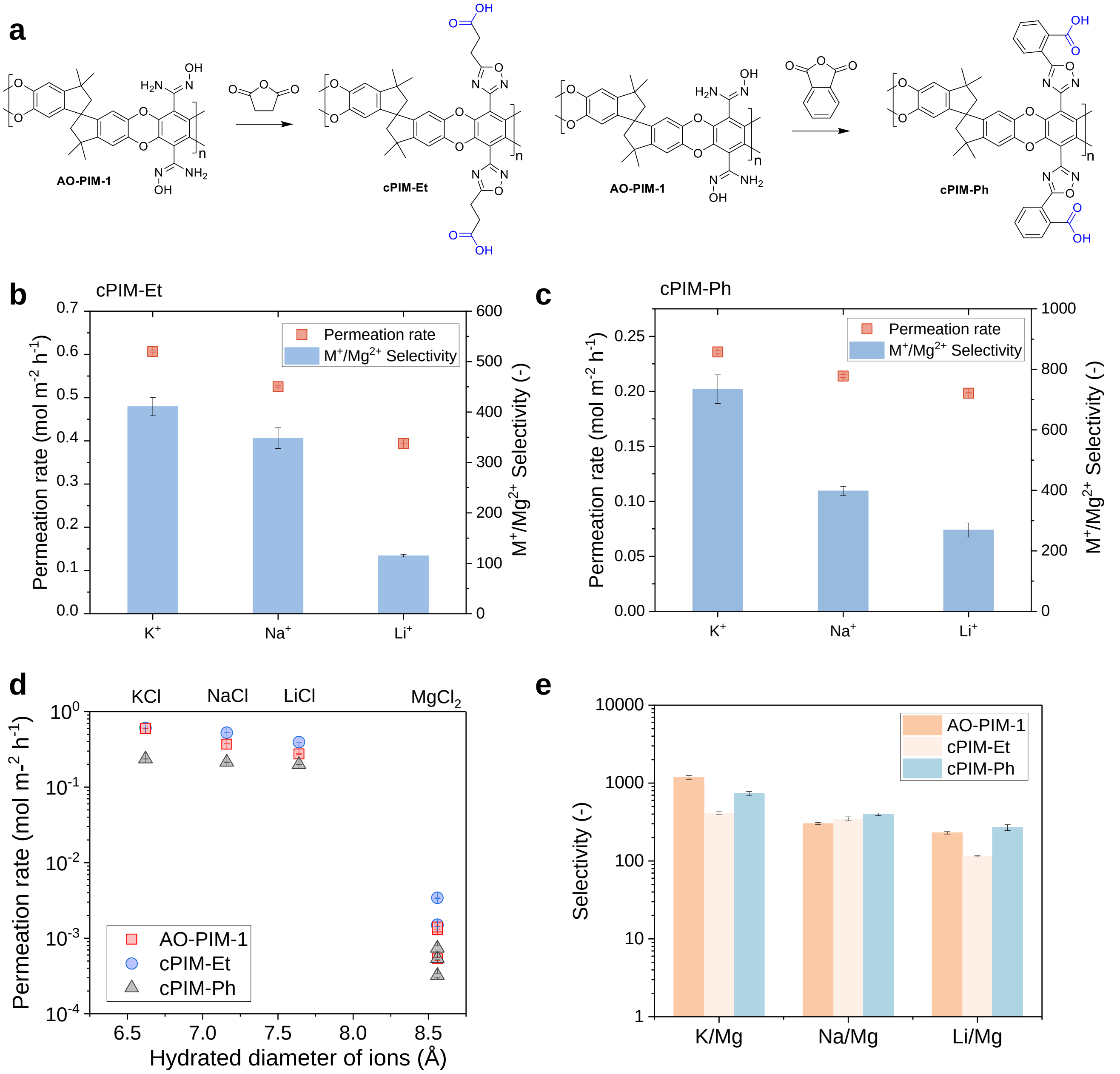

Supplementary Figure S51. Electrodialysis separation performance of cPIM membranes with pendant groups. a, Structures of cPIMs modified from AO-PIM-1, with varied pendant groups[7]. **b**, Ion permeation rate and selectivity of cPIM-Et for separation of K/Mg, Na/Mg, Li/Mg binary mixtures. **c**, Ion permeation rate and selectivity of cPIM-Ph for separation of K/Mg, Na/Mg, Li/Mg binary mixtures. **d**, Comparison of permeation rates of AO-PIM-1, cPIM-Et, and cPIM-Ph. **e**, Comparison of selectivity of AO-PIM-1, cPIM-Et, and cPIM-Ph. In **b-e**, the error bars of permeation rate data represent the standard errors derived from linear fittings of salt concentration profiles of three independent experiments, and the error bars of selectivity data represent uncertainties derived from the permeation rates.

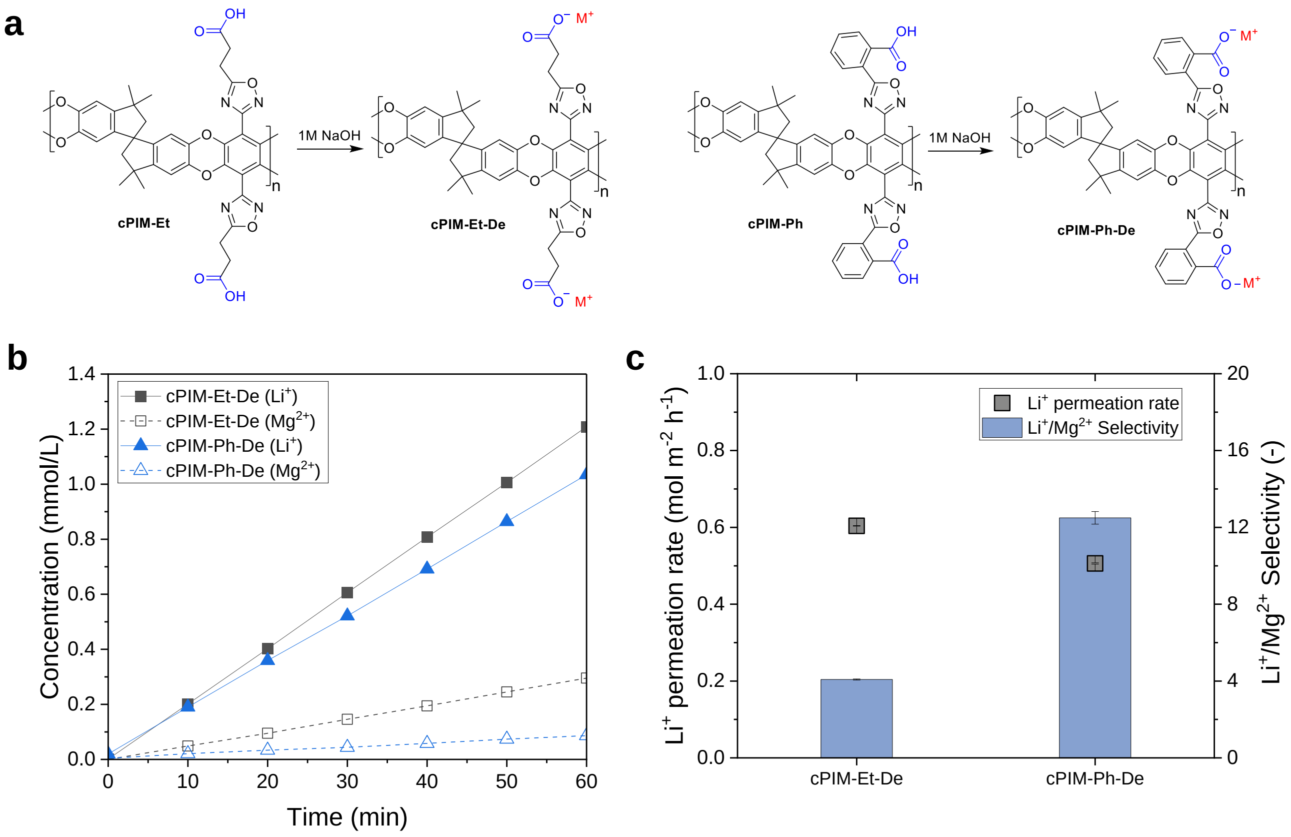

Supplementary Figure S52. Electrodialysis separation performance of ion-exchanged cPIM membranes. a, Ion exchange of cPIM membranes. **b**, Ion concentration profiles. **c**, Comparison of permeation rates and selectivity of cPIM-Et-De and cPIM-Ph-De. In **c**, the error bars of permeation rate data represent the standard errors derived from linear fittings of salt concentration profiles, and the error bars of selectivity data represent uncertainties derived from the permeation rates.

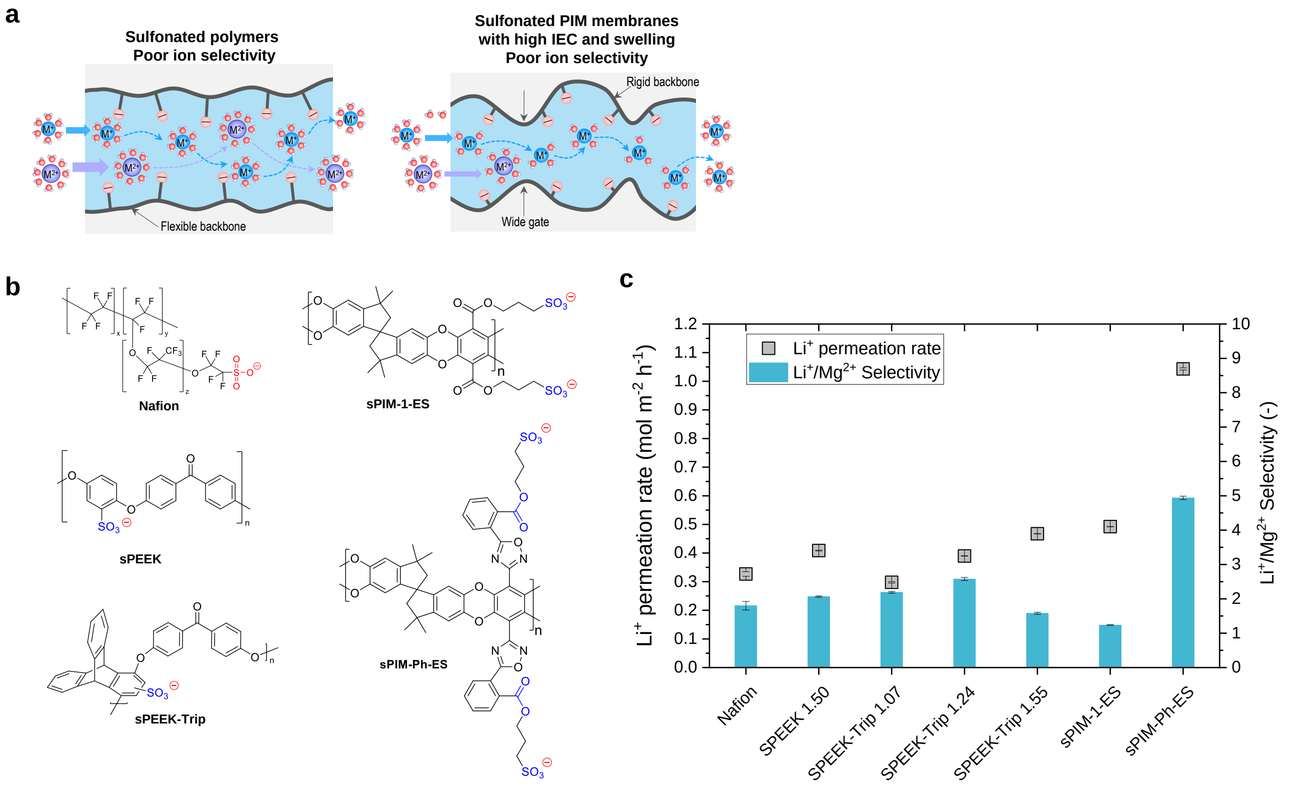

Supplementary Figure S53. Electrodialysis performance of sulfonated polymer membranes. a, Schematic diagram showing poor ion selectivity in cation exchange membranes. **b**, Structure of Nafion, sulfonated poly(ether-ether-ketone) (sPEEK) with ion exchange capacity of 1.5 meq g^{-1}, and sPEEK with rigid and contorted triptycene in the backbone (sPEEK-Trip) with varied ion exchange capacity of 1.07, 1.24, and 1.55 meq g^{-1} prepared following our recent work[25], sulfonated sPIM-1-ES, and sPIM-Ph-ES. **c**, Electrodialysis separation performance of membranes with Li/Mg binary mixture. All membranes were tested in a small ED stack with an effective area of 2 cm^2, and concentrate volume of 100 mL. In **c**, the error bars of permeation rate data represent the standard errors derived from linear fittings of salt concentration profiles of three independent experiments, and the error bars of selectivity data represent uncertainties derived from the permeation rates. Ionizable functional groups, such as sulfonate groups, could introduce strong electrostatic charges into the membrane pores, which promote the migration of divalent ions under the driving force of electric field, leading to poor mono/divalent selectivity.

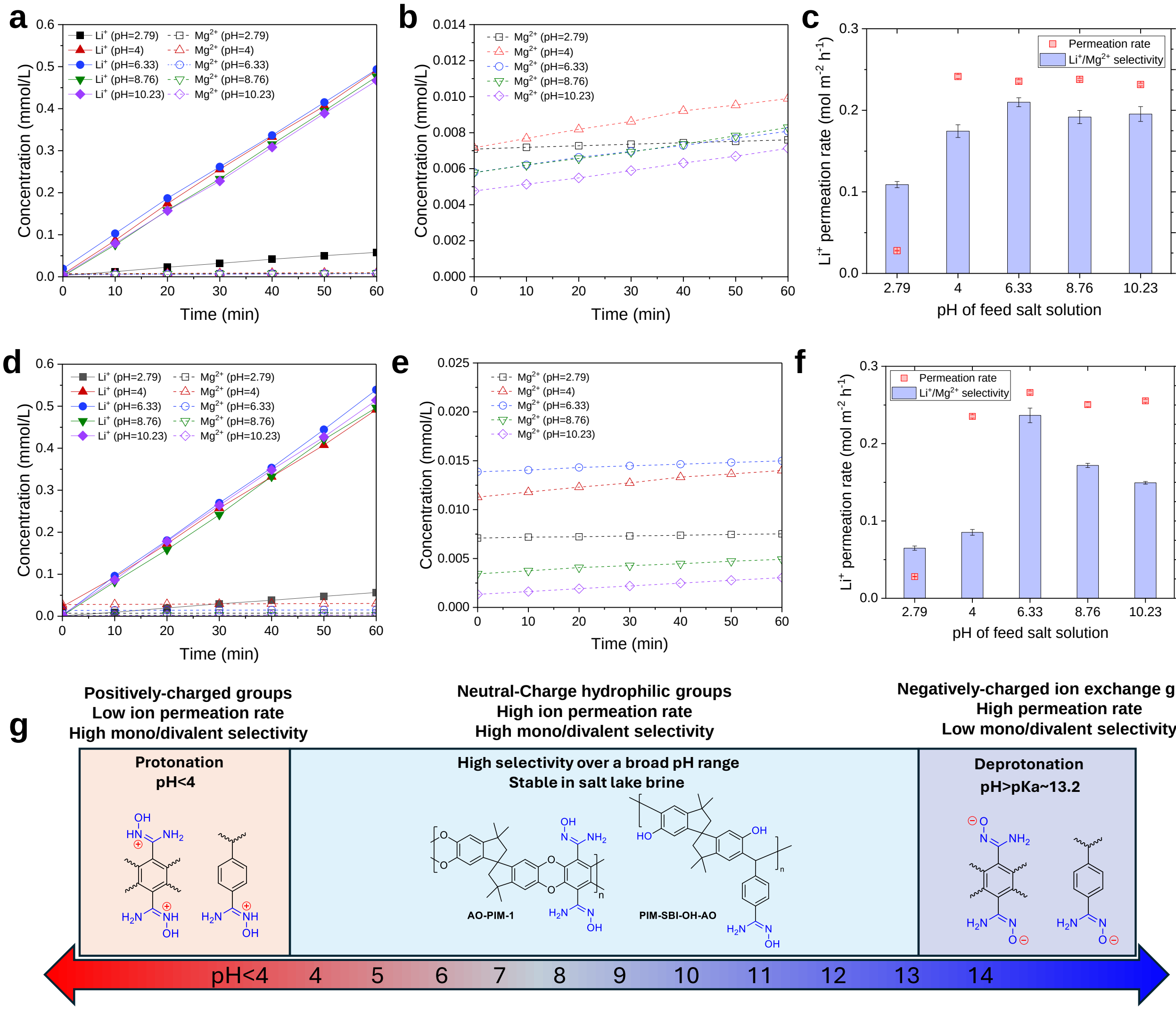

Supplementary Figure S54. Ion separation performance of AO-PIM-1 and PIM-SBI-OH-AO membranes in feed solutions with varied pH values. a, Concentration profiles of Li^+ and Mg^{2+} in concentrate chamber for AO-PIM-1 membrane as a function of time. **b**, Concentration profiles of Mg^{2+} in concentrate chamber for AO-PIM-1 membrane as a function of time. **c**, Li^+ ion permeation rate and Li^+/Mg^{2+} selectivity of AO-PIM-1 as a function of pH of feed solution. **d**, Concentration profiles of Li^+ and Mg^{2+} in concentrate chamber for PIM-SBI-OH-AO membrane as a function of time. **e**, Concentration profiles of Mg^{2+} in concentrate chamber for PIM-SBI-OH-AO membrane as a function of time. **f**, Li^+ ion permeation rate and Li^+/Mg^{2+} selectivity of PIM-SBI-OH-AO as a function of pH of feed solution. **g**, Proposed pH operation window for AO-PIM membranes in salt solution. At low pH (<4), the amidoxime groups become positively charged due to protonation, which led to electrostatic repulsion towards positively charged alkali metal cations (Li^+ and Mg^{2+}) and consequently lower permeation rates, while maintaining high selectivity (>100). At higher pH (>13), the amidoxime groups will be deprotonated and become negatively charged, which facilitates the transport of Mg^{2+} ion, leading to lower selectivity. Therefore, the AO-PIM membranes are stable over a broad pH range, especially for salt lake brine (the pH is typically within 7-11). In **c** and **f**, the error bars of permeation rate data represent the standard errors derived from linear fittings of salt concentration profiles, and the error bars of selectivity data represent uncertainties derived from the permeation rates.

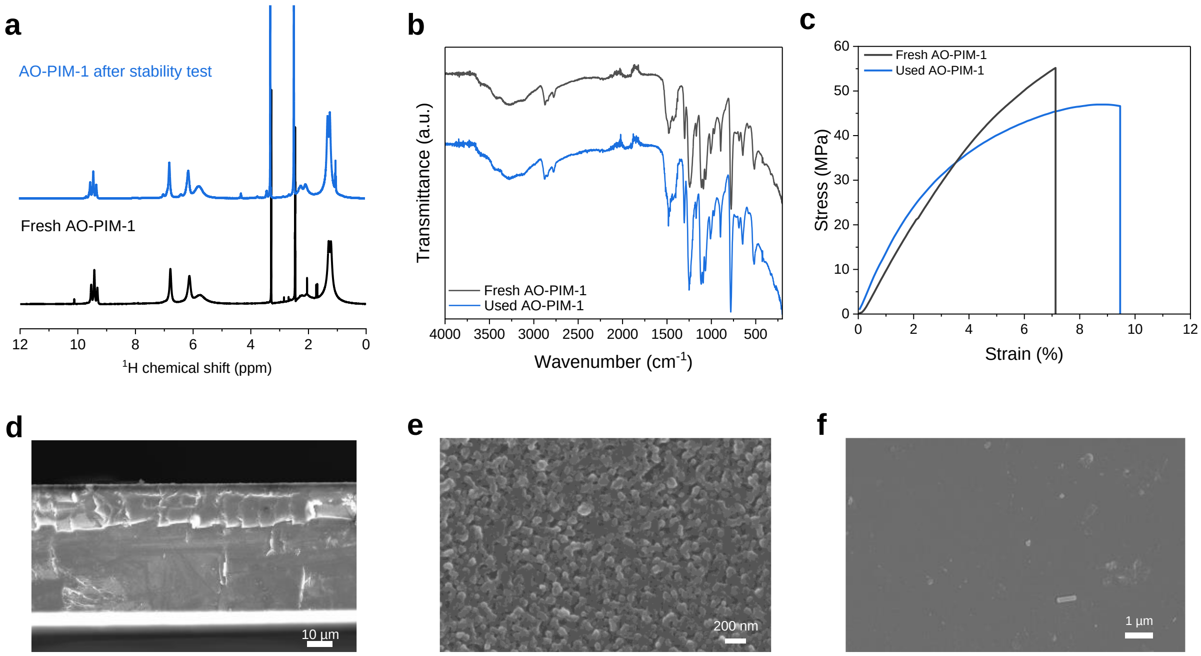

Supplementary Figure S55. Characterization of fresh and used AO-PIM-1 membranes. a, NMR spectra. **b**, FTIR spectra. **c**, Tensile strength test profiles. d, Cross-sectional SEM image of used AO-PIM-1. **e**, Cross-sectional SEM image of used AO-PIM-1 at high magnification. **f**, Surface SEM image of AO-PIM-1 membrane. The used AO-PIM-1 membrane showed good chemical and mechanical stability.

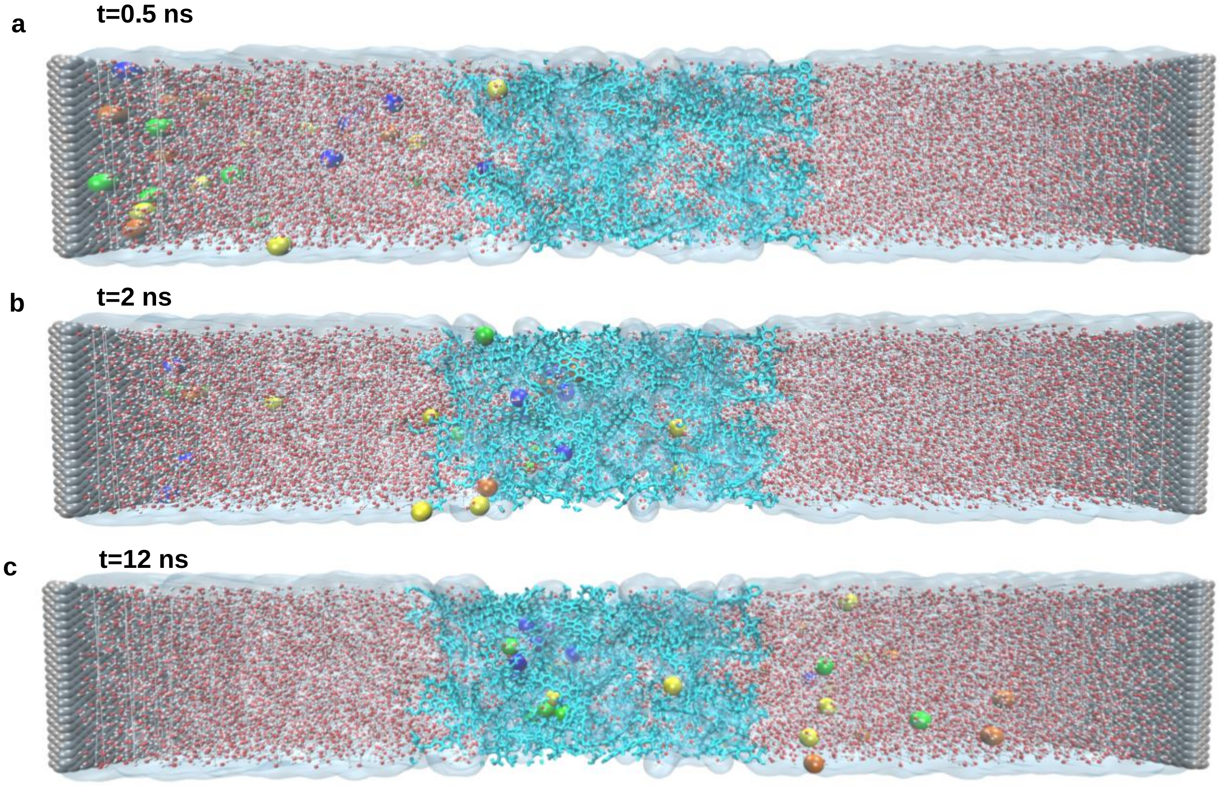

Supplementary Figure S56. Snapshots of non-equilibrium models of ion transport through PIM membranes at different states. a, Initial state, t=0.5 ns; **b**, Ions entering the membrane nanochannels, t=2 ns; **c**, Ions passing through the membranes, t=12 ns.

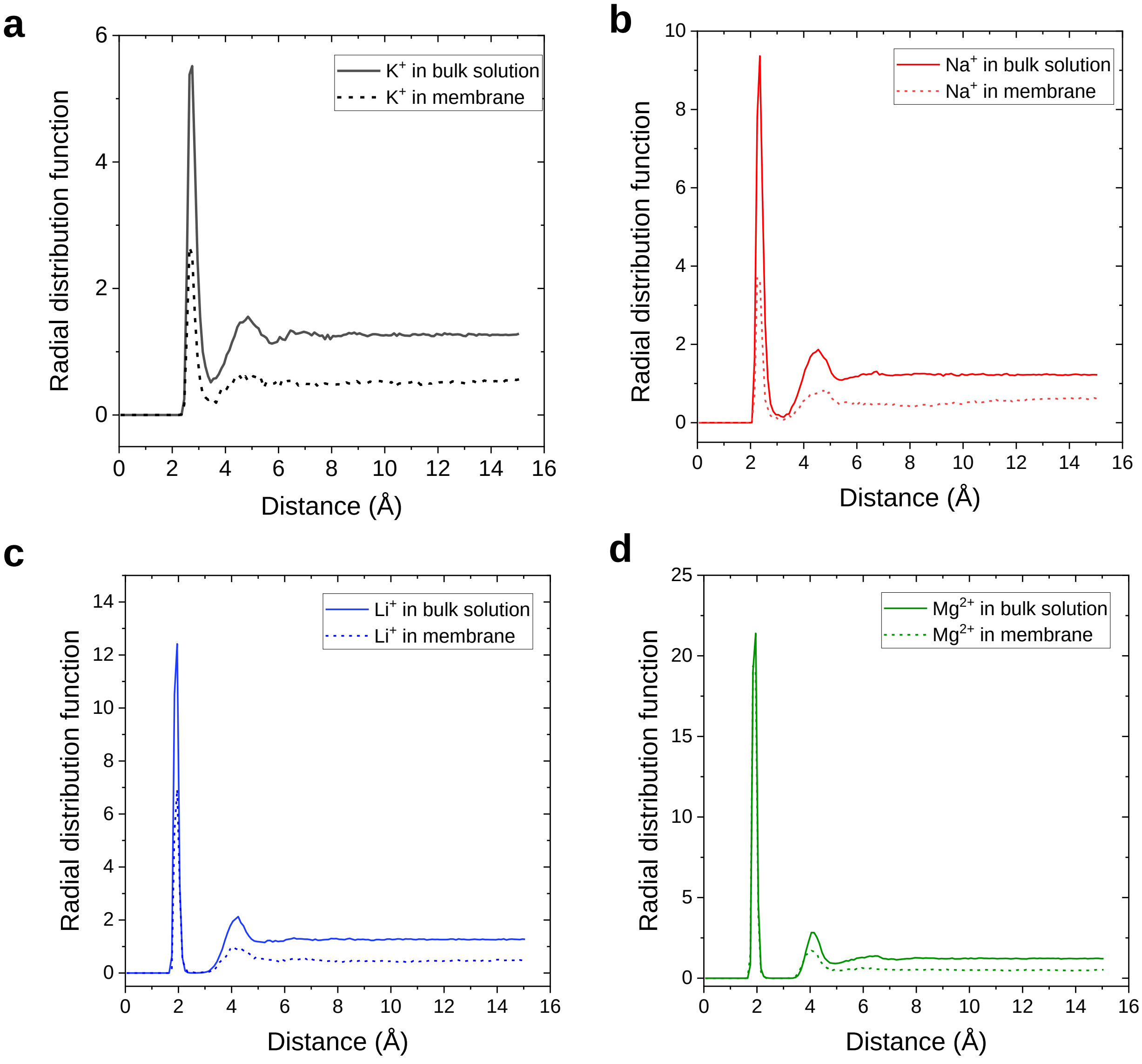

Supplementary Figure S57. RDF of water molecules as function of the distance from the center of ions in bulk solution and in membrane. **a**, K^+; **b**, Na^+; **c**, Li^+; and **d**, Mg^{2+}. There is a clear boundary between the first and second hydration shells for Mg and Li ions, indicating the strong bonding of water molecules. For K and Na ions, the boundary between the first and the second hydration layers are quite close, which is due to the weak bonding and the water molecules can exchange between the two layers.

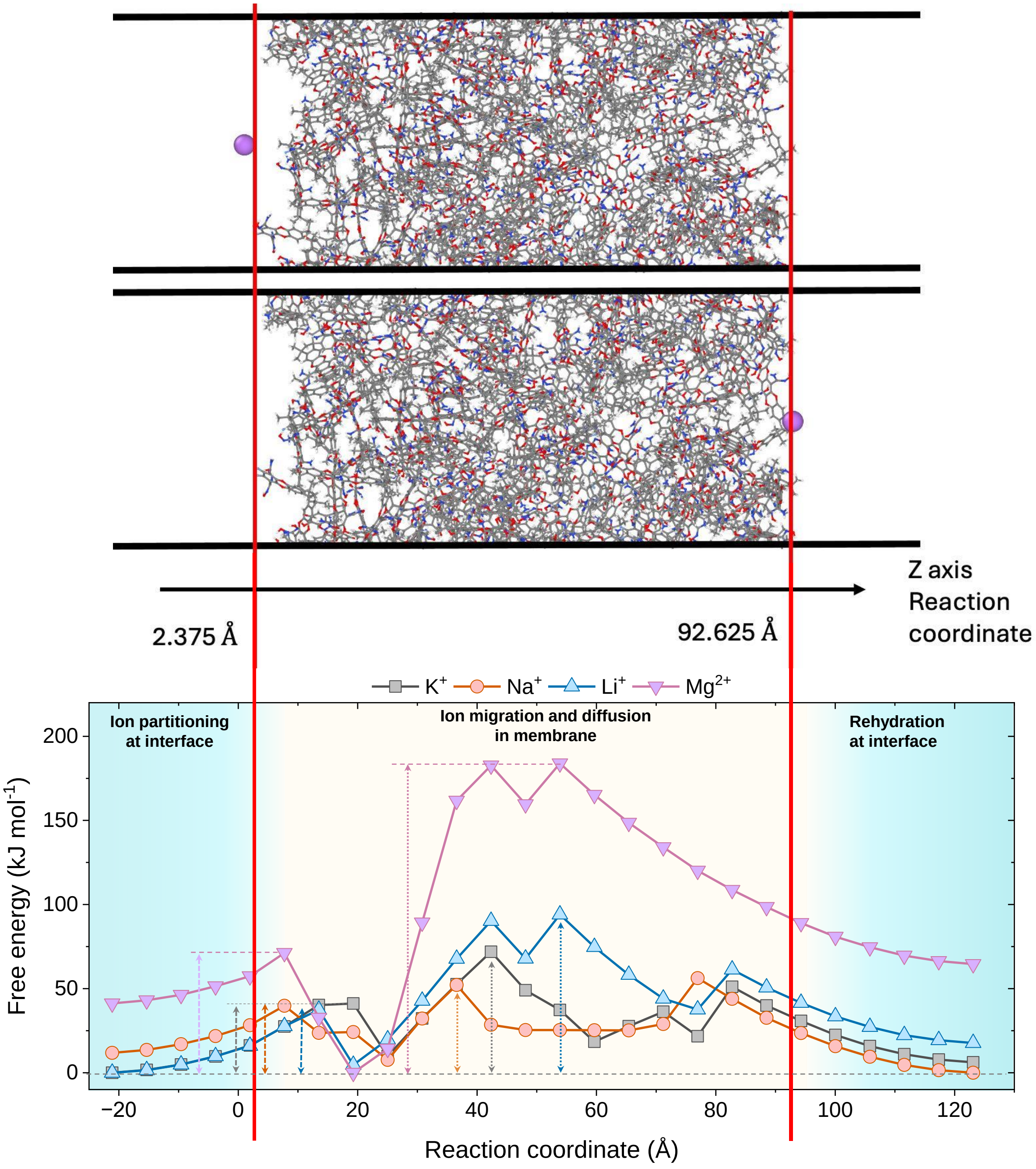

Supplementary Figure S58. Energy barrier for salt ion transport through the subnanometer pores in the AO-PIM-1 membrane. Short dashed arrows indicate the energy barriers for ion partitioning, and dashed arrows indicate the energy barriers for ion diffusion in the membrane.

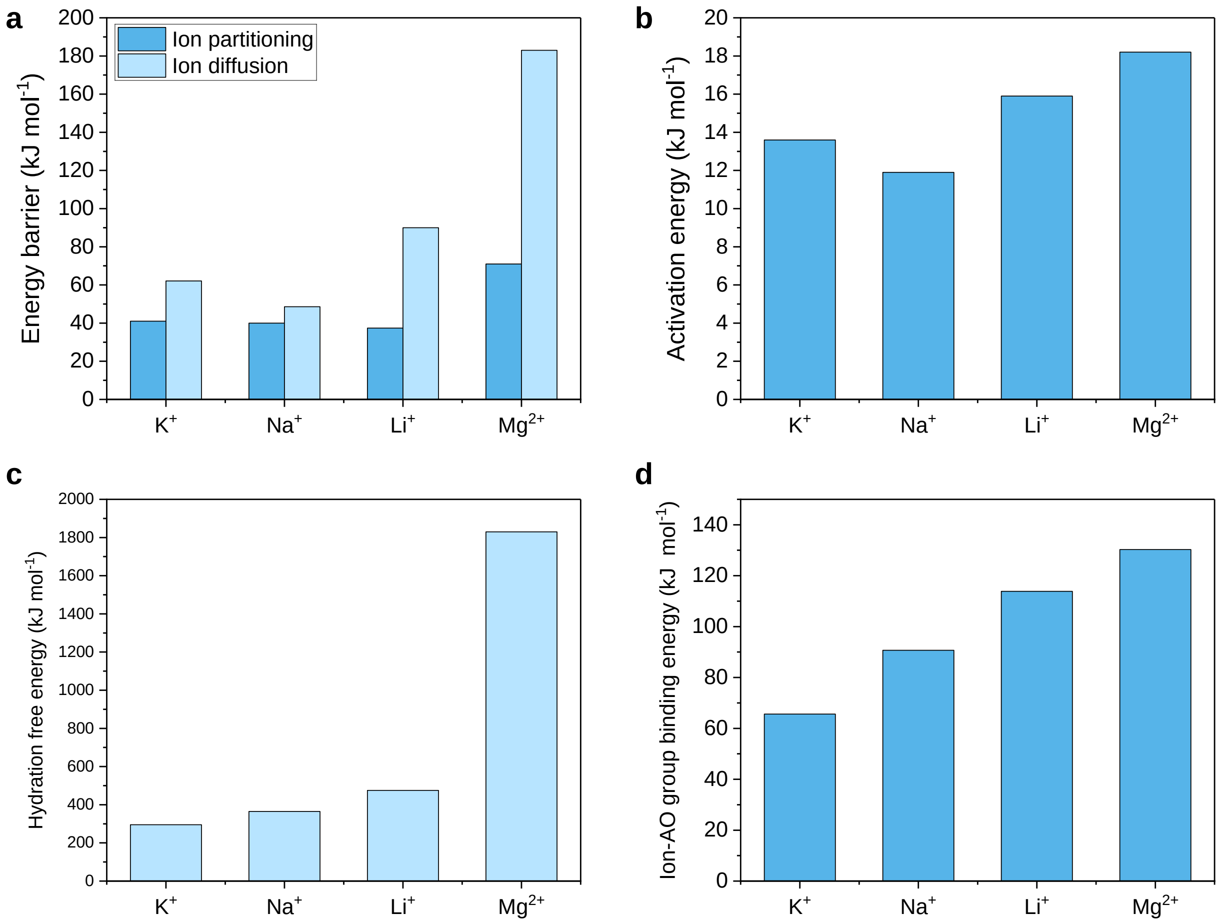

Supplementary Figure S59. Energy barrier analysis. a, Energy barriers calculated by non-equilibrium model. **b,** Activation energy measured by conductivity. **c**, Ion hydration energy. **d,** Ion-AO group binding energy derived by DFT calculation.

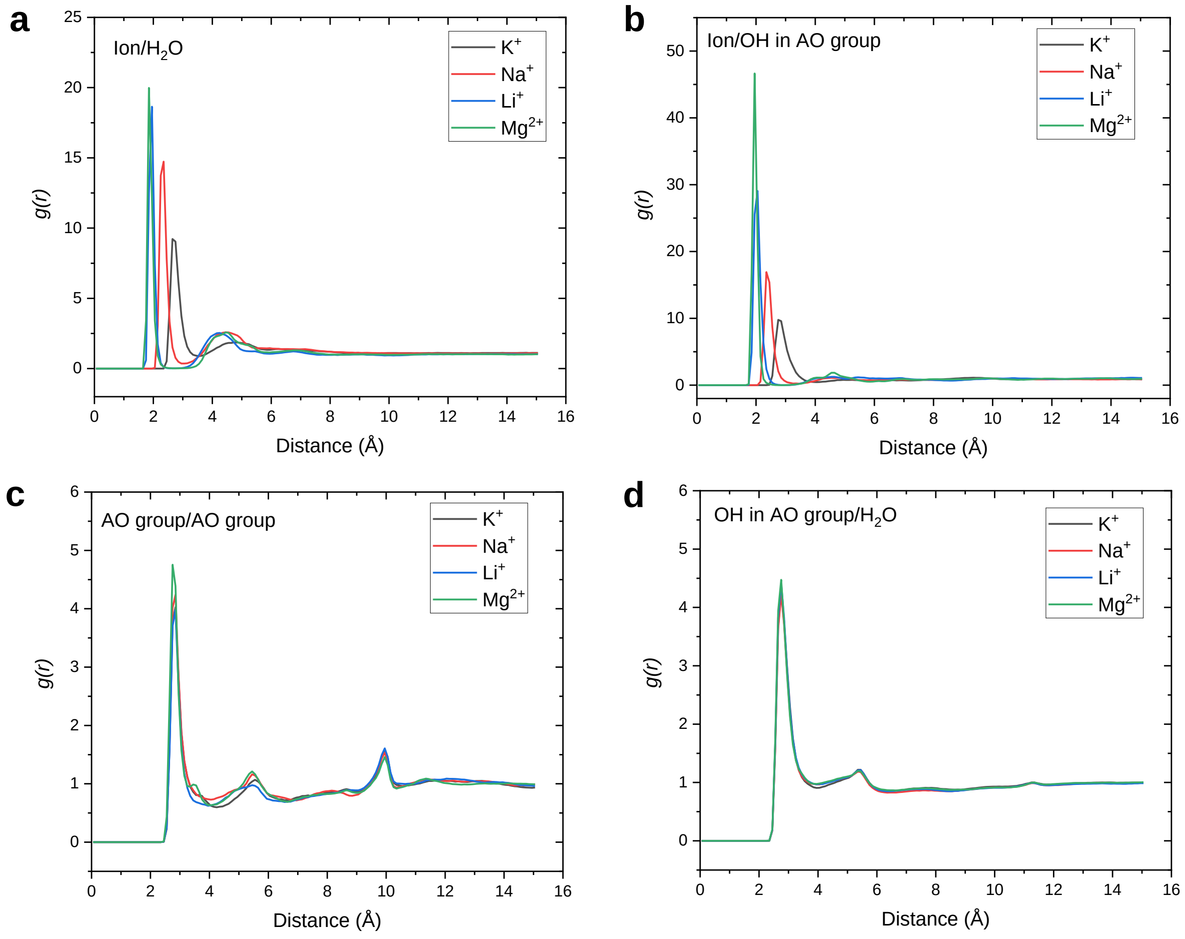

Supplementary Figure S60. Radial distribution function (RDF) plots. a, RDF as a function of the distance between ions and water molecules. **b**, RDF as a function of the distance between ions with hydroxyl groups in amidoxime groups. **c**, RDF as a function of the distance between amidoxime groups. **d**, RDF as a function of the distance between hydroxyl groups in amidoxime with water molecules.

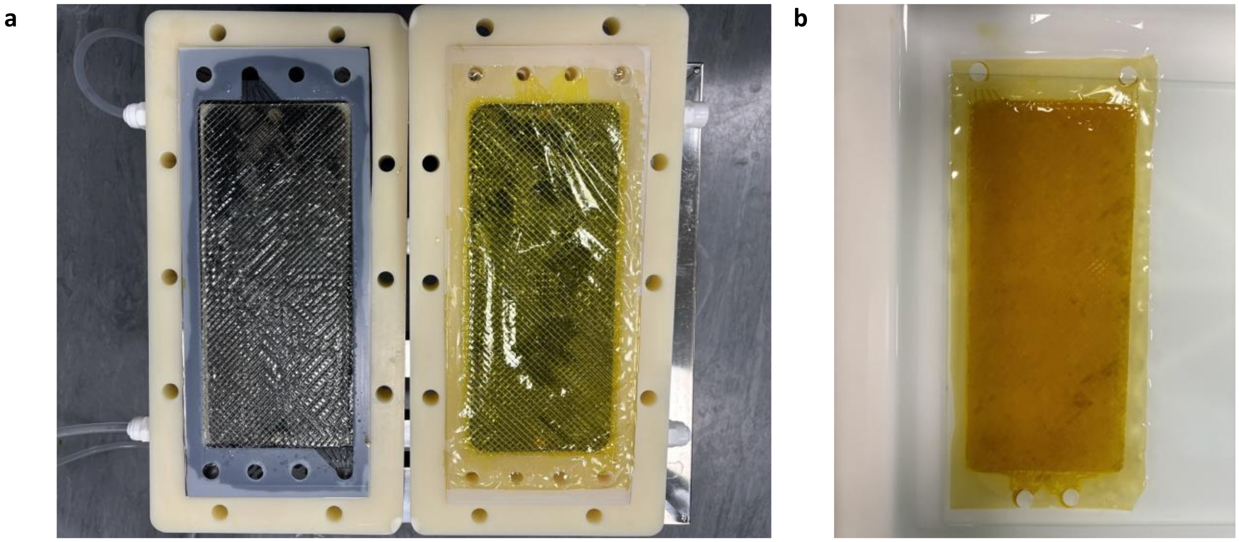

Supplementary Figure S61. Photos of membranes. a, Fresh AO-PIM-1 membrane and **b**, Recovered AO-PIM-1 membrane after testing in the electrodialysis stack. The membrane was still mechanically robust after testing in the membrane stack.

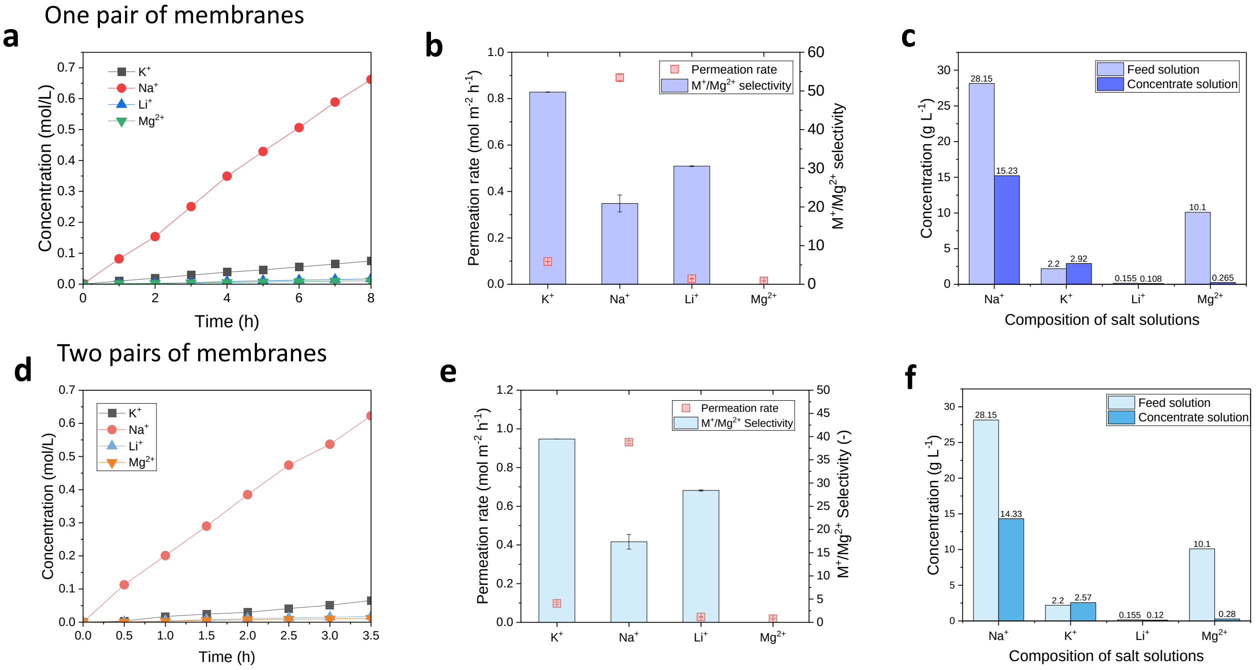

Supplementary Figure S62. Electrodialysis separation performance in large stacks with one pair (a-c) and two pairs of membranes (d-f). a, Ion concentration profiles of concentrate chamber, with feed solution of salt-lake reservoirs in China (diluted 2 times), and derived **b**, Ion permeation rates and selectivity. **c**, Compositions of initial feed solution and concentrate solution after testing. **d**, Ion concentration profiles of concentrate chamber, with feed solution of salt-lake reservoirs in China (diluted 2 times), and derived **e**, Ion permeation rates and selectivity. **f**, Compositions of initial feed solution and concentrate solution after testing. The composition of feed salt solution (hypersaline brine from salt-lake reservoirs) is presented in Supplementary Table S6. In **b** and **e**, the error bars of permeation rate data represent the standard errors derived from linear fittings of salt concentration profiles, and the error bars of selectivity data represent uncertainties derived from the permeation rates.

a

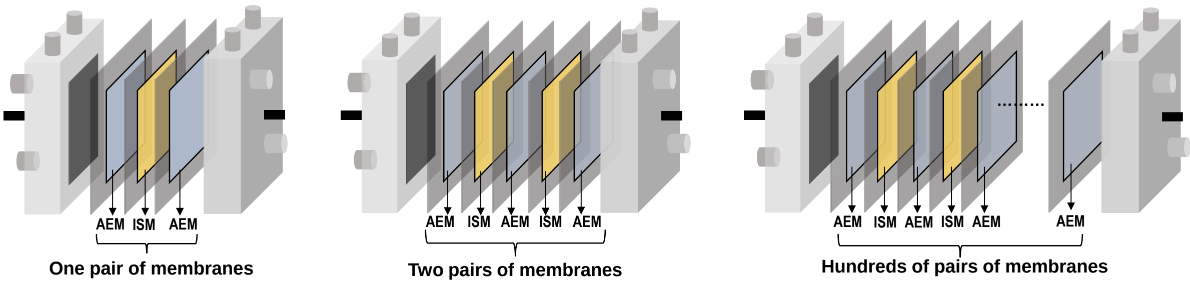

b

Li/Na/K/Mg=1/1/1/1 0.1 M mixed ion solution		
**Number of pairs**	**SEC_{Li} (kWh/mol)**	**SEC_{Li} (kWh/kg)**
One	0.138	19.87
Two	0.076	10.94

c

Typical Chinese salt lake brine (diluted two times)		
**Number of pairs**	**SEC_{Li} (kWh/mol)**	**SEC_{Li} (kWh/kg)**
One	0.7	100.79
Two	0.29	41.76

Supplementary Figure S63. Energy consumption analysis of electrodialysis separation performance in large stacks with one and two pairs of membranes. a, Schematic diagram of electrodialysis stack with one pair of membranes, two pairs of membranes, and multiple pairs of membranes. **b**, Energy consumption for lithium recovery from a salt mixture (KCl/NaCl/LiCl/$MgCl_2$, 0.1 M each). **c**, Energy consumption for lithium recovery from a typical Chinese salt lake brine solution (Composition shown in Supplementary Table S6).

As in the simulated brine solution contains very high concentrations of Na^+, K^+ and Mg^{2+} and low concentration of Li^+, the specific energy consumption for Lithium extraction with one pair of membranes is 100.79 kWh kg^{-1} and with two pairs of membranes is 41.76 kWh kg^{-1}. We acknowledge that the specific energy consumption for lithium extraction in this preliminary study is still high compared to other separation technologies, for example, Li et al [26] reported a membrane free electrochemical cell for lithium extraction and achieved quite low energy consumption for Li recovery (only 11.59 kWh kg^{-1} Li_2CO_3). However, in ED technology's real applications, hundreds of membranes can be assembled in the electrodialysis stack, which can greatly improve the voltage applied to the system and enhance overall ion transport efficiency. We envision that upscaling and optimization of the electrodialysis stack design would lead to lower specific energy consumption. Furthermore, with the development of renewable energy such as wind and solar power, there is potential to use renewable energy to drive electrodialysis processes, energy consumption can be further reduced.

Further work is required to test the membranes in electrodialysis modules and evaluate their efficiency and separation performance. Engineering efforts are necessary such as optimizing the configuration of membrane stacks, flow channels, and spacers to minimize resistance and maximize ion transport. Innovations in module design will lead to improved mass transfer rates, reduced energy consumption, and enhanced overall system performance.

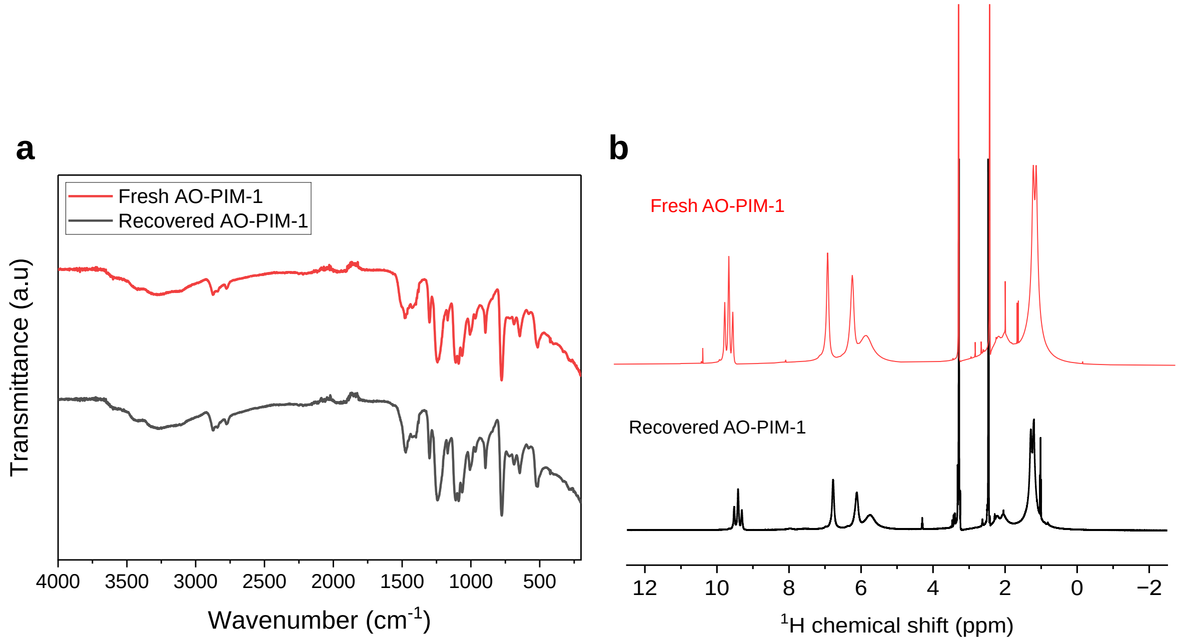

Supplementary Figure S64. Characterization of recovered membranes. a, Comparison of FTIR spectra of fresh AO-PIM-1 and recovered AO-PIM-1. **b**, Comparison of ^{1}H chemical shift of fresh AO-PIM-1 and recovered AO-PIM-1.

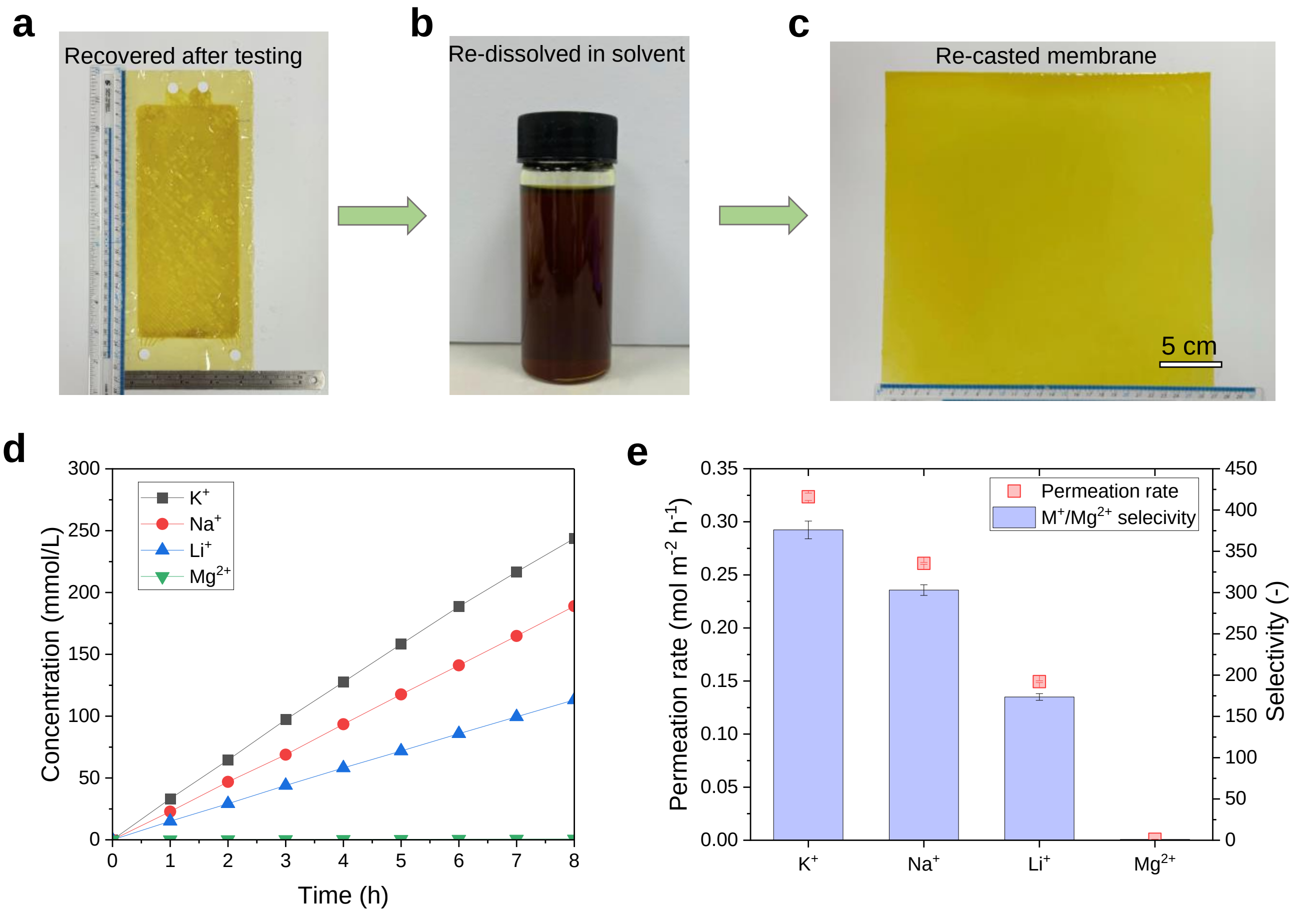

Supplementary Figure S65. Recyclability of membranes. a, Photo of AO-PIM-1 membrane recovered from the stack. **b**, Photo of redissolved polymer solution. **c**, Photo of one re-cast AO-PIM-1 membrane. **d**, Ion concentration profiles for concentrate chamber using recovered AO-PIM-1 membrane, with feed solution of $KCl/NaCl/LiCl/MgCl_2$ (0.1 M), and derived **e**, Ion permeation rates and selectivity. In **e**, the error bars of permeation rate data represent the standard errors derived from linear fittings of salt concentration profiles, and the error bars of selectivity data represent uncertainties derived from the permeation rates. AO-PIM-1 membranes can be cleaned, redissolved, and re-cast into defect-free membranes, maintaining high ion permeation rates and selectivity upon reuse. Recycling polymeric membranes has become an emerging approach to improve the sustainability of membrane processes[27]. Our preliminary result on recycling the membranes without significant loss of performance is a significant indicator of stability, and potentially offer economic and environmental benefits.

Supplementary Table S1. PFG-NMR results. T_1: The time constant for the loss of resonance intensity after a pulse excitation. T_2: The time constant for the width or broadness of resonances.

Samples	T_1 (s)	T_2 (ms)	D (m^2 s^{-1})
AO-PIM-1 hydrated with H_2O	0.199	10.6	1.31×10^{-10}
AO-PIM-1 hydrated with 0.1 M LiCl	0.185	11.5	1.21×10^{-10}
AO-PIM-1 hydrated with 0.1 M $MgCl_2$	0.195	9.54	1.33×10^{-10}
AO-PIM-1 hydrated with 0.1M LiCl+0.1M $MgCl_2$	0.207	7.95	1.40×10^{-10}
AO-PIM-De hydrated with H_2O	0.184	11.3	2.18×10^{-10}
AO-PIM-1-Et hydrated with H_2O	0.222	7.74	3.14×10^{-11}
Bulk DI water	2.831	2063	2.26×10^{-9}
Bulk solution 0.1M LiCl	2.818	2042	2.21×10^{-9}
Bulk solution 0.1M $MgCl_2$	2.761	1789	2.21×10^{-9}
Bulk solution 0.1M LiCl+0.1M $MgCl_2$	2.74	1760	2.23×10^{-9}

Supplementary Table S2. Basic parameters of cations.

Cations	Bare diameter (Å)	Hydrated diameter (Å)	Hydration free energy (kJ mol^{-1})
K^+	2.66	6.62	-295
Na^+	1.90	7.16	-365
Li^+	1.20	7.64	-475
Mg^{2+}	1.30	8.56	-1830

Supplementary Table S3. Operation conditions for electrodialysis tests.

**Chamber**	**Salt solutions (g L^{-1})**	**Volume (mL)**	**Flow rate (mL min^{-1})**
Feed	0.1 M mixed salt solution	100	40
Concentrate	0.01M salt solution	100	40
Electrode	Na_2SO_4	200	80

Supplementary Table S4. Summary of ion separation performance. Both concentration-driven diffusion dialysis and electrodialysis data are included.

Membrane	Ion permeation rate ($mol\cdot m^{-2}\cdot h^{-1}$)			Selectivity			Feed solution	Ref
	K^+	Na^+	Li^+	K^+/Mg^{2+}	Na^+/Mg^{2+}	Li^+/Mg^{2+}		
**AO-PIM-1 1mA cm^{-2}**	**0.29**	**0.24**	**0.13**	**1247**	**434**	**180**	**0.1 M mixed salt solution**	**This work**
**AO-PIM-1 1.5mA cm^{-2}**	**0.50**	**0.30**	**0.18**	**1928**	**246**	**212**	**0.1 M mixed salt solution**	**This work**
**AO-PIM-1 2mA cm^{-2}**	**0.63**	**0.37**	**0.28**	**1181**	**302**	**230**	**0.1 M mixed salt solution**	**This work**
**AO-PIM-1 2.5mA cm^{-2}**	**0.81**	**0.45**	**0.33**	**691**	**230**	**130**	**0.1 M mixed salt solution**	**This work**
**AO-PIM-1 3mA cm^{-2}**	**1.02**	**0.51**	**0.40**	**847**	**245**	**50**	**0.1 M mixed salt solution**	**This work**
**AO-PIM-1-De 2mA cm^{-2}**	**0.68**	**0.51**	**0.30**	**66**	**40**	**7**	**0.1 M mixed salt solution**	**This work**
**AO-PIM-1-Et 2mA cm^{-2}**	**0.024**	**0.011**	**0.006**	**99**	**61**	**30**	**0.1 M mixed salt solution**	**This work**
**PIM-SBI-OH-AO 2mA cm^{-2}**	**0.47**	**0.39**	**0.20**	**1850**	**1650**	**486**	**0.1 M mixed salt solution**	**This work**
**PIM-SBI-$OMe_{0.5}$-$OH_{0.5}$-AO 2mA cm^{-2}**	**0.24**	**0.18**	**0.13**	**555**	**261**	**178**	**0.1 M mixed salt solution**	**This work**
**PIM-SBI-OMe-AO 2mA cm^{-2}**	**0.066**	**0.045**	**0.028**	**35**	**21**	**18**	**0.1 M mixed salt solution**	**This work**
**PIM-SBI-OMe-CN 2mA cm^{-2}**	**0.0183**	**0.0101**	**0.0052**	**3.4**	**1.6**	**1.9**	**0.1 M mixed salt solution**	**This work**
**cPIM-1 2mA cm^{-2}**	****	****	**0.24**	****	****	**420**	**0.1 M mixed salt solution**	**This work**
**cPIM-Et 2mA cm^{-2}**	**0.61**	**0.53**	**0.39**	**411**	**348**	**115**	**0.1 M mixed salt solution**	**This work**
**cPIM-Et-De 2mA cm^{-2}**	****	****	**0.60**	****	****	**4.1**	**0.1 M mixed salt solution**	**This work**
**cPIM-Ph 2mA cm^{-2}**	**0.24**	**0.21**	**0.20**	**735**	**398**	**269**	**0.1 M mixed salt solution**	**This work**
**cPIM-Ph-De 2mA cm^{-2}**	****	****	**0.51**	****	****	**12.5**	**0.1 M mixed salt solution**	**This work**
**cPIM-OMe 2mA cm^{-2}**	****	****	**0.006**	****	****	**5**	**0.1 M mixed salt solution**	**This work**
**sPEEK-Trip-1.07 2mA cm^{-2}**	****	****	**0.30**	****	****	**2.2**	**0.1 M mixed salt solution**	**This work**
**sPEEK-Trip-1.24 2mA cm^{-2}**	****	****	**0.39**	****	****	**2.6**	**0.1 M mixed salt solution**	**This work**
**sPEEK-Trip-1.55 2mA cm^{-2}**	****	****	**0.48**	****	****	**1.6**	**0.1 M mixed salt solution**	**This work**
**Nafion 212 2mA cm^{-2}**	****	****	**0.33**	****	****	**1.8**	**0.1 M mixed salt solution**	**This work**
**sPIM-1-ES 2mA cm^{-2}**	****	****	**0.49**	****	****	**1.2**	**0.1 M mixed salt solution**	**This work**
**sPIM-Ph-ES 2mA cm^{-2}**	****	****	**1.04**	****	****	**4.9**	**0.1 M mixed salt solution**	**This work**
**AO-PAN 2mA cm^{-2}**	**0.16**	**0.12**	**0.08**	**53**	**40**	**5.3**	**0.1 M mixed salt solution**	**This work**
COF-EO_2/PAN*	\\	\\	0.21	\\	\\	1352	0.1 M mixed salt solution	[28]
UiO-66	0.56	0.35	0.32	7.4	4.6	4.3	0.1 M single salt solution	[29]
UiO-66	0.56	0.27	0.15	13	8.3	5.4	0.1 M mixed salt solution	[29]

Supplementary Table S4. Summary of ion separation performance (continued). Both concentration-driven diffusion dialysis and electrodialysis data are included.

Membrane	Ion permeation rate ($mol \cdot m^{-2} \cdot h^{-1}$)			Permselectivity			Feed solution	Ref
	K^+	Na^+	Li^+	K^+/Mg^{2+}	Na^+/Mg^{2+}	Li^+/Mg^{2+}		
DB15C5@ UiO-66	0.71	0.37	0.31	20	10	8.6	0.1 M single salt solution	29
DB15C5@ UiO-66	0.85	0.42	0.27	32	15	9.8	0.1 M mixed salt solution	29
DB18C6@ UiO-66	0.95	0.53	0.32	27	15	9.3	0.1 M single salt solution	29
DB18C6@ UiO-66	1.2	0.44	0.32	57	21	13	0.1 M mixed salt solution	29
CMP@100-20c	0.11	0.09	0.08	1.67	1.43	1.27	0.01 M single salt solution	30
i-CMP	0.08	0.04	0.03	40.4	20.9	17.2	0.01 M single salt solution	31
QAIPA-20	0.47	1.12	0.33	24.8	41.3	8	0.1 M mixed salt solution	32
ICM_{Q/S_35}	2.23	1.71	1.20	7.91	6.19	4.61	0.1 M mixed salt solution	33
$TpBDMe_2$	0.20	0.17	0.05	765	680	217	0.1 M single salt solution	34
$TpBDMe_2$	0.21	0.13	0.04	213	96.2	35.8	0.1 M mixed salt solution	34
Graphene	0.66	0.25	\	15.6	5.98	\	0.5 M single salt solution	35
N-doped graphene	0.10	0.03	\	111	27.8	\	0.1 M single salt solution	36
GO-PPD	0.09	0.06	\	7.14	4.87	\	0.1 M mixed salt solution	37
GO-PEI(70k)	0.20	0.13	\	44.9	42.3	\	0.5 M mixed salt solution	38
GO	2.50	2.50	\	1.20	1.20	\	0.2 M single salt solution	39
FGOM-1	0.06	0.04	\	9.70	6.50	\	0.1 M single salt solution	40
FGOM-15	0.004	0.003	\	11.0	10.3	\	0.1 M single salt solution	40
FGOM-30	0.002	0.001	\	50.0	31.1	\	0.1 M single salt solution	40
FGOM-60	0.0009	0.0004	\	90.3	40.3	\	0.1 M single salt solution	40
GO	\	0.83	0.32	\	3.77	1.45	0.1 M single salt solution	41
KCl-controlled GO	\	0.48	0.04	\	36.9	3.15	0.1 M single salt solution	41
rGO	0.19	0.08	0.02	169	48	12	0.1 M mixed salt solution	42
PET	0.002	0.003	0.036	39.6	60.6	634	1 M mixed salt solution	43
PET	0.034	0.015	0.014	16.7	20.9	21.2	1 M mixed salt solution	44
CC3 membrane	0.87	0.10	0.08	160	120	100	0.1 M mixed salt solution	45

Supplementary Table S4. Summary of ion separation performance (continued). Both concentration-driven diffusion dialysis and electrodialysis data are included.

Membrane	Ion permeation rate ($mol{\cdot}m^{-2}{\cdot}h^{-1}$)			Permselectivity			Feed solution	Ref
	K^+	Na^+	Li^+	K^+/Mg^{2+}	Na^+/Mg^{2+}	Li^+/Mg^{2+}		
MXene	0.004	0.005	0.066	1.72	2.10	26.7	0.2 M mixed salt solution	[46]
Mxene	0.94	1.53	1.40	5.88	9.56	8.75	0.2 M single salt solution	[47]
Mxene/GO	0.02	0.01	0.06	\	\	\	0.2 M single salt solution	[48]

Supplementary Table S5. Energy barrier analysis.

Cations	Energy barrier for partition[a] (kJ mol^{-1})	Energy barrier for Migration and Diffusion[b] (kJ mol^{-1})	Activation energy[c] (kJ mol^{-1})	Hydration free energy [d] (kJ mol^{-1})	Binding energy[e] (kJ mol^{-1})
**K^+**	41.4	~62	13.6	-295	-65.6
**Na^+**	40.0	~50	11.9	-365	-90.7
**Li^+**	37.4	~90	15.9	-475	-113.9
**Mg^{2+}**	71.0	~183	18.2	-1830	-130.3

[a]Energy barrier for ion partitioning into micropores, calculated as the maximum free energy in the first stage.
[b]Energy barrier for ion migration and diffusion in membrane, calculated as the difference between the maximum free energy and the minimum energy in the second stage.
[c]Activation energy measured in temperature-dependent conductivity measurements.
[d]Hydration free energy of ions.
[e]Binding energies between ions and AO groups in PIM membranes calculated by DFT.

Supplementary Table S6. Nominal composition of hypersaline brine from salt-lake reservoirs in China (diluted 2 times) and product solution after one-stage electrodialysis process.

	**Composition (g L^{-1})**						
**Sample**	Li^+	Na^+	K^+	Mg^{2+}	Cl^-	SO_4^{2-}	Mg/Li ratio
Salt lake salt solution	0.155	28.15	2.20	10.10	67.10	17.05	65
Product solution	1.17	15.23	2.92	2.65			2.26

Supplementary Table S7. Nominal composition of a typical salt-lake brine in China (after removal of Na^+ and K^+) and product solution after one-stage electrodialysis process.

	**Composition (g L^{-1})**					
**Sample**	Li^+	Na^+	K^+	Ca^{2+}	Mg^{2+}	Mg/Li ratio
Salt lake salt solution	3.38	0.125	0.414	1.520	106.74	31.6
Product solution	2.69	0.14	0.71	0.13	0.34	0.126

Supplementary Table S8. Setting of hydrated polymer models.

**Polymer types**	**Number of monomers**	**Number of water molecules**	**Number of cations/anions**			
			KCl	NaCl	LiCl	$MgCl_2$
**AO-PIM-1**	150	1321	24/24	24/24	24/24	24/48
**AO-PIM-De**	150	2852	-	-	351/51	-
**AO-PIM-Et**	150	974	-	-	18/18	-

References

1. Li, Z. et al. A Microporous Polymer with Suspended Cations for Anion Exchange Membrane Fuel Cells. *Macromolecules* **53**, 10998-11008 (2020).
2. Du, N., Song, J., Robertson, G.P., Pinnau, I. & Guiver, M.D. Linear High Molecular Weight Ladder Polymer via Fast Polycondensation of 5,5',6,6'-Tetrahydroxy-3,3,3',3'-tetramethylspirobisindane with 1,4-Dicyanotetrafluorobenzene. *Macromolecular Rapid Communications* **29**, 783-788 (2008).
3. Patel, H.A. & Yavuz, C.T. Noninvasive functionalization of polymers of intrinsic microporosity for enhanced CO_2 capture. *Chemical Communications* **48**, 9989-9991 (2012).
4. Baran, M.J. et al. Diversity-oriented synthesis of polymer membranes with ion solvation cages. *Nature* **592**, 225-231 (2021).
5. Huang, F. et al. Preparation of Amidoxime Polyacrylonitrile Chelating Nanofibers and Their Application for Adsorption of Metal Ions. *Materials (Basel)* **6**, 969-980 (2013).
6. Mizrahi Rodriguez, K. et al. Facile and Time-Efficient Carboxylic Acid Functionalization of PIM-1: Effect on Molecular Packing and Gas Separation Performance. *Macromolecules* **53**, 6220-6234 (2020).
7. Wang, A. et al. Selective ion transport through hydrated micropores in polymer membranes. *Nature* **635**, 353-358 (2024).
8. Perrin, J.-C., Lyonnard, S. & Volino, F. Quasielastic Neutron Scattering Study of Water Dynamics in Hydrated Nafion Membranes. *The Journal of Physical Chemistry C* **111**, 3393-3404 (2007).
9. Foglia, F. et al. Disentangling water, ion and polymer dynamics in an anion exchange membrane. *Nature Materials* **21**, 555-563 (2022).
10. Abbott, L.J., Hart, K.E. & Colina, C.M. Polymatic: a generalized simulated polymerization algorithm for amorphous polymers. *Theoretical Chemistry Accounts* **132**, 1334 (2013).
11. Ye, C. et al. Development of efficient aqueous organic redox flow batteries using ion-sieving sulfonated polymer membranes. *Nature Communications* **13**, 3184 (2022).
12. Thompson, A.P. et al. LAMMPS - a flexible simulation tool for particle-based materials modeling at the atomic, meso, and continuum scales. *Computer Physics Communications* **271**, 108171 (2022).
13. Jorgensen, W.L., Maxwell, D.S. & Tirado-Rives, J. Development and Testing of the OPLS All-Atom Force Field on Conformational Energetics and Properties of Organic Liquids. *Journal of the American Chemical Society* **118**, 11225-11236 (1996).
14. Li, P., Roberts, B.P., Chakravorty, D.K. & Merz, K.M., Jr. Rational Design of Particle Mesh Ewald Compatible Lennard-Jones Parameters for +2 Metal Cations in Explicit Solvent. *Journal of Chemical Theory and Computation* **9**, 2733-2748 (2013).
15. Li, P., Song, L.F. & Merz, K.M., Jr. Systematic Parameterization of Monovalent Ions Employing the Nonbonded Model. *Journal of Chemical Theory and Computation* **11**, 1645-1657 (2015).
16. Jorgensen, W.L., Chandrasekhar, J., Madura, J.D., Impey, R.W. & Klein, M.L. Comparison of simple potential functions for simulating liquid water. *The Journal of Chemical Physics* **79**, 926-935 (1983).
17. Andersen, H.C. Rattle: A "velocity" version of the shake algorithm for molecular dynamics calculations. *Journal of Computational Physics* **52**, 24-34 (1983).
18. Grossfield, A. WHAM: the weighted histogram analysis method, version 2.0.10. *http://membrane.urmc.rochester.edu/wordpress/?page_id=126*.

19. Humphrey, W., Dalke, A. & Schulten, K. VMD: Visual molecular dynamics. *Journal of Molecular Graphics* **14**, 33-38 (1996).
20. Frisch, M.J. et al. Gaussian 16 Rev. C.01. (2016).
21. Willems, T.F., Rycroft, C.H., Kazi, M., Meza, J.C. & Haranczyk, M. Algorithms and tools for high-throughput geometry-based analysis of crystalline porous materials. *Microporous and Mesoporous Materials* **149**, 134-141 (2012).
22. Tan, R. et al. Hydrophilic microporous membranes for selective ion separation and flow-battery energy storage. *Nature Materials* **19**, 195-202 (2020).
23. Tekinalp, Ö., Zimmermann, P., Holdcroft, S., Burheim, O.S. & Deng, L. Cation Exchange Membranes and Process Optimizations in Electrodialysis for Selective Metal Separation: A Review. *Membranes* **13**, 566 (2023).
24. Wang, R. & Lin, S. Membrane Design Principles for Ion-Selective Electrodialysis: An Analysis for Li/Mg Separation. *Environmental Science & Technology* **58**, 3552-3563 (2024).
25. Wong, T. et al. Sulfonated poly(ether-ether-ketone) membranes with intrinsic microporosity enable efficient redox flow batteries for energy storage. *Joule* **9**, 101795 (2025).
26. Li, Z. et al. Lithium extraction from brine through a decoupled and membrane-free electrochemical cell design. *Science* **385**, 1438-1444 (2024).
27. Tian, C. et al. Recycling of end-of-life polymeric membranes for water treatment: Closing the loop. *Journal of Membrane Science Letters* **3**, 100063 (2023).
28. Meng, Q.-W. et al. Enhancing ion selectivity by tuning solvation abilities of covalent-organic-framework membranes. *Proceedings of the National Academy of Sciences* **121**, e2316716121 (2024).
29. Xu, T. et al. Perfect confinement of crown ethers in MOF membrane for complete dehydration and fast transport of monovalent ions. *Science Advances* **10**, eadn0944 (2024).
30. Zhou, Z. et al. Precise Sub-Angstrom Ion Separation Using Conjugated Microporous Polymer Membranes. *ACS Nano* **15**, 11970-11980 (2021).
31. Zhou, Z. et al. Flexible Ionic Conjugated Microporous Polymer Membranes for Fast and Selective Ion Transport. *Adv. Funct. Mater.* **32**, 2108672 (2022).
32. Ul Afsar, N. et al. Zwitterion membranes for selective cation separation via electrodialysis. *Sep. Purif. Technol.* **254**, 117619 (2021).
33. Zhang, Y. et al. Highly efficient monovalent ion transport enabled by ionic crosslinking-induced nanochannels. *AIChE J.* **68**, e17825 (2022).
34. Sheng, F. et al. Efficient Ion Sieving in Covalent Organic Framework Membranes with Sub-2-Nanometer Channels. *Adv. Mater.* **33**, e2104404 (2021).
35. Li, S., Lee, J.-H., Hu, Q., Oh, T.-S. & Yoo, J.-B. Scalable graphene composite membranes for enhanced ion selectivity. *J. Membr. Sci.* **564**, 159-165 (2018).
36. Song, J., Yu, H., Ham, M. & Kim, I.S. Tunable Ion Sieving of Graphene Membranes through the Control of Nitrogen-Bonding Configuration. *Nano Lett.* **18**, 5506-5513 (2018).
37. Jia, Z., Wang, Y., Shi, W. & Wang, J. Diamines cross-linked graphene oxide free-standing membranes for ion dialysis separation. *J. Membr. Sci.* **520**, 139-144 (2016).
38. Zhang, W. et al. Graphene oxide membrane regulated by surface charges and interlayer channels for selective transport of monovalent ions over divalent ions. *Sep. Purif. Technol.* **291**, 120938 (2022).
39. Joshi, R.K. et al. Precise and ultrafast molecular sieving through graphene oxide membranes. *Science* **343**, 752-754 (2014).
40. Qian, Y. et al. Enhanced Ion Sieving of Graphene Oxide Membranes via Surface Amine Functionalization. *J. Am. Chem. Soc.* **143**, 5080-5090 (2021).

41. Liang, S., Wang, S., Chen, L. & Fang, H. Controlling interlayer spacings of graphene oxide membranes with cationic for precise sieving of mono-/multi-valent ions. *Sep. Purif. Technol.* **241**, 116738 (2020).
42. Xi, Y. et al. Graphene-based membranes with uniform 2D nanochannels for precise sieving of mono-/multi-valent metal ions. *J. Membr. Sci.* **550**, 208-218 (2018).
43. Wen, Q. et al. Highly Selective Ionic Transport through Subnanometer Pores in Polymer Films. *Adv. Funct. Mater.* **26**, 5796-5803 (2016).
44. Wang, P. et al. Ultrafast ion sieving using nanoporous polymeric membranes. *Nat. Commun.* **9**, 569 (2018).
45. Xu, T. et al. Highly Ion-Permselective Porous Organic Cage Membranes with Hierarchical Channels. *J. Am. Chem. Soc.* **144**, 10220-10229 (2022).
46. Lu, Z., Wu, Y., Ding, L., Wei, Y. & Wang, H. A Lamellar MXene ($Ti_3C_2T_x$)/PSS Composite Membrane for Fast and Selective Lithium-Ion Separation. *Angew. Chem. Int. Ed.* **60**, 22265-22269 (2021).
47. Ren, C.E. et al. Charge- and Size-Selective Ion Sieving Through $Ti_3C_2T_x$ MXene Membranes. *J. Phys. Chem. Lett.* **6**, 4026-4031 (2015).
48. Yin, Z. et al. Supported MXene/GO Composite Membranes with Suppressed Swelling for Metal Ion Sieving. *Membranes* **11**, 621 (2021).
